# Deep Sequencing-Based Identification of Small Regulatory RNAs in *Synechocystis* sp. PCC 6803

**DOI:** 10.1371/journal.pone.0092711

**Published:** 2014-03-19

**Authors:** Wen Xu, Hui Chen, Chen-Liu He, Qiang Wang

**Affiliations:** 1 Key Laboratory of Algal Biology, Institute of Hydrobiology, Chinese Academy of Sciences, Wuhan, Hubei Province, China; 2 University of Chinese Academy of Sciences, Beijing, China; Louisiana State University and A & M College, United States of America

## Abstract

*Synechocystis* sp. PCC 6803 is a genetically tractable model organism for photosynthesis research. The genome of *Synechocystis* sp. PCC 6803 consists of a circular chromosome and seven plasmids. The importance of small regulatory RNAs (sRNAs) as mediators of a number of cellular processes in bacteria has begun to be recognized. However, little is known regarding sRNAs in *Synechocystis* sp. PCC 6803. To provide a comprehensive overview of sRNAs in this model organism, the sRNAs of *Synechocystis* sp. PCC 6803 were analyzed using deep sequencing, and 7,951,189 reads were obtained. High quality mapping reads (6,127,890) were mapped onto the genome and assembled into 16,192 transcribed regions (clusters) based on read overlap. A total number of 5211 putative sRNAs were revealed from the genome and the 4 megaplasmids, and 27 of these molecules, including four from plasmids, were confirmed by RT-PCR. In addition, possible target genes regulated by all of the putative sRNAs identified in this study were predicted by IntaRNA and analyzed for functional categorization and biological pathways, which provided evidence that sRNAs are indeed involved in many different metabolic pathways, including basic metabolic pathways, such as glycolysis/gluconeogenesis, the citrate cycle, fatty acid metabolism and adaptations to environmentally stress-induced changes. The information from this study provides a valuable reservoir for understanding the sRNA-mediated regulation of the complex physiology and metabolic processes of cyanobacteria.

## Introduction

Cyanobacteria are among the oldest life forms on the earth, appearing in the fossil record as long as 3.5 billion years ago [1]. *Synechocystis* sp. PCC 6803 was the first photosynthetic organism for which the genome was completely sequenced [2]. This organism’s genomic information coupled with the rich biochemistry and physiological information available for *Synechocystis* sp. PCC 6803 has made this strain one of the most popular genetically tractable model organisms for physiological studies of photosynthesis and systems biology and an opportune biological model for biofuel-producing microalgae [3]–[5].

Non-coding RNAs are known to be key genetic regulators in organisms from all domains of life. Bacteria, as well as eukaryotes, possess a significant number of non-coding RNAs. In bacteria, these regulatory RNAs are generally referred to as small regulatory RNAs (sRNAs) because they typically range from 50 to 200 nt in length [6]. Over the past few years, considerable progress has been made in understanding the mechanisms and regulation of bacterial sRNAs [7]. A variety of RNA-based regulatory mechanisms have been proven to control a variety of processes, including stress responses, metabolic pathways, developmental processes and pathogenesis [8]–[12]. In recent years, deep sequencing has emerged as a new and powerful experimental method for transcriptome analysis [13], and sRNAs have been detected in a wide variety of microbial genomes, including those of *Escherichia coli* [14], *Mycobacterium tuberculosis* [15], *Streptococcus pneumoniae* [16], *Bacillus subtilis* [17] and *Helicobacter pylori* [18],*Nodularia spumigena* CCY9414[19],*Anabaena* sp. PCC 7120 [20].

As in eukaryotes, the importance of sRNAs as mediators of a number of cellular processes has begun to be recognized in bacteria [9], [21]–[26]. However, apart from *E. coli*, reports about sRNAs in other bacteria are very limited, and relatively little information on sRNAs in cyanobacteria is available. Since the first cyanobacterial antisense RNA (asRNA) was found in the filamentous strain *Calothrix* sp. PCC7601 [27], the repertoire of cyanobacterial sRNAs has been expanding. In 1997, Watanabe et al. identified a single sRNA, called 6Sa RNA, using an elaborate biochemical protocol in the freshwater cyanobacteria *Synechococcus* sp. PCC6301 [28], which later turned out to be the cyanobacterial homolog of the 6S RNA [29]. Another functional RNA studied early on in cyanobacteria was the RNA subunit of RNaseP from the unicellular cyanobacterium *Synechocystis* sp. PCC 6803 and the heterocyst-forming strains *Anabaena* sp. PCC 7120 and *Calothrix* sp. PCC 7601 [30]–[33]. However, the first genome-wide and systematic screen for ncRNAs in cyanobacteria was based on comparative genome analyses in four marine cyanobacteria, *Prochlorococcus* MED4, MIT 9313, SS120 and *Synechococcus* WH 8102, and followed by experimental validation, which identified seven different ncRNAs, called Yfr1-7 for cYanobacterial Functional RNA [34]. Among these ncRNAs, Yfr7 is homologous to the 6S RNA [35] and well-known for its global regulatory functions in bacteria [29], [36]. In *Synechocystis* sp. PCC 6803, the Yfr2 sRNA family consists of three members known as Yfr2a–c [37]. In addition, 12 new ncRNAs and 24 asRNAs have been found in *Prochlorococcus* sp. MED4 by using high-density microarrays and genome information [38]. Comparative genome analyses have also been used for the identification of sRNAs in four model cyanobacteria, including three unicellular model cyanobacteria, *Synechocystis* sp. PCC 6803, *Synechococcus elongatus* PCC6301 and *Thermosynechococcus elongatus* BP1, and the toxic *Microcystis aeruginosa* NIES843 [37]. Seventy-eight putative RNA elements were predicted as small RNA candidates in *Synechocystis* sp. PCC 6803, and experimental analyses validated two new sRNAs originating from the *fabF-hoxH* and *apcC-prmA* intergenic spacers and three highly expressed sRNAs belonging to the Yfr2 family of sRNAs [37].

A computational search and a tiling microarray covering approximately 40% of the chromosome by overlapping probes have been developed specifically to detect asRNAs in *Synechocystis* sp. PCC 6803. Sixty intergenic sRNAs and 73 asRNAs were detected. Among these RNAs, there were 54 new sRNAs and 72 new asRNAs [39]. Recently, a differential RNA-sequencing approach examined the global transcriptional profile of *Synechocystis* sp. PCC 6803, resulting in the identification of 314 sRNA candidates in intergenic regions [40]. In addition to intergenic sRNAs and asRNAs, bacteria also contain regulatory elements within the 5′-leader regions of several mRNAs [14]. In this study, we report novel sRNAs identified in *Synechocystis* sp. PCC 6803 using a deep sequencing analysis focusing on low-molecular-weight RNAs (≤ 200 nt). By applying this approach, 5211 novel transcribed sRNA regions were identified, and 589 were found expressed from plasmid regions. The expression of 11 intergenic sRNAs, nine antisense sRNAs and seven 5′-leader regions was confirmed by RT-PCR. Possible target genes regulated by all identified putative sRNAs were predicted by IntaRNA and further analyzed for functional categorizations for biological pathways. Overall, our results provide new insights towards understanding the complex regulatory network of sRNAs in *Synechocystis* sp. PCC 6803.

## Materials and Methods

### Sample preparation

Liquid cultures of wild-type *Synechocystis* sp. PCC 6803 were grown at 30°C in BG11 medium under continuous illumination with white light (30 μmol photons m^−2^ s^−1^). Cells were grown to an OD_730_ of 0.8 and harvested by centrifugation at 3000 g, then flashed frozen in liquid nitrogen and stored at –80°C until use.

### RNA preparation, library construction, and sequencing

Total RNA from *Synechocystis* sp. PCC 6803 cultures grown in 100 ml liquid medium was isolated using the TRIzol reagent (INVITROGEN, Carlsbad, CA, USA) according to the manufacturer’s protocol. Genomic DNA was digested by treatment with DNase (TAKARA, DALIAN, CA, CHINA), and rRNAs were removed using the Ribo-Zero rRNA Removal (Gram-negative bacteria) kit (EPICENTRE, Madison, WI, USA). Preparation of small RNA libraries was performed using the TruSeq Small RNA Sample Prep kit (ILLUMINA, San Diego, CA, USA) following the manufacturer’s instructions. Briefly, 1 μg of total RNA was ligated with adapters at the 3′ and 5′ ends without any size fractionation. Adapter-ligated RNA was reverse-transcribed with SuperScript II Reverse Transcriptase (INVITROGEN, Carlsbad, CA, USA) and then PCR-amplified for 10 cycles. Libraries (transcript sizes ≤ 200 nt) were purified using 6% TBE PAGE gels after electrophoresis. Library quality and concentration were evaluated using the Agilent 2100 Bioanalyzer DNA 1000 assay. The obtained cDNAs were sequenced using an Illumina Genome Analyzer IIx. The deep sequencing data have been submitted to the NCBI Sequence Read Archive with the accession number SRR935472.

### Analysis of the deep sequencing data and prediction of candidate sRNAs

After removing adaptor sequences from the raw reads obtained using Solexa sequencing technology and the low-quality sequences, the remaining sequencing reads were mapped onto the *Synechocystis* sp. PCC 6803 genome and its four megaplasmids using BWA [41]. A filtering procedure was implemented in PerlScript to extract the BWA output, and those reads that mapped onto any of the intergenic regions and regions that are complementary to known annotated genes were further analyzed. A cluster is defined as a genomic region that has mapped sequence reads with the same strand orientation contiguously overlapping with each other. Candidate sRNAs were extracted from all predicted transcribed regions (clusters) based on the following two criteria. First, the depth for a given transcribed region is more than 10. Second, the novel transcribed regions that were larger than 50 nt were extracted as candidate sRNAs.

### RT-PCR validation

Total RNA isolated using the TRIzol reagent (INVITROGEN, Carlsbad, CA, USA) was treated with RNase-free DNase I (FERMENTAS, Life Sciences, Germany) and two micrograms of total RNA was used as a template to synthesize first-strand cDNA using a One Step SYBR PrimeScript RT-PCR kit (TAKARA) following the manufacturer’s instructions. The sRNA primers were designed using Primer5. The detailed information for these primers is available in Online Resource Table S7 in File S1. The cycling parameters were: 95°C for 3 min followed by 35 cycles of 95°C for 15 s, 57°C for 30 s and 72°C for 30 s.

### Target prediction and the analysis of candidate sRNAs

To obtain the target information, we searched the target genes of all the putative sRNAs using the target prediction program IntaRNA taking the sRNAs as query and all annotated protein-coding genes of *Synechocystis* sp. PCC 6803 as the subject. GO annotations with the default parameters and GO enrichment with EASE scores of 0.05 were performed with the functional annotation tool DAVID [42], [43]. The KEGG pathway annotations were performed using the KEGG (Kyoto Encyclopedia of Genes and Genomes) pathway database (http://www.genome.jp/kegg/tool/map_pathway2.html) with the default parameters.

## Results

### A general strategy for the high-throughput identification of sRNAs

**Figure 1:**
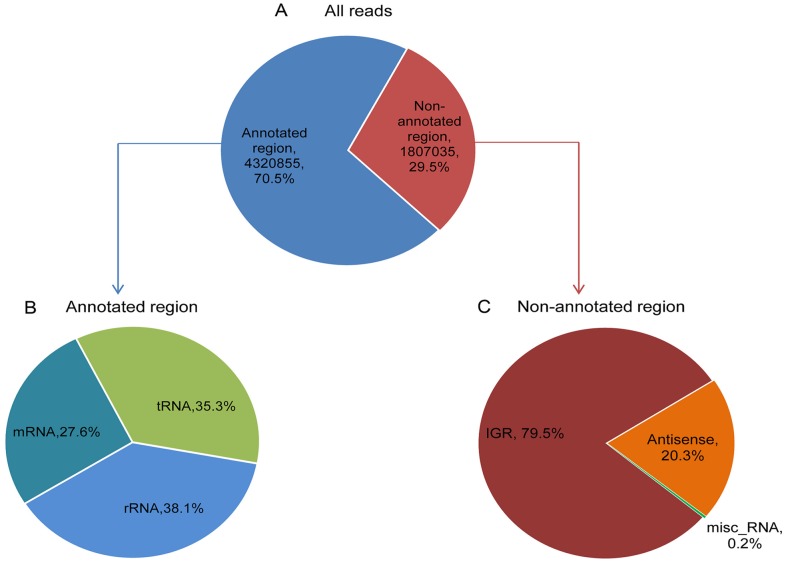
Pie charts classifying the deep sequencing reads. A, The percentages of all the deep sequencing reads mapped to the previously annotated or non-annotated regions of the *Synechocystis* sp. PCC 6803 genome. B, The relative proportions of the deep sequencing reads (n  =  4,320,855) mapped to the annotated regions. C, The relative percentages of the deep sequencing reads (n  =  1,807,035) mapped to the nonannotated regions. IGR, intergenic region; Antisense, reads located on the cis-antisense strand of a known gene; misc_rna, RNA of unknown function.

To comprehensively identify and characterize the sRNAs of wild-type *Synechocystis* sp. PCC 6803 using deep sequencing, a total of 7.95 million low-molecular-weight RNA (81 nt) reads were obtained from cultures harvested at exponential phase. From the raw sequences generated by high-throughput sequencing, the 3′- and 5′-adaptor sequences were trimmed off. Low-quality sequences were identified as those sequencing reads that averaged Phred scores smaller than 20 and sequencing reads shorter than 18 nucleotides. After discarding the low-quality reads, a total of 6.12 million reads could be successfully mapped onto the *Synechocystis* sp. PCC 6803 genome and its four megaplasmids. As ribosomal RNAs (rRNAs) account for the vast majority of total RNAs in a bacterial cell, but on account of the removal of rRNAs, these sequences represented only 26.9% of the total reads in our sample. As shown in Figure 1, 70.5% of the reads were mapped to annotated regions corresponding to rRNAs (38.1%), tRNAs (35.3%), and mRNAs (26.6%), whereas the remaining 29.5% were mapped to non-annotated regions corresponding to intergenic regions (79.5%), the cis-antisense strands of known genes (20.3%) and misc_rna (RNA of unknown function, 0.2%).

Generally, sRNAs can be generated either as primary transcripts or as being processed from longer precursor transcripts [44]. In the current study, the latter case was ignored because it was impossible to discriminate the processed sRNAs from RNA degradation products. To predict novel transcribed regions, all overlapping reads of mapped non-annotated regions were assembled, and 16,192 novel transcribed regions (clusters) were generated. It should be noted that 43 of the 89 BSRD (Bacterial Small regulatory RNA Database) registered sRNAs [45] were detected in the current study, indicating the reliability of our method.

The sequencing results were further analyzed for transcripts derived from intergenic regions and for reads complementary to known annotated genes. Transcript clusters were listed as putative candidate sRNAs only when they were represented by 10 or more reads at one cluster and were 50 nt longer. This procedure resulted in a total of 5211 candidate sRNAs, including the previously reported iron stress-repressed IsrR [9], PsbA3R [46], Yfr2a and Yfr2b [37].

### Identification of sRNAs and validation by RT-PCR

**Figure 2:**
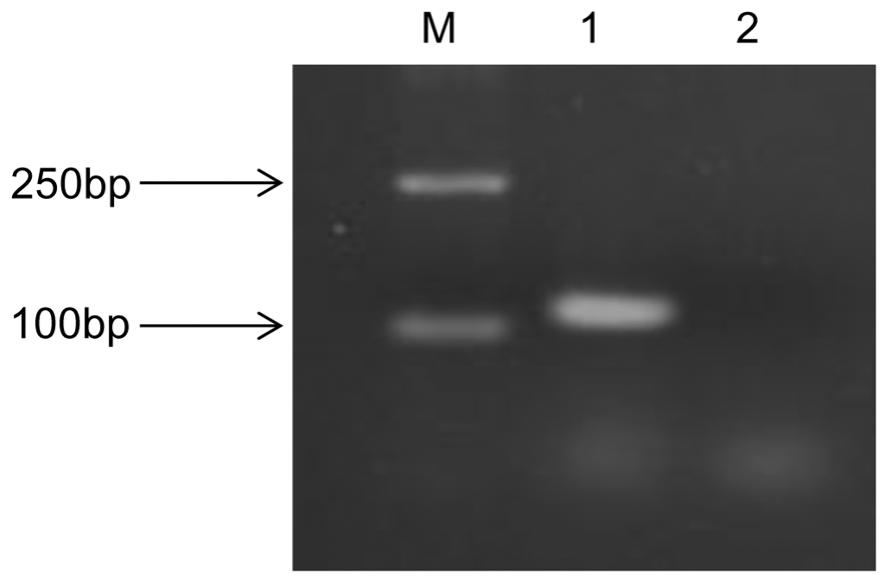
PCR results of total RNA with DNase I digestion. Electrophoresis of PCR amplicons of 5S rRNA using 2% agarose gels. M, Marker; 1, PCR amplicons of 5S rRNA in total RNA without digestion; 2, PCR amplicons of 5S rRNA in total RNA with DNase I digestion.

Among the 5211 candidate sRNAs resulting from the deep sequencing analysis, 4622 were located on the chromosome, and 589 were located on the four megaplasmids: 110 on pSYSX, 80 on pSYSG, 200 on pSYSA and 199 on pSYSM. The putative sRNA candidates were placed into three different classes: class I compromises intergenic sRNAs, which are located within intergenic regions without overlapping adjacent genes; class II are referred to as antisense RNAs (asRNAs), located antisense to known genes; and class III comprises 5′-leader regions of mRNA transcripts, including riboswitches. To rule out the possibility of DNA contamination in total RNA sample, total RNA was digested with DNase I before reverse transcription and the results of 5S rRNA amplification showed that there was no DNA contamination after digestion in total RNA (Fig. 2)

#### Class I, Intergenic small RNAs

**Figure 3:**
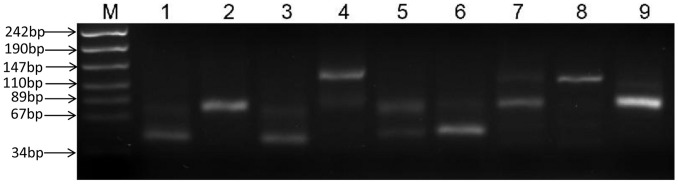
Validation of intergenic sRNA expression by RT-PCR. Electrophoresis of the PCR amplicons of the 9 selected intergenic sRNAs using 3% agarose gels. Lane M: Marker, Lane 1-9: SYPCC_Igr_01, SYPCC_Igr_02, SYPCC_Igr_03, SYPCC_Igr_04, SYPCC_Igr_05, SYPCC_Igr_06, SYPCC_Igr_07 SYPCC_Igr_08, and SYPCC_Igr_09.

An investigation that examined the global transcriptional profile of *Synechocystis* sp. PCC 6803 using RNA-seq resulted in the identification of 314 sRNA candidates in intergenic regions [40]. However, by using Solexa sequencing, we searched for cDNA clusters that occurred specifically within intergenic regions and discovered 423 clusters that represent possible intergenic sRNA candidates, including 335 on the chromosome and 88 on the four megaplasmids (Online Resource Table S1 in File S1). Twelve of the 56 putative top-scoring sRNAs identified by RNA-seq [40] are also presented in our results, and a total of 411 novel sRNA candidates are listed (Online Resource Table S1 in File S1). The expression of 9 out of the 13 intergenic candidate sRNAs could be detected by RT-PCR (Fig. 3), and the results are summarized in Table 1. The length of each main band on the SYPCC_igr_02, SYPCC_igr_07, SYPCC_igr_08 and SYPCC_igr_09 were almost as predicted (Fig. 3), whereas the other five candidate sRNAs tested exhibited more than one band and appeared to be different in size.

**Table 1:** The 9 selected candidate intergenic sRNAs for RT-PCR validation.

sRNA name	Location	Start (nt)	End (nt)	Strand
SYPCC_Igr_01	pSYSA	68081	68925	+
SYPCC_Igr_02	chromosome	2530895	2530962	+
SYPCC_Igr_03	pSYSG	15392	15481	+
SYPCC_Igr_04	chromosome	496101	496321	+
SYPCC_Igr_05	chromsome	1104709	1104929	+
SYPCC_Igr_06	chromosome	3019919	3020058	–
SYPCC_Igr_07	pSYSM	66503	66574	+
SYPCC_Igr_08	chromosome	3039292	3039406	+
SYPCC_Igr_09	chromosome	3305423	3305514	+

#### Class II, Antisense RNAs

**Figure 4:**
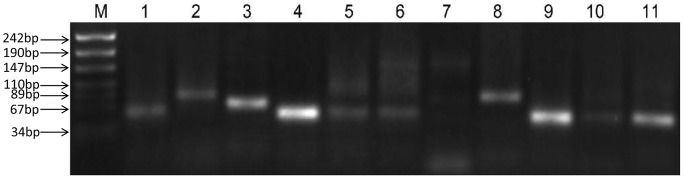
Validation of asRNA expression by RT-PCR. Electrophoresis of the PCR amplicons of 11 asRNAs using 3% agarose gels. Lane M: Marker, Lane 1–11: SYPCC_anti_01, SYPCC_anti_02, SYPCC_anti_03, SYPCC_anti_04, SYPCC_anti_05, SYPCC_anti_06, SYPCC_anti_07, SYPCC_anti_08, SYPCC_anti_09, SYPCC_anti_10, and SYPCC_anti_11.

It has been reported that, in the unicellular cyanobacterium *Synechocystis* sp. PCC 6803, asRNAs could account for up to 26% of all genes [39], [40] and that one gene may be predicted for several candidate sRNAs, such as *slr1028* and *slr0261* [40]. Overall, 4627 candidate asRNAs, including 15 of the asRNAs identified via tilling array and Northern blot analysis by Georg et al. [39], were suggested by our analysis, and the top-scoring (≥ 40 reads) putative asRNAs are provided in Online Resource Table S2 in File S1. Among these asRNAs, 116 originate from the four plasmids. It should be noted that the three previously reported known asRNAs in *Synechocystis* sp. PCC 6803, as1_flv4 [47], IsrR [9] and PsbA3R [46], which have been demonstrated to regulate the expression of the *flv4-2* operon, iron stress-repressed mRNA *isiA* and the photosystem II D1 protein *psbA3*, respectively, were also detected in our analysis. The expression of 11 of the 14 candidate asRNAs was verified by RT-PCR (Fig. 4), and the results are summarized in Table 2.

**Table 2:** The 11 selected candidate asRNAs for RT-PCR validation.

sRNA name	Location	Start (nt)	End (nt)	Strand
SYPCC_anti_01	chromosome	407802	407923	+
SYPCC_anti_02	chromosome	1909216	1909443	–
SYPCC_anti_03	chromosome	2724266	2724365	–
SYPCC_anti_04	chromosome	279064	29161	–
SYPCC_anti_05	chromosome	346136	346282	+
SYPCC_anti_06	chromosome	2478723	2478824	–
SYPCC_anti_07	chromosome	940674	940896	+
SYPCC_anti_08	chromosome	1665302	1665520	+
SYPCC_anti_09	chromosome	2219904	2220106	–
SYPCC_anti_10	chromosome	1020080	1020302	–
SYPCC_anti_11	pSYSX	91564	91738	–

#### Class III, 5’-leader region

**Figure 5:**
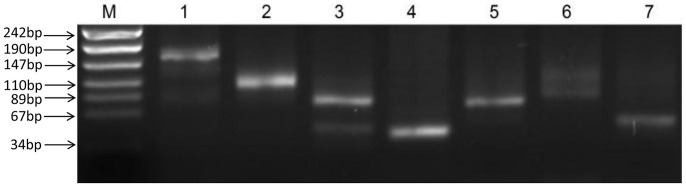
Validation of 5′-leader region expression by RT-PCR. Electrophoresis of PCR amplicons of 7 5′-leader regions using 3% agarose gels. Lane M: Marker, Lane 1-7: SYPCC_Ir_01, SYPCC_Ir_02, SYPCC_Ir_03, SYPCC_Ir_04, SYPCC_Ir_05, SYPCC_Ir_06, and SYPCC_Ir_07.

In addition to intergenic sRNAs and asRNAs, bacteria contain regulatory elements within their 5′-leader regions [14], [48], [49]. Similar to sRNAs, these regulatory elements control transcription elongation, mRNA stability, and translation initiation in response to specific stimuli and metabolites [49]. In *Synechocystis* sp. PCC 6803, the distances between the identified and annotated genes’ transcriptional start sites and start codons for protein-coding genes range from 0 to 278 nt with a median distance of 42 nt [40]. Therefore, we define those sRNA candidates upstream of the 5′ ORF that are smaller than 300 nt as functional 5′-leader region sRNAs. A total of 161 5′-leader region candidates are shown in Online Resource Table S3 in File S1. Yfr2b, the 5′-untranslated leader of *slr0199* [37], and the 6 other candidates reported by Mitschke et al. [40] were also detected in the current analysis. However, in contrast to the average length of 42 nt for the 5′-leader regions of the transcripts, the average length of predicted 5′-leader region candidates is ∼146 nt. This observation suggests that ‘long’ 5′-leader regions are likely to occur only when specialized functions are encrypted within them, and the most likely explanation for a long 5′-mRNA leader region is due to the inclusion of a signal-responsive regulatory RNA [17]. Seven out of 10 high read candidates selected for verification were detected by RT-PCR (Fig.5), and the results are summarized in Table 3.

**Table 3:** The 7 selected candidate 5′-leader regions for RT-PCR validation.

sRNA name	Location	Start (nt)	End (nt)	Strand
SYPCC_Ir_01	chromosome	1832226	1832339	+
SYPCC_Ir_02	chromosome	2730512	2730591	+
SYPCC_Ir_03	chromosome	3433172	3433275	+
SYPCC_Ir_04	chromosome	901005	901125	+
SYPCC_Ir_05	chromosome	458917	459162	+
SYPCC_Ir_06	chromosome	3168882	3169030	+
SYPCC_Ir_07	chromosome	102301	102387	–

### Functional categorization of candidate small RNA target genes

To better understand the role of sRNAs in different biological modules, sRNA- regulating target genes were predicted using IntaRNA [50]. Gene ontology (GO) assignment programs were utilized for the functional categorization of the predicted target genes, and the functional annotation tool DAVID [42], [43] was used to analysis the target GO annotation terms. The results are presented in Online Resource Table S4 in File S1.

**Figure 6:**
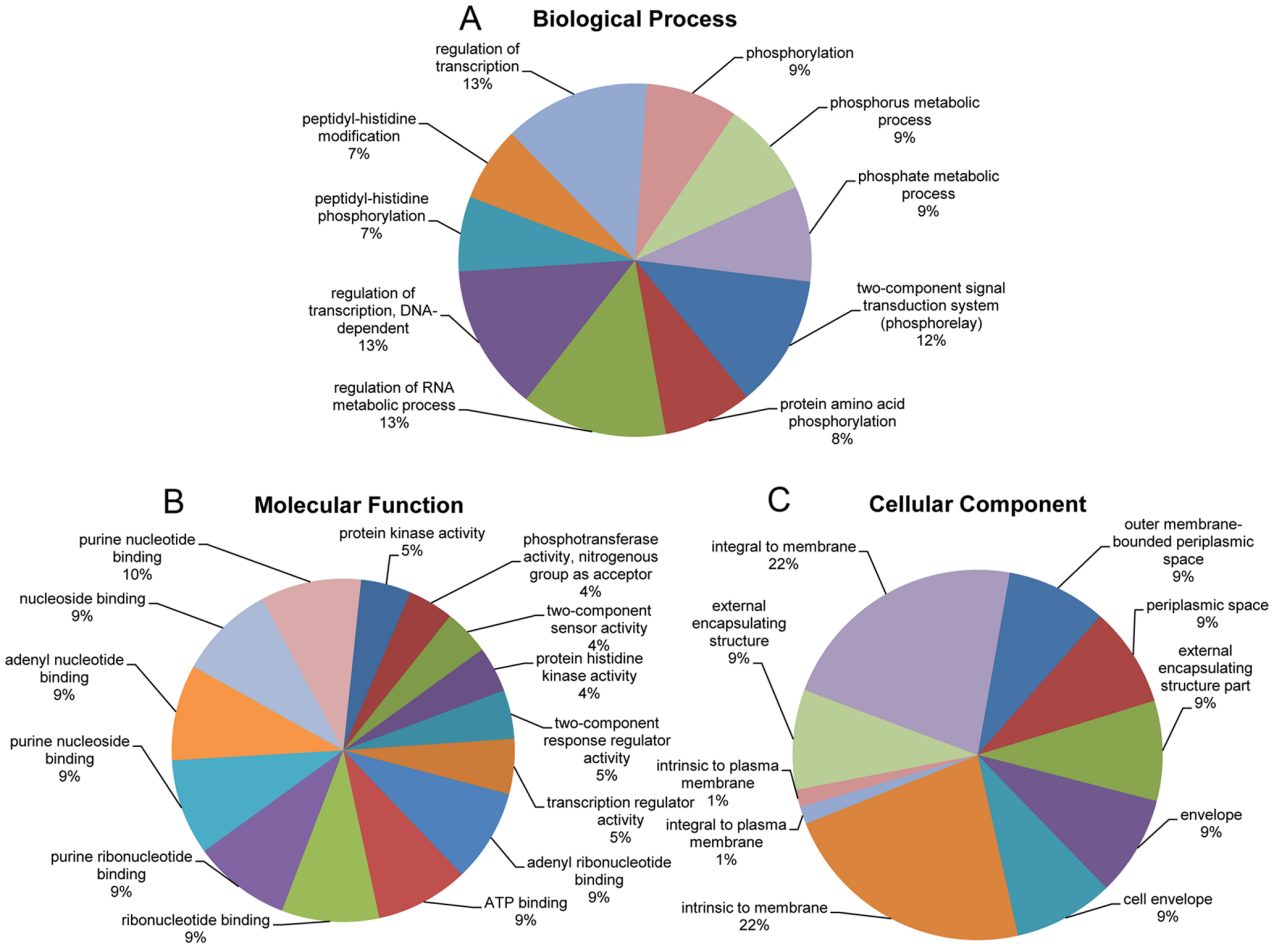
GO analyses of predicted target sRNA genes. GO analysis of target genes that are predicted to be involved in biological processes A, molecular functions B and their cellular components C. Classified target gene objects are depicted as percentages (in brackets) of the total number of gene objects with GO assignments.

Enriched GO terms were searched by GO enrichment analysis and defined by the functional significance of their target genes. The results are shown in Online Resource Table S5 in File S1. As shown in Figure 6A, the GO terms were distributed widely with regards to their respective biological processes, from transcription to RNA metabolite, to posttranslational modifications and signaling pathways. When classified as molecular functions, a total of 63% of the enriched GO terms are related to nucleoside and nucleotide binding, indicating extremely important roles for the sRNAs in the processes of replication and transcription (Fig. 6B). As for cellular components, the majority of the predicted targets are involved in different membrane systems (Fig. 6C).

To further evaluate the role of the identified possible target genes in biological/signaling pathways, the predicted target genes were superimposed onto the KEGG (Kyoto Encyclopedia of Genes and Genomes) pathway database (http://www.genome.jp/kegg/tool/map_pathway2.html). As shown in Online Resource Table S6 in File S1, the annotated genes were grouped into 89 known metabolic or signaling pathways, and many identified target genes are involved in pathways responsible for the control of key physiological processes. The pathways in which the target genes are predominantly enriched include “metabolic pathways” (62.8%), followed by “biosynthesis of secondary metabolites” (33%), “microbial metabolism in diverse environments” (14.3%), “two-component system” (8.8%), and “ABC transporters” (8.6%). Among those, 9 target genes (*sll0427, sll0629, sll1317, slr1181, slr1655, slr1739, slr1834, smr0001* and *ssr2831*) were assigned to photosynthesis.

### Small RNA targets involved in photosynthesis

The unicellular aquatic cyanobacterium *Synechocystis* sp. PCC 6803 has long been utilized as a model organism for photosynthesis studies. The regulation of photosynthesis genes in cyanobacteria has been studied in the past few years [9], [46]. According to the sRNA target data set contributed by this study, 9 genes were found to be involved in the photosynthetic pathway. As shown in Table 4, there are 4 genes involved in photosystem I and photosystem II, respectively. Among these genes, the D1 protein, encoded by the *psbA* gene, is believed to be the initial site of photoinhibition [51]. PsbT has been demonstrated to be involved in the efficient repair of photodamaged PSII under high light conditions [52], and there are also results indicating that PsaK and PsaL are essential for high light condition survival [53], [54]. Therefore, it is likely that the sRNAs in *Synechocystis* sp. PCC 6803 are involved in the regulation of the light energy distribution between PSI and PSII. Moreover, the PetA of the Cytb_6_f complex, which provides the electronic connection between PSII and PSI, was also identified. Taken together, sRNAs may be essential for the regulation of photosynthetic function, especially under high light conditions.

**Table 4:** The predicted target sRNA genes involved in photosynthesis.

Gene ID	Description
sll0427	psbO; photosystem II manganese-stabilizing polypeptide
sll0629	psaK; photosystem I subunit X
sll1317	petA; apocytochrome f
slr1181	psbA1; photosystem II D1 protein
slr1655	psaL; photosystem I reaction center protein subunit XI
slr1739	photosystem II protein
slr1834	psaA; photosystem I P700 chlorophyll a apoprotein A1
smr0001	psbT; photosystem II reaction center protein T
ssr2831	psaE; photosystem I reaction center subunit IV

## Discussion

Although sRNAs have been increasingly recognized as major modulators of gene expression in bacteria [9], [21], [46], [55], only a limited number of studies have sought to identify the global set of expressed sRNAs in cyanobacteria [34], [37], [39], [40], [56]. Here, we describe the deep sequencing-based identification of sRNAs in the model cyanobacterium *Synechocystis* sp. PCC 6803. We utilized the strategy of analyzing millions of short reads from deep sequencing for the prediction of expressed sRNAs. Although the deep sequencing analyses used in this study focused on identifying low-molecular-weight RNAs (≤ 200 nt) by limiting the lengths of the RNA samples, assemblages of contiguously overlapping reads also overlap with longer regulatory RNAs.

During the past decade, several groups have extensively searched for sRNAs in *Synechocystis* sp. PCC 6803 using comparative genome analyses and experimental approaches [37], [39], [40]. Therefore, we examined some overlaps between the candidate sRNAs in this study and those identified in previous studies. In this study, 5211 candidate sRNAs were identified from exponentially growing cells, 27 of which were validated by RT-PCR. A total of only 33 overlapping candidate sRNAs were identified. The possible reasons are as follows. (1) It is quite possible that some of the bioinformatics-predicted sRNAs are not expressed at all. (2) This result is also possible because the majority of sRNAs are only expressed under stress conditions and are typically very tightly regulated [7] and may not have been expressed in our exponentially grown healthy sample pool. (3) Additionally, different bioinformatics-based approaches, such as [34] and ‘orphan’ transcriptional signals [40], only rely on transcriptional start sites, which may have resulted in our different results. Comparative bioinformatics is limited to the discovery of sRNAs that are conserved in closely related species, whereas experimental approaches, such as deep sequencing, should be able to comprehensively discover sRNAs in bacteria.

Plasmids containing sRNAs have been reported in various bacterial species [17], [57], [58]. Our results demonstrate that the plasmids in *Synechocystis* sp. PCC 6803 also express sRNAs, and 4 of those, including 3 intergenic RNAs (Table 1) and 1 asRNA (Table 2), were verified by RT-PCR, which suggests plasmids containing sRNAs could be a common feature of prokaryotic sRNAs. In fact, one of these candidate sRNAs, a cluster coded in pSYSA, has been identified in more than 13,000 reads, agreeing with previously reported results from *Chlamydia trachomatis* [57]. In addition, some candidate sRNAs were found in the antisense orientation to known genes, implying a role in the regulation of plasmid-coded genes. However, the roles of plasmid-encoded sRNAs are less clear and have been reported to be involved in the control of plasmid replication [59] and plasmid copy number [60], [61] and are perhaps related to the regulation of chromosomal gene expression [62].

sRNAs can be generated either as primary transcripts or through their processing from longer precursor transcripts [44]. In the current study, some of the identified sRNAs were observed for more than one transcript (Fig. 3), suggesting a working mechanism for the sRNAs in *Synechocystis* sp. PCC 6803 that may also involve the post-transcriptional processing of the primary transcripts. More recently, two asRNAs, Psb2R and Psb3R, located in 5′-untranslated regions of *psbA2* and *psbA3* were validated as two distinct signals with fragment lengths of 129 and approximately 220 nucleotides and 157 and 181 nucleotides, respectively, in *Synechocystis* sp. PCC 6803 [46].

While there has been a rapid increase in the identification of bacterial sRNAs in recent years, the identification of sRNA targets has proceeded relatively slowly. In *E. coli,* the number of validated sRNAs is approximately 80; however, only approximately 20 sRNAs have been assigned cellular functions [14], [63]. However, small RNA-mediated post-transcriptional regulation has emerged as an important regulatory mechanism for gene expression in various cellular processes [64]. One small RNA concurrently regulates several target mRNAs by binding to the corresponding mRNA via either perfect or imperfect sequence complementarity or by modulating protein activities, such as the Hfq-dependent GcvB sRNA, which has been shown to target more than 20 different mRNAs [65]. By targeting mRNA transcripts or affecting the activities of proteins at one time, sRNAs regulate a wide range of metabolic processes, such as quorum sensing [66], two-component signal transduction systems [65], photosynthesis [9], responses to the environment [67] and the regulation of outer membrane porins [68]. In this study, we analyzed all putative target genes in the KEGG pathway database, and the targets of the sRNAs were shown to be involved in 89 known metabolic or signaling pathways, suggesting that these sRNAs might play a significant role in regulating different biological processes and pathways such as photosynthesis, responses to diverse environments and the biosynthesis of secondary metabolites. Therefore, this work provides a solid platform for further experimental analyses of sRNAs and for the identification of the biological functions of these specific sRNAs.

To summarize, we have comprehensively analyzed sRNA expression under normal growth conditions using *Synechocystis* sp. PCC 6803 and predicted 5211 candidate sRNAs. We also identified several novel sRNAs that are highly expressed in plasmid regions, and four of these, including three intergenic sRNAs and one asRNA, were verified experimentally. Though the functions for the novel specific sRNAs obtained in this study remain unknown, the reliability of the targets for all the predicted sRNAs was further analyzed for the functional categorization of the biological pathways. The results indicate that these sRNAs are indeed involved in various metabolic pathways. This current study also provides more information about the regulatory network of sRNAs and will advance our understanding of sRNA functions in cyanobacteria.

## Supporting Information

File S1 **Contains the Files: Table S1**. List of all predicted candidate intergenic small RNAs. **Table S2.** List of some putative top-scoring antisense RNAs(≥40 reads). **Table S3.** List of all predicted 5′ leader region candidates. **Table S4.** Classification of predicted targets of candidate small regulatory RNAs according to their biological process, molecular function and cellular component. **Table S5.** Complete list of enriched GO terms of target genes in *Synechocystis* sp. PCC 6803. **Table S6.** Complete list of KEGG pathways in the identified target genes of small regulatory RNAs. **Table S7.** List of the primer oligonucleotides used in this study.
(DOCX)
