# Supplementary material for: Deep Sequencing-Based Identification of Small Regulatory RNAs in Synechocystis sp. PCC 6803
Source: PLoS One. 2014 Mar 19;9(3):e92711. doi: 10.1371/journal.pone.0092711 (PMC3960264; doi:10.1371/journal.pone.0092711)
Supplement: File S1 — Contains the Files: Table S1. List of all predicted candidate intergenic small RNAs. Table S2. List of some putative top-scoring antisense RNAs(≥40 reads). Table S3. List of all predicted 5′ leader region candidates. Table S4. Classification of predicted targets of candidate small regulatory RNAs according to their biological process, molecular function and cellular component. Table S5. Complete list of enriched GO terms of target genes in Synechocystis sp. PCC 6803. Table S6. Complete list of KEGG pathways in the identified target genes of small regulatory RNAs. Table S7. List of the primer oligonucleotides used in this study. (DOCX) [file pone.0092711.s001.docx]

**File S1.**

| **Table S1.** List of all predicted candidate intergenic small RNAs. | |  |  |
| --- | --- | --- | --- |
| **Location** | **Start(nt)** | **End(nt)** | **Strand** |
| chromsome | 1886876 | 1886926 | - |
| chromsome | 1914760 | 1914812 | + |
| chromsome | 261769 | 261827 | - |
| chromsome | 3001757 | 3001817 | - |
| chromsome | 2024478 | 2024539 | - |
| chromsome | 1578624 | 1578687 | - |
| chromsome | 1728648 | 1728711 | + |
| chromsome | 3188100 | 3188164 | + |
| chromsome | 1606849 | 1606913 | + |
| chromsome | 2091640 | 2091705 | + |
| chromsome | 969686 | 969752 | - |
| chromsome | 2530895 | 2530962 | + |
| chromsome | 1302867 | 1302936 | + |
| chromsome | 923871 | 923940 | + |
| chromsome | 326045 | 326115 | - |
| chromsome | 3316970 | 3317040 | + |
| chromsome | 1104763 | 1104834 | - |
| chromsome | 3427247 | 3427319 | - |
| chromsome | 1977977 | 1978050 | + |
| chromsome | 1514053 | 1514127 | + |
| chromsome | 1949126 | 1949202 | - |
| chromsome | 1947209 | 1947285 | + |
| chromsome | 2312563 | 2312640 | + |
| chromsome | 2948242 | 2948320 | + |
| chromsome | 1921981 | 1922060 | - |
| chromsome | 3479108 | 3479187 | + |
| chromsome | 297935 | 298014 | + |
| chromsome | 424613 | 424692 | + |
| chromsome | 1429168 | 1429247 | + |
| chromsome | 1906091 | 1906170 | + |
| chromsome | 2842640 | 2842720 | - |
| chromsome | 2730183 | 2730264 | + |
| chromsome | 2093512 | 2093594 | + |
| chromsome | 3093048 | 3093130 | + |
| chromsome | 779979 | 780062 | - |
| chromsome | 2008778 | 2008862 | - |
| chromsome | 1607966 | 1608050 | - |
| chromsome | 2509648 | 2509733 | - |
| chromsome | 1650538 | 1650623 | + |
| chromsome | 1428030 | 1428116 | + |
| chromsome | 416264 | 416351 | - |
| chromsome | 3002819 | 3002906 | - |
| chromsome | 1135182 | 1135269 | - |
| chromsome | 1303175 | 1303262 | + |
| chromsome | 1002253 | 1002340 | + |
| chromsome | 456853 | 456941 | - |
| chromsome | 2706017 | 2706105 | + |
| chromsome | 3039423 | 3039512 | + |
| chromsome | 943969 | 944059 | - |
| chromsome | 783128 | 783218 | - |
| chromsome | 955697 | 955787 | - |
| chromsome | 1845424 | 1845514 | + |
| chromsome | 3305423 | 3305514 | + |
| chromsome | 2950783 | 2950874 | + |
| chromsome | 1668414 | 1668506 | - |
| chromsome | 1524247 | 1524340 | - |
| chromsome | 1832202 | 1832296 | - |
| chromsome | 2676363 | 2676457 | - |
| chromsome | 1763105 | 1763200 | + |
| chromsome | 1112091 | 1112186 | + |
| chromsome | 1751165 | 1751260 | + |
| chromsome | 291305 | 291403 | + |
| chromsome | 3317062 | 3317161 | + |
| chromsome | 3250872 | 3250972 | - |
| chromsome | 2859951 | 2860051 | + |
| chromsome | 2316968 | 2317068 | + |
| chromsome | 1166846 | 1166946 | + |
| chromsome | 781466 | 781567 | - |
| chromsome | 1318575 | 1318676 | + |
| chromsome | 430279 | 430380 | + |
| chromsome | 1899291 | 1899392 | + |
| chromsome | 1957611 | 1957713 | - |
| chromsome | 821807 | 821909 | + |
| chromsome | 1558253 | 1558355 | + |
| chromsome | 842645 | 842747 | + |
| chromsome | 3028201 | 3028304 | - |
| chromsome | 1945406 | 1945509 | + |
| chromsome | 2330378 | 2330482 | + |
| chromsome | 969424 | 969528 | + |
| chromsome | 3074791 | 3074895 | + |
| chromsome | 1728760 | 1728864 | + |
| chromsome | 727523 | 727628 | + |
| chromsome | 609043 | 609149 | + |
| chromsome | 875398 | 875506 | - |
| chromsome | 1112390 | 1112498 | - |
| chromsome | 1957499 | 1957607 | - |
| chromsome | 387959 | 388068 | - |
| chromsome | 2861865 | 2861974 | + |
| chromsome | 3305535 | 3305645 | + |
| chromsome | 3250612 | 3250723 | - |
| chromsome | 2695305 | 2695416 | + |
| chromsome | 2148134 | 2148246 | - |
| chromsome | 120173 | 120285 | - |
| chromsome | 2446255 | 2446367 | + |
| chromsome | 2910961 | 2911073 | + |
| chromsome | 3322999 | 3323112 | - |
| chromsome | 473838 | 473951 | - |
| chromsome | 794245 | 794358 | - |
| chromsome | 776401 | 776514 | + |
| chromsome | 1679396 | 1679510 | - |
| chromsome | 1592591 | 1592705 | - |
| chromsome | 1098078 | 1098192 | - |
| chromsome | 3305449 | 3305563 | - |
| chromsome | 3039292 | 3039406 | + |
| chromsome | 957926 | 958041 | - |
| chromsome | 1899425 | 1899540 | - |
| chromsome | 3458160 | 3458275 | - |
| chromsome | 2351144 | 2351260 | - |
| chromsome | 3452142 | 3452258 | + |
| chromsome | 2227446 | 2227562 | + |
| chromsome | 1002136 | 1002252 | + |
| chromsome | 2796908 | 2797025 | - |
| chromsome | 785230 | 785347 | + |
| chromsome | 2324901 | 2325018 | + |
| chromsome | 1240217 | 1240335 | - |
| chromsome | 901067 | 901186 | - |
| chromsome | 2263581 | 2263701 | - |
| chromsome | 2639478 | 2639598 | + |
| chromsome | 3549751 | 3549872 | - |
| chromsome | 2676230 | 2676351 | + |
| chromsome | 112708 | 112831 | + |
| chromsome | 1923844 | 1923967 | + |
| chromsome | 3016804 | 3016929 | - |
| chromsome | 2905317 | 2905442 | + |
| chromsome | 268483 | 268608 | + |
| chromsome | 1452569 | 1452694 | + |
| chromsome | 997806 | 997931 | + |
| chromsome | 2098608 | 2098734 | - |
| chromsome | 2328037 | 2328163 | - |
| chromsome | 1349959 | 1350085 | + |
| chromsome | 1600506 | 1600633 | - |
| chromsome | 1604530 | 1604657 | - |
| chromsome | 1561475 | 1561605 | - |
| chromsome | 3352450 | 3352581 | - |
| chromsome | 1244301 | 1244432 | - |
| chromsome | 51895 | 52026 | + |
| chromsome | 1728873 | 1729004 | + |
| chromsome | 983546 | 983678 | - |
| chromsome | 572972 | 573105 | + |
| chromsome | 821406 | 821539 | + |
| chromsome | 92670 | 92804 | - |
| chromsome | 477519 | 477653 | + |
| chromsome | 3048718 | 3048853 | + |
| chromsome | 1881415 | 1881551 | + |
| chromsome | 2937554 | 2937691 | + |
| chromsome | 2046368 | 2046505 | + |
| chromsome | 3019919 | 3020058 | - |
| chromsome | 729776 | 729916 | - |
| chromsome | 1930855 | 1930995 | - |
| chromsome | 2831690 | 2831830 | - |
| chromsome | 454495 | 454636 | - |
| chromsome | 3383849 | 3383990 | + |
| chromsome | 3274123 | 3274265 | + |
| chromsome | 3044591 | 3044734 | - |
| chromsome | 1806591 | 1806734 | + |
| chromsome | 3509757 | 3509901 | - |
| chromsome | 3028154 | 3028299 | + |
| chromsome | 953260 | 953405 | + |
| chromsome | 3417082 | 3417227 | + |
| chromsome | 2755398 | 2755544 | + |
| chromsome | 1190522 | 1190669 | + |
| chromsome | 2695383 | 2695531 | - |
| chromsome | 1311296 | 1311445 | + |
| chromsome | 508324 | 508475 | - |
| chromsome | 2938346 | 2938497 | + |
| chromsome | 2944 | 3095 | + |
| chromsome | 1599873 | 1600025 | - |
| chromsome | 1696827 | 1696979 | + |
| chromsome | 273453 | 273606 | - |
| chromsome | 1211321 | 1211475 | + |
| chromsome | 3449290 | 3449444 | + |
| chromsome | 1840801 | 1840956 | + |
| chromsome | 1700688 | 1700845 | - |
| chromsome | 1823753 | 1823910 | - |
| chromsome | 2215584 | 2215741 | + |
| chromsome | 1486345 | 1486502 | + |
| chromsome | 3095626 | 3095783 | + |
| chromsome | 3031896 | 3032053 | + |
| chromsome | 1728394 | 1728552 | - |
| chromsome | 450321 | 450480 | + |
| chromsome | 2950566 | 2950726 | + |
| chromsome | 1428001 | 1428162 | - |
| chromsome | 1486502 | 1486664 | - |
| chromsome | 2997466 | 2997628 | + |
| chromsome | 473190 | 473353 | - |
| chromsome | 1504566 | 1504729 | + |
| chromsome | 2707703 | 2707866 | + |
| chromsome | 1347105 | 1347268 | + |
| chromsome | 1840579 | 1840742 | + |
| chromsome | 668589 | 668753 | + |
| chromsome | 3526779 | 3526944 | - |
| chromsome | 2446186 | 2446352 | - |
| chromsome | 969578 | 969744 | + |
| chromsome | 734454 | 734621 | - |
| chromsome | 450488 | 450655 | - |
| chromsome | 3027976 | 3028143 | - |
| chromsome | 3210480 | 3210648 | + |
| chromsome | 1599843 | 1600013 | + |
| chromsome | 3165805 | 3165976 | - |
| chromsome | 700949 | 701120 | + |
| chromsome | 903711 | 903882 | + |
| chromsome | 1876990 | 1877162 | - |
| chromsome | 2067075 | 2067250 | + |
| chromsome | 2080645 | 2080824 | - |
| chromsome | 1213796 | 1213977 | - |
| chromsome | 1722985 | 1723166 | + |
| chromsome | 1881561 | 1881742 | + |
| chromsome | 2384977 | 2385159 | - |
| chromsome | 1337081 | 1337264 | - |
| chromsome | 1816636 | 1816820 | + |
| chromsome | 1148247 | 1148432 | - |
| chromsome | 1884937 | 1885122 | - |
| chromsome | 1524134 | 1524319 | + |
| chromsome | 658064 | 658250 | - |
| chromsome | 3465586 | 3465772 | - |
| chromsome | 2837585 | 2837771 | + |
| chromsome | 1579410 | 1579597 | - |
| chromsome | 3132163 | 3132352 | - |
| chromsome | 3353744 | 3353934 | - |
| chromsome | 2383688 | 2383878 | + |
| chromsome | 562072 | 562263 | - |
| chromsome | 704928 | 705119 | - |
| chromsome | 2706439 | 2706631 | - |
| chromsome | 347920 | 348112 | - |
| chromsome | 474546 | 474738 | - |
| chromsome | 3165633 | 3165825 | + |
| chromsome | 1806671 | 1806864 | - |
| chromsome | 1345845 | 1346038 | + |
| chromsome | 2473351 | 2473544 | + |
| chromsome | 3032088 | 3032282 | + |
| chromsome | 953414 | 953609 | + |
| chromsome | 206507 | 206703 | - |
| chromsome | 921236 | 921432 | + |
| chromsome | 2215934 | 2216131 | - |
| chromsome | 3427027 | 3427224 | - |
| chromsome | 1366823 | 1367021 | + |
| chromsome | 2707867 | 2708066 | + |
| chromsome | 1430175 | 1430377 | - |
| chromsome | 1112182 | 1112384 | - |
| chromsome | 3358992 | 3359194 | + |
| chromsome | 953294 | 953497 | - |
| chromsome | 2473437 | 2473640 | - |
| chromsome | 450232 | 450435 | - |
| chromsome | 2486253 | 2486457 | - |
| chromsome | 1562862 | 1563068 | - |
| chromsome | 2080303 | 2080509 | - |
| chromsome | 1075825 | 1076033 | - |
| chromsome | 1412664 | 1412872 | - |
| chromsome | 107233 | 107441 | + |
| chromsome | 450097 | 450305 | + |
| chromsome | 2817479 | 2817688 | + |
| chromsome | 612877 | 613087 | - |
| chromsome | 2779062 | 2779273 | + |
| chromsome | 624624 | 624838 | + |
| chromsome | 2553659 | 2553873 | + |
| chromsome | 3025962 | 3026180 | - |
| chromsome | 2461102 | 2461320 | + |
| chromsome | 1558731 | 1558949 | + |
| chromsome | 785246 | 785465 | - |
| chromsome | 1600032 | 1600251 | + |
| chromsome | 496101 | 496321 | + |
| chromsome | 1104709 | 1104929 | + |
| chromsome | 562040 | 562260 | + |
| chromsome | 610501 | 610722 | + |
| chromsome | 3027694 | 3027917 | - |
| chromsome | 1002298 | 1002522 | - |
| chromsome | 546 | 773 | - |
| chromsome | 914552 | 914780 | + |
| chromsome | 2290446 | 2290678 | - |
| chromsome | 1680014 | 1680247 | + |
| chromsome | 705037 | 705273 | + |
| chromsome | 3426949 | 3427185 | + |
| chromsome | 2311117 | 2311355 | - |
| chromsome | 2781035 | 2781274 | - |
| chromsome | 1049067 | 1049306 | + |
| chromsome | 3422726 | 3422966 | - |
| chromsome | 729738 | 729978 | + |
| chromsome | 727639 | 727880 | + |
| chromsome | 1215018 | 1215261 | + |
| chromsome | 2717772 | 2718016 | - |
| chromsome | 92809 | 93055 | - |
| chromsome | 3475677 | 3475923 | - |
| chromsome | 226571 | 226819 | + |
| chromsome | 2664049 | 2664297 | + |
| chromsome | 2755143 | 2755391 | + |
| chromsome | 900749 | 900998 | + |
| chromsome | 3006811 | 3007061 | - |
| chromsome | 605148 | 605403 | - |
| chromsome | 3039553 | 3039809 | + |
| chromsome | 2347178 | 2347436 | + |
| chromsome | 2121239 | 2121502 | - |
| chromsome | 1345881 | 1346144 | - |
| chromsome | 77973 | 78238 | + |
| chromsome | 3027842 | 3028107 | + |
| chromsome | 1981535 | 1981804 | - |
| chromsome | 298802 | 299076 | + |
| chromsome | 1639420 | 1639698 | + |
| chromsome | 3217488 | 3217769 | - |
| chromsome | 3474237 | 3474519 | + |
| chromsome | 1678977 | 1679262 | - |
| chromsome | 900772 | 901064 | - |
| chromsome | 347878 | 348171 | + |
| chromsome | 2676480 | 2676778 | - |
| chromsome | 1077311 | 1077610 | - |
| chromsome | 1553074 | 1553374 | - |
| chromsome | 3239774 | 3240078 | - |
| chromsome | 2706109 | 2706420 | - |
| chromsome | 2676615 | 2676927 | + |
| chromsome | 3359216 | 3359536 | + |
| chromsome | 1923850 | 1924175 | - |
| chromsome | 1467580 | 1467905 | + |
| chromsome | 1600126 | 1600459 | - |
| chromsome | 2646257 | 2646592 | + |
| chromsome | 2755292 | 2755633 | - |
| chromsome | 1483675 | 1484031 | + |
| chromsome | 2270195 | 2270556 | + |
| chromsome | 6633 | 6998 | - |
| chromsome | 1370695 | 1371060 | + |
| chromsome | 3359056 | 3359439 | - |
| chromsome | 3449355 | 3449758 | - |
| chromsome | 1679935 | 1680349 | - |
| chromsome | 2707756 | 2708178 | - |
| chromsome | 1881116 | 1881540 | - |
| chromsome | 1467537 | 1467971 | - |
| chromsome | 2553225 | 2553660 | - |
| chromsome | 2553205 | 2553657 | + |
| chromsome | 1880812 | 1881268 | + |
| chromsome | 3474536 | 3475016 | + |
| chromsome | 3572662 | 3573167 | + |
| chromsome | 106676 | 107206 | + |
| chromsome | 1606795 | 1607332 | - |
| chromsome | 3474321 | 3474890 | - |
| chromsome | 3044013 | 3044587 | - |
| chromsome | 3039225 | 3039833 | - |
| chromsome | 3044032 | 3044704 | + |
| pSYSM | 66503 | 66574 | + |
| pSYSM | 80389 | 80463 | + |
| pSYSM | 109966 | 110044 | - |
| pSYSM | 103354 | 103448 | - |
| pSYSM | 38274 | 38376 | - |
| pSYSM | 81926 | 82035 | + |
| pSYSM | 82095 | 82207 | + |
| pSYSM | 68794 | 68910 | + |
| pSYSM | 105395 | 105511 | + |
| pSYSM | 74335 | 74481 | + |
| pSYSM | 113516 | 113672 | + |
| pSYSM | 2022 | 2201 | + |
| pSYSM | 97175 | 97355 | - |
| pSYSM | 17570 | 17751 | + |
| pSYSM | 68845 | 69056 | - |
| pSYSM | 82000 | 82230 | - |
| pSYSM | 105470 | 105706 | - |
| pSYSM | 70878 | 71153 | + |
| pSYSM | 79006 | 79314 | + |
| pSYSM | 31006 | 31326 | - |
| pSYSM | 14036 | 14410 | - |
| pSYSA | 69916 | 69979 | - |
| pSYSA | 71090 | 71156 | + |
| pSYSA | 97028 | 97095 | - |
| pSYSA | 71164 | 71239 | + |
| pSYSA | 71187 | 71269 | - |
| pSYSA | 3859 | 3947 | - |
| pSYSA | 89939 | 90032 | - |
| pSYSA | 12576 | 12671 | - |
| pSYSA | 30096 | 30191 | - |
| pSYSA | 42770 | 42870 | + |
| pSYSA | 68231 | 68335 | - |
| pSYSA | 39282 | 39387 | - |
| pSYSA | 3 | 114 | + |
| pSYSA | 71938 | 72052 | - |
| pSYSA | 68774 | 68890 | - |
| pSYSA | 92262 | 92386 | - |
| pSYSA | 44610 | 44736 | - |
| pSYSA | 30014 | 30147 | + |
| pSYSA | 46522 | 46659 | - |
| pSYSA | 47246 | 47389 | - |
| pSYSA | 70188 | 70335 | + |
| pSYSA | 39274 | 39422 | + |
| pSYSA | 42799 | 42950 | - |
| pSYSA | 25702 | 25864 | - |
| pSYSA | 72155 | 72329 | - |
| pSYSA | 39759 | 39947 | + |
| pSYSA | 71687 | 71882 | + |
| pSYSA | 92419 | 92618 | - |
| pSYSA | 29875 | 30078 | - |
| pSYSA | 71933 | 72137 | + |
| pSYSA | 39075 | 39280 | - |
| pSYSA | 68560 | 68769 | - |
| pSYSA | 70462 | 70684 | - |
| pSYSA | 39446 | 39679 | + |
| pSYSA | 70880 | 71124 | - |
| pSYSA | 3813 | 4074 | + |
| pSYSA | 29732 | 29997 | + |
| pSYSA | 70068 | 70335 | - |
| pSYSA | 92804 | 93088 | - |
| pSYSA | 72299 | 72667 | + |
| pSYSA | 38896 | 39269 | + |
| pSYSA | 70337 | 70721 | + |
| pSYSA | 16239 | 17005 | - |
| pSYSA | 68081 | 68925 | + |
| pSYSA | 90128 | 91945 | - |
| pSYSG | 21558 | 21639 | + |
| pSYSG | 15392 | 15481 | + |
| pSYSG | 6 | 135 | - |
| pSYSG | 1739 | 1873 | + |
| pSYSG | 368 | 507 | - |
| pSYSG | 505 | 656 | + |
| pSYSG | 340 | 504 | + |
| pSYSG | 12039 | 12220 | + |
| pSYSG | 24884 | 25075 | - |
| pSYSG | 508 | 756 | - |
| pSYSG | 12125 | 12377 | - |
| pSYSG | 663 | 944 | + |
| pSYSG | 1 | 318 | + |
| pSYSG | 955 | 1394 | + |
| pSYSG | 779 | 1233 | - |
| pSYSX | 104459 | 104593 | + |
| pSYSX | 55455 | 55607 | - |
| pSYSX | 101832 | 102019 | - |
| pSYSX | 31871 | 32061 | - |
| pSYSX | 137 | 364 | - |
| pSYSX | 104539 | 104788 | - |
| pSYSX | 55326 | 55581 | + |

| **Table S2.** List of some putative top-scoring antisense RNAs(≥40 reads). | | |  |
| --- | --- | --- | --- |
| **Location** | **Start（nt）** | **End（nt）** | **Strand** |
| chromsome | 942308 | 942361 | - |
| chromsome | 2476561 | 2476615 | - |
| chromsome | 1391750 | 1391806 | - |
| chromsome | 2527435 | 2527493 | - |
| chromsome | 366267 | 366330 | - |
| chromsome | 1089931 | 1089998 | - |
| chromsome | 2177702 | 2177780 | - |
| chromsome | 2848054 | 2848144 | - |
| chromsome | 2844139 | 2844233 | - |
| chromsome | 279064 | 279161 | - |
| chromsome | 2101501 | 2101598 | - |
| chromsome | 2724266 | 2724365 | - |
| chromsome | 2211844 | 2211944 | - |
| chromsome | 2478723 | 2478824 | - |
| chromsome | 1061884 | 1061996 | - |
| chromsome | 3558868 | 3558980 | - |
| chromsome | 368537 | 368652 | - |
| chromsome | 3108265 | 3108382 | - |
| chromsome | 2214291 | 2214411 | - |
| chromsome | 67659 | 67782 | - |
| chromsome | 1207841 | 1207965 | - |
| chromsome | 3034538 | 3034662 | - |
| chromsome | 170992 | 171122 | - |
| chromsome | 611798 | 611932 | - |
| chromsome | 2925145 | 2925279 | - |
| chromsome | 2578850 | 2578985 | - |
| chromsome | 3009810 | 3009945 | - |
| chromsome | 3534017 | 3534156 | - |
| chromsome | 1911309 | 1911452 | - |
| chromsome | 2375763 | 2375906 | - |
| chromsome | 2455538 | 2455683 | - |
| chromsome | 431707 | 431852 | - |
| chromsome | 45848 | 45994 | - |
| chromsome | 385695 | 385841 | - |
| chromsome | 1078361 | 1078508 | - |
| chromsome | 229982 | 230130 | - |
| chromsome | 1326909 | 1327059 | - |
| chromsome | 81345 | 81495 | - |
| chromsome | 1547805 | 1547956 | - |
| chromsome | 67801 | 67954 | - |
| chromsome | 42645 | 42799 | - |
| chromsome | 610004 | 610160 | - |
| chromsome | 3038892 | 3039054 | - |
| chromsome | 46426 | 46590 | - |
| chromsome | 3519524 | 3519690 | - |
| chromsome | 729291 | 729457 | - |
| chromsome | 3046780 | 3046946 | - |
| chromsome | 2047003 | 2047172 | - |
| chromsome | 2340630 | 2340799 | - |
| chromsome | 2794005 | 2794174 | - |
| chromsome | 1367239 | 1367410 | - |
| chromsome | 3016245 | 3016417 | - |
| chromsome | 984770 | 984944 | - |
| chromsome | 3382038 | 3382212 | - |
| chromsome | 844419 | 844594 | - |
| chromsome | 139138 | 139315 | - |
| chromsome | 781668 | 781846 | - |
| chromsome | 1825619 | 1825800 | - |
| chromsome | 650418 | 650600 | - |
| chromsome | 2919013 | 2919195 | - |
| chromsome | 1328387 | 1328570 | - |
| chromsome | 2253402 | 2253585 | - |
| chromsome | 1908811 | 1908994 | - |
| chromsome | 2811403 | 2811588 | - |
| chromsome | 3336474 | 3336660 | - |
| chromsome | 3020608 | 3020794 | - |
| chromsome | 2656667 | 2656854 | - |
| chromsome | 3047901 | 3048088 | - |
| chromsome | 2500382 | 2500569 | - |
| chromsome | 1768455 | 1768643 | - |
| chromsome | 2752462 | 2752650 | - |
| chromsome | 3458864 | 3459052 | - |
| chromsome | 1019866 | 1020056 | - |
| chromsome | 696669 | 696861 | - |
| chromsome | 2867643 | 2867837 | - |
| chromsome | 1651560 | 1651755 | - |
| chromsome | 782258 | 782453 | - |
| chromsome | 2568475 | 2568672 | - |
| chromsome | 1639015 | 1639214 | - |
| chromsome | 2764675 | 2764875 | - |
| chromsome | 2219904 | 2220106 | - |
| chromsome | 278859 | 279062 | - |
| chromsome | 2815180 | 2815384 | - |
| chromsome | 2860674 | 2860878 | - |
| chromsome | 1338018 | 1338222 | - |
| chromsome | 2206931 | 2207136 | - |
| chromsome | 1745470 | 1745677 | - |
| chromsome | 3035100 | 3035312 | - |
| chromsome | 3001093 | 3001307 | - |
| chromsome | 1594621 | 1594835 | - |
| chromsome | 2799949 | 2800166 | - |
| chromsome | 1355777 | 1355995 | - |
| chromsome | 1539200 | 1539418 | - |
| chromsome | 2550975 | 2551194 | - |
| chromsome | 612127 | 612346 | - |
| chromsome | 1020080 | 1020302 | - |
| chromsome | 2081997 | 2082219 | - |
| chromsome | 1712158 | 1712381 | - |
| chromsome | 621877 | 622100 | - |
| chromsome | 3387970 | 3388193 | - |
| chromsome | 183515 | 183739 | - |
| chromsome | 3012682 | 3012906 | - |
| chromsome | 773142 | 773366 | - |
| chromsome | 2012341 | 2012566 | - |
| chromsome | 1547520 | 1547746 | - |
| chromsome | 1909216 | 1909443 | - |
| chromsome | 510019 | 510246 | - |
| chromsome | 984946 | 985174 | - |
| chromsome | 1573183 | 1573413 | - |
| chromsome | 772175 | 772405 | - |
| chromsome | 182948 | 183179 | - |
| chromsome | 961662 | 961893 | - |
| chromsome | 3551996 | 3552228 | - |
| chromsome | 2521383 | 2521617 | - |
| chromsome | 2204330 | 2204564 | - |
| chromsome | 1354094 | 1354328 | - |
| chromsome | 3566378 | 3566613 | - |
| chromsome | 3315087 | 3315323 | - |
| chromsome | 1636457 | 1636693 | - |
| chromsome | 1778377 | 1778614 | - |
| chromsome | 2901241 | 2901478 | - |
| chromsome | 3045156 | 3045394 | - |
| chromsome | 301388 | 301626 | - |
| chromsome | 1926686 | 1926925 | - |
| chromsome | 2745292 | 2745532 | - |
| chromsome | 1829177 | 1829418 | - |
| chromsome | 1340539 | 1340780 | - |
| chromsome | 1548087 | 1548329 | - |
| chromsome | 155785 | 156027 | - |
| chromsome | 1715825 | 1716068 | - |
| chromsome | 2806336 | 2806579 | - |
| chromsome | 1825806 | 1826049 | - |
| chromsome | 656785 | 657030 | - |
| chromsome | 962296 | 962541 | - |
| chromsome | 2776839 | 2777084 | - |
| chromsome | 3194093 | 3194339 | - |
| chromsome | 629434 | 629681 | - |
| chromsome | 3053280 | 3053527 | - |
| chromsome | 728694 | 728942 | - |
| chromsome | 739719 | 739967 | - |
| chromsome | 2847796 | 2848044 | - |
| chromsome | 3472646 | 3472895 | - |
| chromsome | 2829980 | 2830231 | - |
| chromsome | 45999 | 46251 | - |
| chromsome | 3198735 | 3198988 | - |
| chromsome | 438222 | 438475 | - |
| chromsome | 2230608 | 2230862 | - |
| chromsome | 2859240 | 2859496 | - |
| chromsome | 2129050 | 2129306 | - |
| chromsome | 1328117 | 1328374 | - |
| chromsome | 758301 | 758559 | - |
| chromsome | 3323830 | 3324088 | - |
| chromsome | 2161859 | 2162117 | - |
| chromsome | 2829252 | 2829510 | - |
| chromsome | 438513 | 438773 | - |
| chromsome | 680759 | 681019 | - |
| chromsome | 2919222 | 2919482 | - |
| chromsome | 384448 | 384708 | - |
| chromsome | 784925 | 785186 | - |
| chromsome | 558020 | 558283 | - |
| chromsome | 1229475 | 1229739 | - |
| chromsome | 2010328 | 2010593 | - |
| chromsome | 1928920 | 1929186 | - |
| chromsome | 272108 | 272376 | - |
| chromsome | 782647 | 782916 | - |
| chromsome | 2292245 | 2292516 | - |
| chromsome | 310415 | 310687 | - |
| chromsome | 2577062 | 2577334 | - |
| chromsome | 469759 | 470032 | - |
| chromsome | 653234 | 653507 | - |
| chromsome | 1865082 | 1865356 | - |
| chromsome | 1016038 | 1016313 | - |
| chromsome | 1582494 | 1582769 | - |
| chromsome | 2261633 | 2261911 | - |
| chromsome | 660125 | 660403 | - |
| chromsome | 2457898 | 2458176 | - |
| chromsome | 540152 | 540431 | - |
| chromsome | 154197 | 154478 | - |
| chromsome | 3337285 | 3337567 | - |
| chromsome | 1454330 | 1454612 | - |
| chromsome | 471140 | 471425 | - |
| chromsome | 651271 | 651557 | - |
| chromsome | 876947 | 877233 | - |
| chromsome | 2881985 | 2882271 | - |
| chromsome | 1909448 | 1909734 | - |
| chromsome | 3316113 | 3316402 | - |
| chromsome | 628205 | 628497 | - |
| chromsome | 907450 | 907743 | - |
| chromsome | 249385 | 249679 | - |
| chromsome | 2643715 | 2644010 | - |
| chromsome | 732769 | 733065 | - |
| chromsome | 2212861 | 2213158 | - |
| chromsome | 1647101 | 1647398 | - |
| chromsome | 628549 | 628846 | - |
| chromsome | 1047633 | 1047930 | - |
| chromsome | 155487 | 155784 | - |
| chromsome | 60735 | 61033 | - |
| chromsome | 759349 | 759647 | - |
| chromsome | 2498474 | 2498772 | - |
| chromsome | 902551 | 902850 | - |
| chromsome | 1746707 | 1747007 | - |
| chromsome | 3501625 | 3501925 | - |
| chromsome | 1525321 | 1525621 | - |
| chromsome | 1446122 | 1446423 | - |
| chromsome | 153178 | 153481 | - |
| chromsome | 2782534 | 2782838 | - |
| chromsome | 381275 | 381580 | - |
| chromsome | 1213010 | 1213316 | - |
| chromsome | 1446530 | 1446837 | - |
| chromsome | 3382815 | 3383123 | - |
| chromsome | 421271 | 421582 | - |
| chromsome | 3039884 | 3040196 | - |
| chromsome | 627575 | 627888 | - |
| chromsome | 3314696 | 3315011 | - |
| chromsome | 3362283 | 3362599 | - |
| chromsome | 278531 | 278847 | - |
| chromsome | 1647414 | 1647730 | - |
| chromsome | 2811080 | 2811399 | - |
| chromsome | 2601910 | 2602229 | - |
| chromsome | 67973 | 68293 | - |
| chromsome | 272911 | 273231 | - |
| chromsome | 2792229 | 2792551 | - |
| chromsome | 2027752 | 2028074 | - |
| chromsome | 2203239 | 2203561 | - |
| chromsome | 3523982 | 3524306 | - |
| chromsome | 3312959 | 3313283 | - |
| chromsome | 513314 | 513641 | - |
| chromsome | 1653510 | 1653837 | - |
| chromsome | 3348802 | 3349130 | - |
| chromsome | 300423 | 300752 | - |
| chromsome | 2261914 | 2262243 | - |
| chromsome | 728952 | 729282 | - |
| chromsome | 3476944 | 3477275 | - |
| chromsome | 1037599 | 1037930 | - |
| chromsome | 272548 | 272881 | - |
| chromsome | 3046068 | 3046401 | - |
| chromsome | 811516 | 811850 | - |
| chromsome | 1987896 | 1988230 | - |
| chromsome | 200677 | 201011 | - |
| chromsome | 1016344 | 1016682 | - |
| chromsome | 2543085 | 2543423 | - |
| chromsome | 1240553 | 1240892 | - |
| chromsome | 3496555 | 3496896 | - |
| chromsome | 3350929 | 3351271 | - |
| chromsome | 3033036 | 3033379 | - |
| chromsome | 657081 | 657424 | - |
| chromsome | 2283290 | 2283636 | - |
| chromsome | 3331861 | 3332208 | - |
| chromsome | 3362603 | 3362956 | - |
| chromsome | 45490 | 45844 | - |
| chromsome | 2491963 | 2492317 | - |
| chromsome | 418123 | 418478 | - |
| chromsome | 283064 | 283420 | - |
| chromsome | 1552461 | 1552821 | - |
| chromsome | 1510240 | 1510602 | - |
| chromsome | 1299211 | 1299574 | - |
| chromsome | 2800186 | 2800549 | - |
| chromsome | 1540817 | 1541182 | - |
| chromsome | 3197046 | 3197411 | - |
| chromsome | 994379 | 994745 | - |
| chromsome | 3565249 | 3565616 | - |
| chromsome | 1526575 | 1526945 | - |
| chromsome | 707651 | 708022 | - |
| chromsome | 2241875 | 2242247 | - |
| chromsome | 771767 | 772141 | - |
| chromsome | 597818 | 598192 | - |
| chromsome | 3408742 | 3409117 | - |
| chromsome | 415814 | 416190 | - |
| chromsome | 703089 | 703465 | - |
| chromsome | 3470331 | 3470711 | - |
| chromsome | 3114667 | 3115047 | - |
| chromsome | 1637028 | 1637410 | - |
| chromsome | 2231791 | 2232174 | - |
| chromsome | 120877 | 121260 | - |
| chromsome | 1236995 | 1237379 | - |
| chromsome | 419030 | 419414 | - |
| chromsome | 3261417 | 3261803 | - |
| chromsome | 781853 | 782240 | - |
| chromsome | 2343091 | 2343479 | - |
| chromsome | 40513 | 40901 | - |
| chromsome | 1330143 | 1330531 | - |
| chromsome | 1017104 | 1017493 | - |
| chromsome | 2329547 | 2329939 | - |
| chromsome | 985240 | 985632 | - |
| chromsome | 3256908 | 3257300 | - |
| chromsome | 2727494 | 2727888 | - |
| chromsome | 1244799 | 1245194 | - |
| chromsome | 116005 | 116401 | - |
| chromsome | 652675 | 653071 | - |
| chromsome | 1784341 | 1784737 | - |
| chromsome | 2249045 | 2249442 | - |
| chromsome | 2211946 | 2212344 | - |
| chromsome | 2213189 | 2213588 | - |
| chromsome | 1365815 | 1366215 | - |
| chromsome | 1323859 | 1324262 | - |
| chromsome | 2175505 | 2175908 | - |
| chromsome | 50795 | 51202 | - |
| chromsome | 1858972 | 1859379 | - |
| chromsome | 753594 | 754003 | - |
| chromsome | 2783552 | 2783962 | - |
| chromsome | 2237338 | 2237755 | - |
| chromsome | 510915 | 511332 | - |
| chromsome | 1781264 | 1781684 | - |
| chromsome | 3291437 | 3291858 | - |
| chromsome | 1262834 | 1263260 | - |
| chromsome | 815717 | 816144 | - |
| chromsome | 2211412 | 2211842 | - |
| chromsome | 3343478 | 3343910 | - |
| chromsome | 3337608 | 3338042 | - |
| chromsome | 2904017 | 2904451 | - |
| chromsome | 3202035 | 3202470 | - |
| chromsome | 2196515 | 2196950 | - |
| chromsome | 85593 | 86029 | - |
| chromsome | 2893745 | 2894183 | - |
| chromsome | 1385272 | 1385712 | - |
| chromsome | 2995914 | 2996355 | - |
| chromsome | 2281756 | 2282201 | - |
| chromsome | 1171238 | 1171685 | - |
| chromsome | 3453811 | 3454258 | - |
| chromsome | 1177518 | 1177965 | - |
| chromsome | 958598 | 959047 | - |
| chromsome | 3330448 | 3330898 | - |
| chromsome | 2810565 | 2811017 | - |
| chromsome | 276998 | 277451 | - |
| chromsome | 202045 | 202499 | - |
| chromsome | 2412178 | 2412635 | - |
| chromsome | 2947401 | 2947860 | - |
| chromsome | 2182461 | 2182924 | - |
| chromsome | 3043079 | 3043543 | - |
| chromsome | 2904686 | 2905154 | - |
| chromsome | 3549009 | 3549481 | - |
| chromsome | 256756 | 257230 | - |
| chromsome | 3217917 | 3218394 | - |
| chromsome | 3336715 | 3337198 | - |
| chromsome | 3516609 | 3517096 | - |
| chromsome | 723296 | 723785 | - |
| chromsome | 1251949 | 1252444 | - |
| chromsome | 1573873 | 1574369 | - |
| chromsome | 1543154 | 1543651 | - |
| chromsome | 928112 | 928609 | - |
| chromsome | 1327585 | 1328089 | - |
| chromsome | 953845 | 954353 | - |
| chromsome | 2766702 | 2767212 | - |
| chromsome | 1332708 | 1333220 | - |
| chromsome | 360505 | 361020 | - |
| chromsome | 2499504 | 2500025 | - |
| chromsome | 2170798 | 2171319 | - |
| chromsome | 2150763 | 2151292 | - |
| chromsome | 3036510 | 3037042 | - |
| chromsome | 2213757 | 2214290 | - |
| chromsome | 2857909 | 2858442 | - |
| chromsome | 1681547 | 1682084 | - |
| chromsome | 283691 | 284228 | - |
| chromsome | 3568093 | 3568633 | - |
| chromsome | 198812 | 199356 | - |
| chromsome | 1992180 | 1992726 | - |
| chromsome | 3566846 | 3567403 | - |
| chromsome | 1779043 | 1779616 | - |
| chromsome | 41159 | 41741 | - |
| chromsome | 1549289 | 1549878 | - |
| chromsome | 2204573 | 2205167 | - |
| chromsome | 3197810 | 3198406 | - |
| chromsome | 3033933 | 3034532 | - |
| chromsome | 1723843 | 1724443 | - |
| chromsome | 647635 | 648245 | - |
| chromsome | 3333678 | 3334293 | - |
| chromsome | 654416 | 655040 | - |
| chromsome | 1926969 | 1927603 | - |
| chromsome | 1869905 | 1870539 | - |
| chromsome | 653511 | 654173 | - |
| chromsome | 3335480 | 3336147 | - |
| chromsome | 3565640 | 3566311 | - |
| chromsome | 651835 | 652521 | - |
| chromsome | 655482 | 656206 | - |
| chromsome | 277465 | 278237 | - |
| chromsome | 2081027 | 2081814 | - |
| chromsome | 1551126 | 1551959 | - |
| chromsome | 699818 | 700715 | - |
| chromsome | 1333222 | 1334128 | - |
| chromsome | 3343923 | 3344901 | - |
| chromsome | 648500 | 649624 | - |
| chromsome | 1868677 | 1869904 | - |
| chromsome | 2581732 | 2581805 | + |
| chromsome | 2315611 | 2315685 | + |
| chromsome | 669425 | 669520 | + |
| chromsome | 1003653 | 1003752 | + |
| chromsome | 2650624 | 2650727 | + |
| chromsome | 738377 | 738483 | + |
| chromsome | 218847 | 218967 | + |
| chromsome | 720497 | 720617 | + |
| chromsome | 407802 | 407923 | + |
| chromsome | 2911702 | 2911824 | + |
| chromsome | 694964 | 695093 | + |
| chromsome | 2218450 | 2218584 | + |
| chromsome | 119117 | 119255 | + |
| chromsome | 1691601 | 1691740 | + |
| chromsome | 2595539 | 2595678 | + |
| chromsome | 285264 | 285403 | + |
| chromsome | 2058530 | 2058672 | + |
| chromsome | 2911331 | 2911474 | + |
| chromsome | 3099124 | 3099269 | + |
| chromsome | 346136 | 346282 | + |
| chromsome | 2541866 | 2542012 | + |
| chromsome | 1618239 | 1618388 | + |
| chromsome | 726651 | 726802 | + |
| chromsome | 345095 | 345246 | + |
| chromsome | 2714394 | 2714545 | + |
| chromsome | 1400635 | 1400787 | + |
| chromsome | 2405742 | 2405894 | + |
| chromsome | 553850 | 554003 | + |
| chromsome | 3514912 | 3515066 | + |
| chromsome | 2927593 | 2927747 | + |
| chromsome | 564280 | 564437 | + |
| chromsome | 865128 | 865288 | + |
| chromsome | 726486 | 726646 | + |
| chromsome | 3364286 | 3364447 | + |
| chromsome | 433899 | 434066 | + |
| chromsome | 1653126 | 1653294 | + |
| chromsome | 843798 | 843967 | + |
| chromsome | 3480881 | 3481050 | + |
| chromsome | 936988 | 937159 | + |
| chromsome | 839010 | 839182 | + |
| chromsome | 1857004 | 1857178 | + |
| chromsome | 2906030 | 2906204 | + |
| chromsome | 1423945 | 1424121 | + |
| chromsome | 1946331 | 1946508 | + |
| chromsome | 2937151 | 2937329 | + |
| chromsome | 1941248 | 1941429 | + |
| chromsome | 1062451 | 1062633 | + |
| chromsome | 1163576 | 1163759 | + |
| chromsome | 2033087 | 2033272 | + |
| chromsome | 3006417 | 3006602 | + |
| chromsome | 1306872 | 1307058 | + |
| chromsome | 1168726 | 1168912 | + |
| chromsome | 2861275 | 2861461 | + |
| chromsome | 939271 | 939459 | + |
| chromsome | 1518020 | 1518212 | + |
| chromsome | 1299851 | 1300043 | + |
| chromsome | 2708721 | 2708913 | + |
| chromsome | 152601 | 152794 | + |
| chromsome | 2795002 | 2795196 | + |
| chromsome | 2538067 | 2538262 | + |
| chromsome | 2200211 | 2200410 | + |
| chromsome | 1428697 | 1428898 | + |
| chromsome | 395799 | 396000 | + |
| chromsome | 1253758 | 1253960 | + |
| chromsome | 2314920 | 2315122 | + |
| chromsome | 1765928 | 1766131 | + |
| chromsome | 2472038 | 2472241 | + |
| chromsome | 1504215 | 1504420 | + |
| chromsome | 2474430 | 2474636 | + |
| chromsome | 2051724 | 2051931 | + |
| chromsome | 161892 | 162100 | + |
| chromsome | 3521088 | 3521299 | + |
| chromsome | 2736120 | 2736332 | + |
| chromsome | 3383628 | 3383843 | + |
| chromsome | 2937947 | 2938163 | + |
| chromsome | 27198 | 27415 | + |
| chromsome | 1544893 | 1545110 | + |
| chromsome | 31949 | 32166 | + |
| chromsome | 1665302 | 1665520 | + |
| chromsome | 1660748 | 1660967 | + |
| chromsome | 469091 | 469311 | + |
| chromsome | 635546 | 635766 | + |
| chromsome | 2057818 | 2058039 | + |
| chromsome | 940674 | 940896 | + |
| chromsome | 1796381 | 1796603 | + |
| chromsome | 2152306 | 2152529 | + |
| chromsome | 1662763 | 1662987 | + |
| chromsome | 2680559 | 2680785 | + |
| chromsome | 1642578 | 1642805 | + |
| chromsome | 1768783 | 1769014 | + |
| chromsome | 280300 | 280531 | + |
| chromsome | 100896 | 101129 | + |
| chromsome | 3363446 | 3363680 | + |
| chromsome | 670380 | 670614 | + |
| chromsome | 1359744 | 1359979 | + |
| chromsome | 3358196 | 3358431 | + |
| chromsome | 439576 | 439815 | + |
| chromsome | 829730 | 829969 | + |
| chromsome | 2114092 | 2114331 | + |
| chromsome | 1889122 | 1889362 | + |
| chromsome | 2918277 | 2918518 | + |
| chromsome | 3464755 | 3464996 | + |
| chromsome | 3049685 | 3049927 | + |
| chromsome | 2304728 | 2304970 | + |
| chromsome | 1939961 | 1940203 | + |
| chromsome | 634253 | 634496 | + |
| chromsome | 866080 | 866325 | + |
| chromsome | 2226007 | 2226253 | + |
| chromsome | 1896300 | 1896546 | + |
| chromsome | 987287 | 987534 | + |
| chromsome | 725103 | 725351 | + |
| chromsome | 2826285 | 2826533 | + |
| chromsome | 829145 | 829393 | + |
| chromsome | 2054767 | 2055015 | + |
| chromsome | 321142 | 321391 | + |
| chromsome | 3341522 | 3341772 | + |
| chromsome | 2571558 | 2571808 | + |
| chromsome | 1857239 | 1857490 | + |
| chromsome | 1024099 | 1024351 | + |
| chromsome | 1851929 | 1852183 | + |
| chromsome | 1220568 | 1220824 | + |
| chromsome | 3021902 | 3022159 | + |
| chromsome | 1158586 | 1158843 | + |
| chromsome | 3480593 | 3480851 | + |
| chromsome | 317327 | 317585 | + |
| chromsome | 939632 | 939891 | + |
| chromsome | 31680 | 31940 | + |
| chromsome | 2907639 | 2907899 | + |
| chromsome | 1065677 | 1065938 | + |
| chromsome | 1856653 | 1856915 | + |
| chromsome | 1480615 | 1480877 | + |
| chromsome | 695544 | 695807 | + |
| chromsome | 433367 | 433632 | + |
| chromsome | 1588649 | 1588915 | + |
| chromsome | 1891368 | 1891634 | + |
| chromsome | 31242 | 31508 | + |
| chromsome | 1664012 | 1664278 | + |
| chromsome | 928852 | 929123 | + |
| chromsome | 1115579 | 1115851 | + |
| chromsome | 1025064 | 1025336 | + |
| chromsome | 1231229 | 1231505 | + |
| chromsome | 2092687 | 2092965 | + |
| chromsome | 3289654 | 3289932 | + |
| chromsome | 3206696 | 3206974 | + |
| chromsome | 2940573 | 2940852 | + |
| chromsome | 2625632 | 2625913 | + |
| chromsome | 73517 | 73801 | + |
| chromsome | 26911 | 27196 | + |
| chromsome | 560342 | 560629 | + |
| chromsome | 399574 | 399864 | + |
| chromsome | 3475321 | 3475612 | + |
| chromsome | 3439157 | 3439452 | + |
| chromsome | 3407157 | 3407452 | + |
| chromsome | 563962 | 564258 | + |
| chromsome | 631150 | 631446 | + |
| chromsome | 507066 | 507363 | + |
| chromsome | 1665741 | 1666038 | + |
| chromsome | 1357756 | 1358053 | + |
| chromsome | 413764 | 414061 | + |
| chromsome | 938969 | 939267 | + |
| chromsome | 1131227 | 1131527 | + |
| chromsome | 392292 | 392592 | + |
| chromsome | 3100971 | 3101271 | + |
| chromsome | 3556571 | 3556871 | + |
| chromsome | 2086588 | 2086889 | + |
| chromsome | 163016 | 163317 | + |
| chromsome | 2314251 | 2314553 | + |
| chromsome | 819545 | 819849 | + |
| chromsome | 1283318 | 1283623 | + |
| chromsome | 33228 | 33533 | + |
| chromsome | 1297726 | 1298034 | + |
| chromsome | 997149 | 997458 | + |
| chromsome | 841407 | 841717 | + |
| chromsome | 2218071 | 2218386 | + |
| chromsome | 1792079 | 1792394 | + |
| chromsome | 2415539 | 2415855 | + |
| chromsome | 407926 | 408242 | + |
| chromsome | 1113117 | 1113435 | + |
| chromsome | 3028881 | 3029202 | + |
| chromsome | 2958757 | 2959081 | + |
| chromsome | 394800 | 395126 | + |
| chromsome | 2808260 | 2808586 | + |
| chromsome | 1905227 | 1905554 | + |
| chromsome | 683367 | 683696 | + |
| chromsome | 1038278 | 1038607 | + |
| chromsome | 20841 | 21171 | + |
| chromsome | 2096872 | 2097203 | + |
| chromsome | 239926 | 240257 | + |
| chromsome | 1110150 | 1110481 | + |
| chromsome | 2924201 | 2924532 | + |
| chromsome | 3406423 | 3406756 | + |
| chromsome | 1457875 | 1458208 | + |
| chromsome | 2229797 | 2230131 | + |
| chromsome | 2033326 | 2033661 | + |
| chromsome | 314493 | 314828 | + |
| chromsome | 617398 | 617736 | + |
| chromsome | 2538263 | 2538601 | + |
| chromsome | 1252858 | 1253197 | + |
| chromsome | 3346316 | 3346656 | + |
| chromsome | 1357154 | 1357495 | + |
| chromsome | 634523 | 634864 | + |
| chromsome | 3321060 | 3321401 | + |
| chromsome | 2667676 | 2668020 | + |
| chromsome | 3077247 | 3077591 | + |
| chromsome | 938580 | 938926 | + |
| chromsome | 865710 | 866057 | + |
| chromsome | 2605054 | 2605402 | + |
| chromsome | 2321957 | 2322306 | + |
| chromsome | 1122188 | 1122538 | + |
| chromsome | 3446180 | 3446530 | + |
| chromsome | 2916212 | 2916563 | + |
| chromsome | 3028511 | 3028863 | + |
| chromsome | 2606410 | 2606763 | + |
| chromsome | 2740234 | 2740587 | + |
| chromsome | 635844 | 636197 | + |
| chromsome | 1344421 | 1344775 | + |
| chromsome | 2826760 | 2827116 | + |
| chromsome | 1082890 | 1083246 | + |
| chromsome | 2532034 | 2532391 | + |
| chromsome | 2939144 | 2939503 | + |
| chromsome | 866472 | 866833 | + |
| chromsome | 2332825 | 2333186 | + |
| chromsome | 3439573 | 3439934 | + |
| chromsome | 2512538 | 2512900 | + |
| chromsome | 405735 | 406097 | + |
| chromsome | 954729 | 955093 | + |
| chromsome | 1082507 | 1082875 | + |
| chromsome | 1978434 | 1978804 | + |
| chromsome | 2331533 | 2331903 | + |
| chromsome | 3773 | 4144 | + |
| chromsome | 2878219 | 2878592 | + |
| chromsome | 2335129 | 2335502 | + |
| chromsome | 3478389 | 3478762 | + |
| chromsome | 857608 | 857983 | + |
| chromsome | 407408 | 407784 | + |
| chromsome | 3515581 | 3515957 | + |
| chromsome | 82578 | 82963 | + |
| chromsome | 2217683 | 2218070 | + |
| chromsome | 3356895 | 3357284 | + |
| chromsome | 26055 | 26444 | + |
| chromsome | 865304 | 865694 | + |
| chromsome | 2641337 | 2641728 | + |
| chromsome | 947590 | 947985 | + |
| chromsome | 2877034 | 2877432 | + |
| chromsome | 1460886 | 1461285 | + |
| chromsome | 1852511 | 1852910 | + |
| chromsome | 2503448 | 2503847 | + |
| chromsome | 130634 | 131035 | + |
| chromsome | 477797 | 478200 | + |
| chromsome | 3520637 | 3521041 | + |
| chromsome | 724534 | 724938 | + |
| chromsome | 2742564 | 2742968 | + |
| chromsome | 47511 | 47916 | + |
| chromsome | 1641591 | 1641998 | + |
| chromsome | 2585556 | 2585964 | + |
| chromsome | 3356044 | 3356454 | + |
| chromsome | 2102146 | 2102558 | + |
| chromsome | 267937 | 268349 | + |
| chromsome | 2560764 | 2561178 | + |
| chromsome | 408291 | 408705 | + |
| chromsome | 2559244 | 2559663 | + |
| chromsome | 3522531 | 3522950 | + |
| chromsome | 718088 | 718509 | + |
| chromsome | 1782857 | 1783279 | + |
| chromsome | 2945666 | 2946089 | + |
| chromsome | 507568 | 507992 | + |
| chromsome | 1451414 | 1451838 | + |
| chromsome | 3440177 | 3440603 | + |
| chromsome | 167535 | 167963 | + |
| chromsome | 3050474 | 3050904 | + |
| chromsome | 1878301 | 1878731 | + |
| chromsome | 1841965 | 1842402 | + |
| chromsome | 863820 | 864259 | + |
| chromsome | 1660985 | 1661432 | + |
| chromsome | 3514445 | 3514895 | + |
| chromsome | 712207 | 712663 | + |
| chromsome | 2528276 | 2528732 | + |
| chromsome | 411037 | 411493 | + |
| chromsome | 1286930 | 1287386 | + |
| chromsome | 267048 | 267509 | + |
| chromsome | 342219 | 342680 | + |
| chromsome | 2825811 | 2826273 | + |
| chromsome | 1785010 | 1785473 | + |
| chromsome | 1508892 | 1509357 | + |
| chromsome | 2640859 | 2641324 | + |
| chromsome | 3354734 | 3355199 | + |
| chromsome | 2942381 | 2942849 | + |
| chromsome | 395196 | 395666 | + |
| chromsome | 2093029 | 2093507 | + |
| chromsome | 3057257 | 3057736 | + |
| chromsome | 1456054 | 1456541 | + |
| chromsome | 2229081 | 2229569 | + |
| chromsome | 1890879 | 1891367 | + |
| chromsome | 2093672 | 2094162 | + |
| chromsome | 405238 | 405729 | + |
| chromsome | 214415 | 214915 | + |
| chromsome | 1014598 | 1015102 | + |
| chromsome | 685883 | 686392 | + |
| chromsome | 1658517 | 1659027 | + |
| chromsome | 2349034 | 2349547 | + |
| chromsome | 711643 | 712161 | + |
| chromsome | 1887505 | 1888029 | + |
| chromsome | 1264556 | 1265081 | + |
| chromsome | 1527171 | 1527698 | + |
| chromsome | 2569848 | 2570377 | + |
| chromsome | 2943052 | 2943582 | + |
| chromsome | 116901 | 117432 | + |
| chromsome | 3363743 | 3364283 | + |
| chromsome | 2348438 | 2348981 | + |
| chromsome | 1543968 | 1544515 | + |
| chromsome | 1083932 | 1084491 | + |
| chromsome | 2697207 | 2697773 | + |
| chromsome | 1531650 | 1532225 | + |
| chromsome | 719906 | 720492 | + |
| chromsome | 96617 | 97204 | + |
| chromsome | 2332181 | 2332769 | + |
| chromsome | 1586259 | 1586868 | + |
| chromsome | 1008487 | 1009116 | + |
| chromsome | 389486 | 390120 | + |
| chromsome | 1276262 | 1276914 | + |
| chromsome | 1618658 | 1619319 | + |
| chromsome | 411815 | 412478 | + |
| chromsome | 166834 | 167501 | + |
| chromsome | 564473 | 565158 | + |
| chromsome | 346673 | 347386 | + |
| chromsome | 1583537 | 1584255 | + |
| chromsome | 3153453 | 3154181 | + |
| chromsome | 622701 | 623442 | + |
| chromsome | 2252199 | 2252941 | + |
| chromsome | 567663 | 568452 | + |
| chromsome | 434753 | 435636 | + |
| chromsome | 412479 | 413489 | + |
| chromsome | 406118 | 407229 | + |
| pSYSM | 58000 | 58146 | - |
| pSYSM | 5173 | 5373 | - |
| pSYSM | 26070 | 26339 | - |
| pSYSM | 3082 | 3363 | - |
| pSYSM | 58278 | 58571 | - |
| pSYSM | 5583 | 5909 | - |
| pSYSM | 3497 | 4094 | - |
| pSYSM | 60438 | 60541 | + |
| pSYSM | 48508 | 48660 | + |
| pSYSM | 65614 | 65774 | + |
| pSYSM | 45048 | 45251 | + |
| pSYSM | 47193 | 47410 | + |
| pSYSM | 20670 | 20891 | + |
| pSYSM | 71192 | 71427 | + |
| pSYSM | 115905 | 116156 | + |
| pSYSM | 28818 | 29069 | + |
| pSYSM | 74656 | 74973 | + |
| pSYSM | 116962 | 117279 | + |
| pSYSM | 35460 | 35780 | + |
| pSYSM | 77531 | 77854 | + |
| pSYSM | 61315 | 61640 | + |
| pSYSM | 60738 | 61072 | + |
| pSYSM | 110932 | 111271 | + |
| pSYSM | 86164 | 86511 | + |
| pSYSM | 48754 | 49102 | + |
| pSYSM | 57276 | 57627 | + |
| pSYSM | 83389 | 83794 | + |
| pSYSM | 40621 | 41139 | + |
| pSYSM | 43351 | 43921 | + |
| pSYSM | 32153 | 32731 | + |
| pSYSA | 89255 | 89404 | - |
| pSYSA | 78037 | 78223 | - |
| pSYSA | 83528 | 83734 | - |
| pSYSA | 23693 | 23906 | - |
| pSYSA | 6603 | 6820 | - |
| pSYSA | 11992 | 12211 | - |
| pSYSA | 36892 | 37113 | - |
| pSYSA | 21371 | 21627 | - |
| pSYSA | 33566 | 33839 | - |
| pSYSA | 14870 | 15177 | - |
| pSYSA | 20906 | 21258 | - |
| pSYSA | 34712 | 35065 | - |
| pSYSA | 65381 | 65743 | - |
| pSYSA | 33101 | 33524 | - |
| pSYSA | 33841 | 34293 | - |
| pSYSA | 63213 | 63750 | - |
| pSYSA | 51773 | 52322 | - |
| pSYSA | 97624 | 98197 | - |
| pSYSA | 43843 | 43996 | + |
| pSYSA | 84825 | 84997 | + |
| pSYSA | 41151 | 41343 | + |
| pSYSA | 53041 | 53312 | + |
| pSYSA | 59904 | 60186 | + |
| pSYSA | 54547 | 54829 | + |
| pSYSA | 61842 | 62177 | + |
| pSYSA | 101642 | 101982 | + |
| pSYSA | 67127 | 67482 | + |
| pSYSA | 38306 | 38686 | + |
| pSYSA | 40669 | 41074 | + |
| pSYSA | 84071 | 84477 | + |
| pSYSA | 67508 | 67941 | + |
| pSYSA | 73908 | 74410 | + |
| pSYSA | 57406 | 57933 | + |
| pSYSA | 59068 | 59618 | + |
| pSYSA | 5492 | 6164 | + |
| pSYSG | 25614 | 25797 | - |
| pSYSG | 40071 | 40266 | - |
| pSYSG | 26732 | 26936 | - |
| pSYSG | 26307 | 26615 | - |
| pSYSG | 39718 | 40052 | - |
| pSYSG | 20774 | 21112 | - |
| pSYSG | 20051 | 20418 | - |
| pSYSG | 34122 | 34582 | - |
| pSYSG | 34762 | 35339 | - |
| pSYSG | 4031 | 4277 | + |
| pSYSG | 2695 | 2951 | + |
| pSYSG | 15666 | 15932 | + |
| pSYSG | 42602 | 42880 | + |
| pSYSG | 37498 | 37807 | + |
| pSYSG | 15959 | 16356 | + |
| pSYSG | 2141 | 2609 | + |
| pSYSG | 43561 | 44086 | + |
| pSYSG | 4801 | 5461 | + |
| pSYSG | 16372 | 17134 | + |
| pSYSX | 96197 | 96338 | - |
| pSYSX | 48525 | 48677 | - |
| pSYSX | 91564 | 91738 | - |
| pSYSX | 50184 | 50361 | - |
| pSYSX | 50373 | 50558 | - |
| pSYSX | 45060 | 45251 | - |
| pSYSX | 28783 | 28997 | - |
| pSYSX | 28998 | 29212 | - |
| pSYSX | 41272 | 41499 | - |
| pSYSX | 49296 | 49530 | - |
| pSYSX | 54351 | 54608 | - |
| pSYSX | 39411 | 39672 | - |
| pSYSX | 84920 | 85187 | - |
| pSYSX | 49021 | 49292 | - |
| pSYSX | 37869 | 38157 | - |
| pSYSX | 41509 | 41801 | - |
| pSYSX | 467 | 787 | - |
| pSYSX | 83046 | 83371 | - |
| pSYSX | 31432 | 31764 | - |
| pSYSX | 85402 | 85765 | - |
| pSYSX | 46633 | 46997 | - |
| pSYSX | 96366 | 96807 | - |
| pSYSX | 83841 | 84328 | - |
| pSYSX | 44533 | 45050 | - |
| pSYSX | 45301 | 45825 | - |
| pSYSX | 43795 | 44374 | - |
| pSYSX | 45837 | 46422 | - |
| pSYSX | 49535 | 50162 | - |
| pSYSX | 40020 | 40783 | - |
| pSYSX | 56471 | 56707 | + |
| pSYSX | 52003 | 52282 | + |
| pSYSX | 52311 | 52782 | + |

| **Table S3.** List of all predicted 5’ leader region candidates. | |  |  |
| --- | --- | --- | --- |
| **Location** | **Start(nt)** | **End(nt)** | **Strand** |
| chromsome | 1650682 | 1650733 | - |
| chromsome | 516010 | 516063 | - |
| chromsome | 2909396 | 2909455 | - |
| chromsome | 2918872 | 2918933 | - |
| chromsome | 3322891 | 3322952 | - |
| chromsome | 99259 | 99326 | - |
| chromsome | 2227461 | 2227529 | - |
| chromsome | 1655762 | 1655831 | - |
| chromsome | 1770481 | 1770553 | - |
| chromsome | 3182189 | 3182267 | - |
| chromsome | 1289173 | 1289253 | - |
| chromsome | 3422642 | 3422723 | - |
| chromsome | 1236097 | 1236179 | - |
| chromsome | 2906517 | 2906600 | - |
| chromsome | 955470 | 955554 | - |
| chromsome | 3261311 | 3261401 | - |
| chromsome | 3479100 | 3479191 | - |
| chromsome | 3422501 | 3422593 | - |
| chromsome | 2333867 | 2333960 | - |
| chromsome | 2580144 | 2580238 | - |
| chromsome | 2193434 | 2193529 | - |
| chromsome | 1330014 | 1330109 | - |
| chromsome | 531333 | 531428 | - |
| chromsome | 1452600 | 1452696 | - |
| chromsome | 2812742 | 2812839 | - |
| chromsome | 255671 | 255769 | - |
| chromsome | 2735692 | 2735792 | - |
| chromsome | 321529 | 321632 | - |
| chromsome | 583575 | 583679 | - |
| chromsome | 2469045 | 2469149 | - |
| chromsome | 2193958 | 2194066 | - |
| chromsome | 2456795 | 2456906 | - |
| chromsome | 231884 | 231996 | - |
| chromsome | 801386 | 801498 | - |
| chromsome | 3358867 | 3358981 | - |
| chromsome | 2384858 | 2384972 | - |
| chromsome | 3211655 | 3211771 | - |
| chromsome | 1853873 | 1853992 | - |
| chromsome | 2528859 | 2528982 | - |
| chromsome | 618769 | 618892 | - |
| chromsome | 1488453 | 1488577 | - |
| chromsome | 106683 | 106809 | - |
| chromsome | 1555849 | 1555975 | - |
| chromsome | 2461079 | 2461212 | - |
| chromsome | 3493507 | 3493642 | - |
| chromsome | 2553087 | 2553223 | - |
| chromsome | 1320550 | 1320686 | - |
| chromsome | 3025806 | 3025943 | - |
| chromsome | 1388444 | 1388581 | - |
| chromsome | 3095728 | 3095866 | - |
| chromsome | 1682419 | 1682561 | - |
| chromsome | 2134050 | 2134192 | - |
| chromsome | 1678616 | 1678760 | - |
| chromsome | 3457000 | 3457144 | - |
| chromsome | 2817459 | 2817607 | - |
| chromsome | 1948925 | 1949076 | - |
| chromsome | 1130139 | 1130297 | - |
| chromsome | 1180772 | 1180933 | - |
| chromsome | 1077080 | 1077242 | - |
| chromsome | 1467361 | 1467527 | - |
| chromsome | 2146536 | 2146706 | - |
| chromsome | 2924588 | 2924764 | - |
| chromsome | 3295316 | 3295493 | - |
| chromsome | 1270777 | 1270954 | - |
| chromsome | 3449172 | 3449353 | - |
| chromsome | 2837602 | 2837785 | - |
| chromsome | 953105 | 953289 | - |
| chromsome | 1896623 | 1896815 | - |
| chromsome | 755640 | 755839 | - |
| chromsome | 1726345 | 1726546 | - |
| chromsome | 3074766 | 3074970 | - |
| chromsome | 456591 | 456795 | - |
| chromsome | 645619 | 645823 | - |
| chromsome | 1554796 | 1555007 | - |
| chromsome | 465651 | 465713 | + |
| chromsome | 3433172 | 3433235 | + |
| chromsome | 2890488 | 2890553 | + |
| chromsome | 2004492 | 2004561 | + |
| chromsome | 907867 | 907936 | + |
| chromsome | 2486446 | 2486516 | + |
| chromsome | 2302535 | 2302611 | + |
| chromsome | 3037148 | 3037224 | + |
| chromsome | 1397582 | 1397660 | + |
| chromsome | 2730512 | 2730591 | + |
| chromsome | 1991467 | 1991546 | + |
| chromsome | 658155 | 658235 | + |
| chromsome | 2796883 | 2796968 | + |
| chromsome | 609343 | 609432 | + |
| chromsome | 707230 | 707321 | + |
| chromsome | 3014253 | 3014345 | + |
| chromsome | 1853896 | 1853991 | + |
| chromsome | 482727 | 482824 | + |
| chromsome | 3311435 | 3311535 | + |
| chromsome | 168246 | 168348 | + |
| chromsome | 3032287 | 3032393 | + |
| chromsome | 920129 | 920236 | + |
| chromsome | 2847210 | 2847322 | + |
| chromsome | 1832226 | 1832339 | + |
| chromsome | 456832 | 456945 | + |
| chromsome | 1180818 | 1180931 | + |
| chromsome | 2414344 | 2414457 | + |
| chromsome | 482828 | 482942 | + |
| chromsome | 2890368 | 2890483 | + |
| chromsome | 2754655 | 2754773 | + |
| chromsome | 3384000 | 3384119 | + |
| chromsome | 2867241 | 2867360 | + |
| chromsome | 901005 | 901125 | + |
| chromsome | 594297 | 594417 | + |
| chromsome | 1981764 | 1981884 | + |
| chromsome | 429675 | 429796 | + |
| chromsome | 1077551 | 1077674 | + |
| chromsome | 1949406 | 1949529 | + |
| chromsome | 3347782 | 3347906 | + |
| chromsome | 1587059 | 1587184 | + |
| chromsome | 1874999 | 1875124 | + |
| chromsome | 2655242 | 2655369 | + |
| chromsome | 1942762 | 1942889 | + |
| chromsome | 2127664 | 2127791 | + |
| chromsome | 2230330 | 2230457 | + |
| chromsome | 1899525 | 1899653 | + |
| chromsome | 489452 | 489584 | + |
| chromsome | 1104088 | 1104228 | + |
| chromsome | 210579 | 210720 | + |
| chromsome | 1470354 | 1470495 | + |
| chromsome | 3342574 | 3342720 | + |
| chromsome | 3168882 | 3169030 | + |
| chromsome | 54361 | 54515 | + |
| chromsome | 1304788 | 1304949 | + |
| chromsome | 3239925 | 3240088 | + |
| chromsome | 3279149 | 3279315 | + |
| chromsome | 568916 | 569083 | + |
| chromsome | 2809820 | 2809990 | + |
| chromsome | 2677875 | 2678046 | + |
| chromsome | 1413917 | 1414091 | + |
| chromsome | 679055 | 679230 | + |
| chromsome | 1011763 | 1011939 | + |
| chromsome | 3273503 | 3273680 | + |
| chromsome | 2329040 | 2329217 | + |
| chromsome | 2227587 | 2227765 | + |
| chromsome | 2740673 | 2740864 | + |
| chromsome | 83869 | 84068 | + |
| chromsome | 1337019 | 1337257 | + |
| chromsome | 458917 | 459162 | + |
| chromsome | 1700626 | 1700871 | + |
| pSYSM | 2030 | 2091 | - |
| pSYSM | 84225 | 84306 | - |
| pSYSM | 102301 | 102387 | - |
| pSYSM | 74281 | 74431 | - |
| pSYSM | 80370 | 80562 | - |
| pSYSM | 31258 | 31364 | + |
| pSYSM | 31130 | 31257 | + |
| pSYSM | 80492 | 80776 | + |
| pSYSA | 31519 | 31622 | - |
| pSYSA | 46354 | 46491 | - |
| pSYSA | 81975 | 82131 | - |
| pSYSA | 93029 | 93131 | + |
| pSYSA | 31525 | 31639 | + |
| pSYSG | 21533 | 21745 | - |
| pSYSX | 57006 | 57075 | - |
| pSYSX | 56855 | 56966 | - |
| pSYSX | 58024 | 58147 | - |

| **Table S4.** Classification of predicted targets of candidate small regulatory RNAs according to their biological process, molecular function and cellular component. | | | |
| --- | --- | --- | --- |
| **ID** | **Gene Name** | **Terms** | **GO_ID** |
| **Biological Process** |  |  |  |
| slr2099 | Sensor protein | two-component signal transduction system (phosphorelay) | GO:0000160 |
| slr1969 | Sensor protein | two-component signal transduction system (phosphorelay) | GO:0000160 |
| slr6001 | Sensor protein | two-component signal transduction system (phosphorelay) | GO:0000160 |
| slr2104 | Sensor protein | two-component signal transduction system (phosphorelay) | GO:0000160 |
| slr1324 | Sensor protein | two-component signal transduction system (phosphorelay) | GO:0000160 |
| slr2098 | Sensor protein | two-component signal transduction system (phosphorelay) | GO:0000160 |
| sll1228 | Sensor protein | two-component signal transduction system (phosphorelay) | GO:0000160 |
| slr0222 | Sensor protein | two-component signal transduction system (phosphorelay) | GO:0000160 |
| sll0474 | Sensor protein | two-component signal transduction system (phosphorelay) | GO:0000160 |
| sll1229 | Sensor protein | two-component signal transduction system (phosphorelay) | GO:0000160 |
| sll1672 | Sensor protein | two-component signal transduction system (phosphorelay) | GO:0000160 |
| sll5060 | Sensor protein | two-component signal transduction system (phosphorelay) | GO:0000160 |
| slr1393 | Sensor protein | two-component signal transduction system (phosphorelay) | GO:0000160 |
| sll1124 | Sensor protein | two-component signal transduction system (phosphorelay) | GO:0000160 |
| sll1905 | Sensor protein | two-component signal transduction system (phosphorelay) | GO:0000160 |
| sll1879 | Ycf55-like protein | two-component signal transduction system (phosphorelay) | GO:0000160 |
| sll0038 | sll0038 | two-component signal transduction system (phosphorelay) | GO:0000160 |
| sll0267 | sll0267 | two-component signal transduction system (phosphorelay) | GO:0000160 |
| sll0779 | sll0779 | two-component signal transduction system (phosphorelay) | GO:0000160 |
| sll0782 | sll0782 | two-component signal transduction system (phosphorelay) | GO:0000160 |
| sll0797 | sll0797 | two-component signal transduction system (phosphorelay) | GO:0000160 |
| sll1296 | sll1296 | two-component signal transduction system (phosphorelay) | GO:0000160 |
| sll1330 | sll1330 | two-component signal transduction system (phosphorelay) | GO:0000160 |
| sll1592 | sll1592 | two-component signal transduction system (phosphorelay) | GO:0000160 |
| sll1687 | sll1687 | two-component signal transduction system (phosphorelay) | GO:0000160 |
| sll5059 | sll5059 | two-component signal transduction system (phosphorelay) | GO:0000160 |
| slr0115 | slr0115 | two-component signal transduction system (phosphorelay) | GO:0000160 |
| slr0302 | slr0302 | two-component signal transduction system (phosphorelay) | GO:0000160 |
| slr0311 | slr0311 | two-component signal transduction system (phosphorelay) | GO:0000160 |
| slr0312 | slr0312 | two-component signal transduction system (phosphorelay) | GO:0000160 |
| slr0359 | slr0359 | two-component signal transduction system (phosphorelay) | GO:0000160 |
| slr1041 | slr1041 | two-component signal transduction system (phosphorelay) | GO:0000160 |
| slr1214 | slr1214 | two-component signal transduction system (phosphorelay) | GO:0000160 |
| slr1305 | slr1305 | two-component signal transduction system (phosphorelay) | GO:0000160 |
| slr1588 | slr1588 | two-component signal transduction system (phosphorelay) | GO:0000160 |
| slr1594 | slr1594 | two-component signal transduction system (phosphorelay) | GO:0000160 |
| slr1693 | slr1693 | two-component signal transduction system (phosphorelay) | GO:0000160 |
| slr1837 | slr1837 | two-component signal transduction system (phosphorelay) | GO:0000160 |
| slr2100 | slr2100 | two-component signal transduction system (phosphorelay) | GO:0000160 |
| slr0756 | Circadian clock protein kaiA | protein amino acid phosphorylation | GO:0006468 |
| slr2099 | Sensor protein | protein amino acid phosphorylation | GO:0006468 |
| slr1285 | Sensor protein | protein amino acid phosphorylation | GO:0006468 |
| slr1969 | Sensor protein | protein amino acid phosphorylation | GO:0006468 |
| slr6001 | Sensor protein | protein amino acid phosphorylation | GO:0006468 |
| slr2104 | Sensor protein | protein amino acid phosphorylation | GO:0006468 |
| slr1324 | Sensor protein | protein amino acid phosphorylation | GO:0006468 |
| sll1888 | Sensor protein | protein amino acid phosphorylation | GO:0006468 |
| slr2098 | Sensor protein | protein amino acid phosphorylation | GO:0006468 |
| slr0640 | Sensor protein | protein amino acid phosphorylation | GO:0006468 |
| sll1228 | Sensor protein | protein amino acid phosphorylation | GO:0006468 |
| slr0222 | Sensor protein | protein amino acid phosphorylation | GO:0006468 |
| slr1147 | Sensor protein | protein amino acid phosphorylation | GO:0006468 |
| sll0474 | Sensor protein | protein amino acid phosphorylation | GO:0006468 |
| sll1672 | Sensor protein | protein amino acid phosphorylation | GO:0006468 |
| sll1229 | Sensor protein | protein amino acid phosphorylation | GO:0006468 |
| sll5060 | Sensor protein | protein amino acid phosphorylation | GO:0006468 |
| sll0798 | Sensor protein | protein amino acid phosphorylation | GO:0006468 |
| slr1393 | Sensor protein | protein amino acid phosphorylation | GO:0006468 |
| sll1124 | Sensor protein | protein amino acid phosphorylation | GO:0006468 |
| sll1905 | Sensor protein | protein amino acid phosphorylation | GO:0006468 |
| sll1575 | Serine/threonine-protein kinase A | protein amino acid phosphorylation | GO:0006468 |
| slr0599 | Serine/threonine-protein kinase C | protein amino acid phosphorylation | GO:0006468 |
| sll1296 | sll1296 | protein amino acid phosphorylation | GO:0006468 |
| slr0152 | slr0152 | protein amino acid phosphorylation | GO:0006468 |
| slr0311 | slr0311 | protein amino acid phosphorylation | GO:0006468 |
| sll0998 | Probable RuBisCO transcriptional regulator | regulation of transcription, DNA-dependent | GO:0006355 |
| slr2099 | Sensor protein | regulation of transcription, DNA-dependent | GO:0006355 |
| slr1969 | Sensor protein | regulation of transcription, DNA-dependent | GO:0006355 |
| slr6001 | Sensor protein | regulation of transcription, DNA-dependent | GO:0006355 |
| slr2104 | Sensor protein | regulation of transcription, DNA-dependent | GO:0006355 |
| slr1324 | Sensor protein | regulation of transcription, DNA-dependent | GO:0006355 |
| slr2098 | Sensor protein | regulation of transcription, DNA-dependent | GO:0006355 |
| sll1228 | Sensor protein | regulation of transcription, DNA-dependent | GO:0006355 |
| slr0222 | Sensor protein | regulation of transcription, DNA-dependent | GO:0006355 |
| sll0474 | Sensor protein | regulation of transcription, DNA-dependent | GO:0006355 |
| sll1672 | Sensor protein | regulation of transcription, DNA-dependent | GO:0006355 |
| sll1229 | Sensor protein | regulation of transcription, DNA-dependent | GO:0006355 |
| sll5060 | Sensor protein | regulation of transcription, DNA-dependent | GO:0006355 |
| slr1393 | Sensor protein | regulation of transcription, DNA-dependent | GO:0006355 |
| sll1124 | Sensor protein | regulation of transcription, DNA-dependent | GO:0006355 |
| sll1905 | Sensor protein | regulation of transcription, DNA-dependent | GO:0006355 |
| sll1879 | Ycf55-like protein | regulation of transcription, DNA-dependent | GO:0006355 |
| sll0038 | sll0038 | regulation of transcription, DNA-dependent | GO:0006355 |
| sll0267 | sll0267 | regulation of transcription, DNA-dependent | GO:0006355 |
| sll0779 | sll0779 | regulation of transcription, DNA-dependent | GO:0006355 |
| sll0782 | sll0782 | regulation of transcription, DNA-dependent | GO:0006355 |
| sll0797 | sll0797 | regulation of transcription, DNA-dependent | GO:0006355 |
| sll1296 | sll1296 | regulation of transcription, DNA-dependent | GO:0006355 |
| sll1330 | sll1330 | regulation of transcription, DNA-dependent | GO:0006355 |
| sll1592 | sll1592 | regulation of transcription, DNA-dependent | GO:0006355 |
| sll1687 | sll1687 | regulation of transcription, DNA-dependent | GO:0006355 |
| sll1924 | sll1924 | regulation of transcription, DNA-dependent | GO:0006355 |
| sll5059 | sll5059 | regulation of transcription, DNA-dependent | GO:0006355 |
| sll5086 | sll5086 | regulation of transcription, DNA-dependent | GO:0006355 |
| slr0115 | slr0115 | regulation of transcription, DNA-dependent | GO:0006355 |
| slr0302 | slr0302 | regulation of transcription, DNA-dependent | GO:0006355 |
| slr0311 | slr0311 | regulation of transcription, DNA-dependent | GO:0006355 |
| slr0312 | slr0312 | regulation of transcription, DNA-dependent | GO:0006355 |
| slr0359 | slr0359 | regulation of transcription, DNA-dependent | GO:0006355 |
| slr1041 | slr1041 | regulation of transcription, DNA-dependent | GO:0006355 |
| slr1214 | slr1214 | regulation of transcription, DNA-dependent | GO:0006355 |
| slr1305 | slr1305 | regulation of transcription, DNA-dependent | GO:0006355 |
| slr1588 | slr1588 | regulation of transcription, DNA-dependent | GO:0006355 |
| slr1594 | slr1594 | regulation of transcription, DNA-dependent | GO:0006355 |
| slr1693 | slr1693 | regulation of transcription, DNA-dependent | GO:0006355 |
| slr1837 | slr1837 | regulation of transcription, DNA-dependent | GO:0006355 |
| slr2100 | slr2100 | regulation of transcription, DNA-dependent | GO:0006355 |
| ssl1326 | ssl1326 | regulation of transcription, DNA-dependent | GO:0006355 |
| sll0998 | Probable RuBisCO transcriptional regulator | regulation of RNA metabolic process | GO:0051252 |
| slr2099 | Sensor protein | regulation of RNA metabolic process | GO:0051252 |
| slr1969 | Sensor protein | regulation of RNA metabolic process | GO:0051252 |
| slr6001 | Sensor protein | regulation of RNA metabolic process | GO:0051252 |
| slr2104 | Sensor protein | regulation of RNA metabolic process | GO:0051252 |
| slr1324 | Sensor protein | regulation of RNA metabolic process | GO:0051252 |
| slr2098 | Sensor protein | regulation of RNA metabolic process | GO:0051252 |
| sll1228 | Sensor protein | regulation of RNA metabolic process | GO:0051252 |
| slr0222 | Sensor protein | regulation of RNA metabolic process | GO:0051252 |
| sll0474 | Sensor protein | regulation of RNA metabolic process | GO:0051252 |
| sll1672 | Sensor protein | regulation of RNA metabolic process | GO:0051252 |
| sll1229 | Sensor protein | regulation of RNA metabolic process | GO:0051252 |
| sll5060 | Sensor protein | regulation of RNA metabolic process | GO:0051252 |
| slr1393 | Sensor protein | regulation of RNA metabolic process | GO:0051252 |
| sll1124 | Sensor protein | regulation of RNA metabolic process | GO:0051252 |
| sll1905 | Sensor protein | regulation of RNA metabolic process | GO:0051252 |
| sll1879 | Ycf55-like protein | regulation of RNA metabolic process | GO:0051252 |
| sll0038 | sll0038 | regulation of RNA metabolic process | GO:0051252 |
| sll0267 | sll0267 | regulation of RNA metabolic process | GO:0051252 |
| sll0779 | sll0779 | regulation of RNA metabolic process | GO:0051252 |
| sll0782 | sll0782 | regulation of RNA metabolic process | GO:0051252 |
| sll0797 | sll0797 | regulation of RNA metabolic process | GO:0051252 |
| sll1296 | sll1296 | regulation of RNA metabolic process | GO:0051252 |
| sll1330 | sll1330 | regulation of RNA metabolic process | GO:0051252 |
| sll1592 | sll1592 | regulation of RNA metabolic process | GO:0051252 |
| sll1687 | sll1687 | regulation of RNA metabolic process | GO:0051252 |
| sll1924 | sll1924 | regulation of RNA metabolic process | GO:0051252 |
| sll5059 | sll5059 | regulation of RNA metabolic process | GO:0051252 |
| sll5086 | sll5086 | regulation of RNA metabolic process | GO:0051252 |
| slr0115 | slr0115 | regulation of RNA metabolic process | GO:0051252 |
| slr0302 | slr0302 | regulation of RNA metabolic process | GO:0051252 |
| slr0311 | slr0311 | regulation of RNA metabolic process | GO:0051252 |
| slr0312 | slr0312 | regulation of RNA metabolic process | GO:0051252 |
| slr0359 | slr0359 | regulation of RNA metabolic process | GO:0051252 |
| slr1041 | slr1041 | regulation of RNA metabolic process | GO:0051252 |
| slr1214 | slr1214 | regulation of RNA metabolic process | GO:0051252 |
| slr1305 | slr1305 | regulation of RNA metabolic process | GO:0051252 |
| slr1588 | slr1588 | regulation of RNA metabolic process | GO:0051252 |
| slr1594 | slr1594 | regulation of RNA metabolic process | GO:0051252 |
| slr1693 | slr1693 | regulation of RNA metabolic process | GO:0051252 |
| slr1837 | slr1837 | regulation of RNA metabolic process | GO:0051252 |
| slr2100 | slr2100 | regulation of RNA metabolic process | GO:0051252 |
| ssl1326 | ssl1326 | regulation of RNA metabolic process | GO:0051252 |
| slr0640 | Sensor protein | peptidyl-histidine modification | GO:0018202 |
| sll1228 | Sensor protein | peptidyl-histidine modification | GO:0018202 |
| slr2099 | Sensor protein | peptidyl-histidine modification | GO:0018202 |
| slr1285 | Sensor protein | peptidyl-histidine modification | GO:0018202 |
| slr0222 | Sensor protein | peptidyl-histidine modification | GO:0018202 |
| slr1969 | Sensor protein | peptidyl-histidine modification | GO:0018202 |
| slr6001 | Sensor protein | peptidyl-histidine modification | GO:0018202 |
| slr1147 | Sensor protein | peptidyl-histidine modification | GO:0018202 |
| slr2104 | Sensor protein | peptidyl-histidine modification | GO:0018202 |
| sll0474 | Sensor protein | peptidyl-histidine modification | GO:0018202 |
| sll1672 | Sensor protein | peptidyl-histidine modification | GO:0018202 |
| sll1229 | Sensor protein | peptidyl-histidine modification | GO:0018202 |
| sll5060 | Sensor protein | peptidyl-histidine modification | GO:0018202 |
| sll0798 | Sensor protein | peptidyl-histidine modification | GO:0018202 |
| slr1393 | Sensor protein | peptidyl-histidine modification | GO:0018202 |
| sll1124 | Sensor protein | peptidyl-histidine modification | GO:0018202 |
| slr1324 | Sensor protein | peptidyl-histidine modification | GO:0018202 |
| sll1888 | Sensor protein | peptidyl-histidine modification | GO:0018202 |
| sll1905 | Sensor protein | peptidyl-histidine modification | GO:0018202 |
| slr2098 | Sensor protein | peptidyl-histidine modification | GO:0018202 |
| sll1296 | sll1296 | peptidyl-histidine modification | GO:0018202 |
| slr0311 | slr0311 | peptidyl-histidine modification | GO:0018202 |
| slr0640 | Sensor protein | peptidyl-histidine phosphorylation | GO:0018106 |
| sll1228 | Sensor protein | peptidyl-histidine phosphorylation | GO:0018106 |
| slr2099 | Sensor protein | peptidyl-histidine phosphorylation | GO:0018106 |
| slr1285 | Sensor protein | peptidyl-histidine phosphorylation | GO:0018106 |
| slr0222 | Sensor protein | peptidyl-histidine phosphorylation | GO:0018106 |
| slr1969 | Sensor protein | peptidyl-histidine phosphorylation | GO:0018106 |
| slr6001 | Sensor protein | peptidyl-histidine phosphorylation | GO:0018106 |
| slr1147 | Sensor protein | peptidyl-histidine phosphorylation | GO:0018106 |
| slr2104 | Sensor protein | peptidyl-histidine phosphorylation | GO:0018106 |
| sll0474 | Sensor protein | peptidyl-histidine phosphorylation | GO:0018106 |
| sll1672 | Sensor protein | peptidyl-histidine phosphorylation | GO:0018106 |
| sll1229 | Sensor protein | peptidyl-histidine phosphorylation | GO:0018106 |
| sll5060 | Sensor protein | peptidyl-histidine phosphorylation | GO:0018106 |
| sll0798 | Sensor protein | peptidyl-histidine phosphorylation | GO:0018106 |
| slr1393 | Sensor protein | peptidyl-histidine phosphorylation | GO:0018106 |
| sll1124 | Sensor protein | peptidyl-histidine phosphorylation | GO:0018106 |
| slr1324 | Sensor protein | peptidyl-histidine phosphorylation | GO:0018106 |
| sll1888 | Sensor protein | peptidyl-histidine phosphorylation | GO:0018106 |
| sll1905 | Sensor protein | peptidyl-histidine phosphorylation | GO:0018106 |
| slr2098 | Sensor protein | peptidyl-histidine phosphorylation | GO:0018106 |
| sll1296 | sll1296 | peptidyl-histidine phosphorylation | GO:0018106 |
| slr0311 | slr0311 | peptidyl-histidine phosphorylation | GO:0018106 |
| sll0998 | Probable RuBisCO transcriptional regulator | regulation of transcription | GO:0006355 |
| slr2099 | Sensor protein | regulation of transcription | GO:0006355 |
| slr1969 | Sensor protein | regulation of transcription | GO:0006355 |
| slr6001 | Sensor protein | regulation of transcription | GO:0006355 |
| slr2104 | Sensor protein | regulation of transcription | GO:0006355 |
| slr1324 | Sensor protein | regulation of transcription | GO:0006355 |
| slr2098 | Sensor protein | regulation of transcription | GO:0006355 |
| sll1228 | Sensor protein | regulation of transcription | GO:0006355 |
| slr0222 | Sensor protein | regulation of transcription | GO:0006355 |
| sll0474 | Sensor protein | regulation of transcription | GO:0006355 |
| sll1672 | Sensor protein | regulation of transcription | GO:0006355 |
| sll1229 | Sensor protein | regulation of transcription | GO:0006355 |
| sll5060 | Sensor protein | regulation of transcription | GO:0006355 |
| slr1393 | Sensor protein | regulation of transcription | GO:0006355 |
| sll1124 | Sensor protein | regulation of transcription | GO:0006355 |
| sll1905 | Sensor protein | regulation of transcription | GO:0006355 |
| sll1879 | Ycf55-like protein | regulation of transcription | GO:0006355 |
| sll0038 | sll0038 | regulation of transcription | GO:0006355 |
| sll0267 | sll0267 | regulation of transcription | GO:0006355 |
| sll0779 | sll0779 | regulation of transcription | GO:0006355 |
| sll0782 | sll0782 | regulation of transcription | GO:0006355 |
| sll0797 | sll0797 | regulation of transcription | GO:0006355 |
| sll1296 | sll1296 | regulation of transcription | GO:0006355 |
| sll1330 | sll1330 | regulation of transcription | GO:0006355 |
| sll1592 | sll1592 | regulation of transcription | GO:0006355 |
| sll1687 | sll1687 | regulation of transcription | GO:0006355 |
| sll1924 | sll1924 | regulation of transcription | GO:0006355 |
| sll5059 | sll5059 | regulation of transcription | GO:0006355 |
| sll5086 | sll5086 | regulation of transcription | GO:0006355 |
| slr0115 | slr0115 | regulation of transcription | GO:0006355 |
| slr0302 | slr0302 | regulation of transcription | GO:0006355 |
| slr0311 | slr0311 | regulation of transcription | GO:0006355 |
| slr0312 | slr0312 | regulation of transcription | GO:0006355 |
| slr0359 | slr0359 | regulation of transcription | GO:0006355 |
| slr1041 | slr1041 | regulation of transcription | GO:0006355 |
| slr1214 | slr1214 | regulation of transcription | GO:0006355 |
| slr1305 | slr1305 | regulation of transcription | GO:0006355 |
| slr1588 | slr1588 | regulation of transcription | GO:0006355 |
| slr1594 | slr1594 | regulation of transcription | GO:0006355 |
| slr1693 | slr1693 | regulation of transcription | GO:0006355 |
| slr1837 | slr1837 | regulation of transcription | GO:0006355 |
| slr2100 | slr2100 | regulation of transcription | GO:0006355 |
| ssl1326 | ssl1326 | regulation of transcription | GO:0006355 |
| sll0711 | 4-diphosphocytidyl-2-C-methyl-D-erythritol kinase | phosphorylation | GO:0016310 |
| slr0756 | Circadian clock protein kaiA | phosphorylation | GO:0016310 |
| slr2099 | Sensor protein | phosphorylation | GO:0016310 |
| slr1285 | Sensor protein | phosphorylation | GO:0016310 |
| slr1969 | Sensor protein | phosphorylation | GO:0016310 |
| slr6001 | Sensor protein | phosphorylation | GO:0016310 |
| slr2104 | Sensor protein | phosphorylation | GO:0016310 |
| slr1324 | Sensor protein | phosphorylation | GO:0016310 |
| sll1888 | Sensor protein | phosphorylation | GO:0016310 |
| slr2098 | Sensor protein | phosphorylation | GO:0016310 |
| slr0640 | Sensor protein | phosphorylation | GO:0016310 |
| sll1228 | Sensor protein | phosphorylation | GO:0016310 |
| slr0222 | Sensor protein | phosphorylation | GO:0016310 |
| slr1147 | Sensor protein | phosphorylation | GO:0016310 |
| sll0474 | Sensor protein | phosphorylation | GO:0016310 |
| sll1672 | Sensor protein | phosphorylation | GO:0016310 |
| sll1229 | Sensor protein | phosphorylation | GO:0016310 |
| sll5060 | Sensor protein | phosphorylation | GO:0016310 |
| sll0798 | Sensor protein | phosphorylation | GO:0016310 |
| slr1393 | Sensor protein | phosphorylation | GO:0016310 |
| sll1124 | Sensor protein | phosphorylation | GO:0016310 |
| sll1905 | Sensor protein | phosphorylation | GO:0016310 |
| sll1575 | Serine/threonine-protein kinase A | phosphorylation | GO:0016310 |
| slr0599 | Serine/threonine-protein kinase C | phosphorylation | GO:0016310 |
| sll1296 | sll1296 | phosphorylation | GO:0016310 |
| slr0152 | slr0152 | phosphorylation | GO:0016310 |
| slr0311 | slr0311 | phosphorylation | GO:0016310 |
| sll0711 | 4-diphosphocytidyl-2-C-methyl-D-erythritol kinase | phosphate metabolic process | GO:0006796 |
| slr0756 | Circadian clock protein kaiA | phosphate metabolic process | GO:0006796 |
| slr0328 | Putative low molecular weight protein-tyrosine-phosphatase slr0328 | phosphate metabolic process | GO:0006796 |
| slr2099 | Sensor protein | phosphate metabolic process | GO:0006796 |
| slr1285 | Sensor protein | phosphate metabolic process | GO:0006796 |
| slr1969 | Sensor protein | phosphate metabolic process | GO:0006796 |
| slr6001 | Sensor protein | phosphate metabolic process | GO:0006796 |
| slr2104 | Sensor protein | phosphate metabolic process | GO:0006796 |
| slr1324 | Sensor protein | phosphate metabolic process | GO:0006796 |
| sll1888 | Sensor protein | phosphate metabolic process | GO:0006796 |
| slr2098 | Sensor protein | phosphate metabolic process | GO:0006796 |
| slr0640 | Sensor protein | phosphate metabolic process | GO:0006796 |
| sll1228 | Sensor protein | phosphate metabolic process | GO:0006796 |
| slr0222 | Sensor protein | phosphate metabolic process | GO:0006796 |
| slr1147 | Sensor protein | phosphate metabolic process | GO:0006796 |
| sll0474 | Sensor protein | phosphate metabolic process | GO:0006796 |
| sll1672 | Sensor protein | phosphate metabolic process | GO:0006796 |
| sll1229 | Sensor protein | phosphate metabolic process | GO:0006796 |
| sll5060 | Sensor protein | phosphate metabolic process | GO:0006796 |
| sll0798 | Sensor protein | phosphate metabolic process | GO:0006796 |
| slr1393 | Sensor protein | phosphate metabolic process | GO:0006796 |
| sll1124 | Sensor protein | phosphate metabolic process | GO:0006796 |
| sll1905 | Sensor protein | phosphate metabolic process | GO:0006796 |
| sll1575 | Serine/threonine-protein kinase A | phosphate metabolic process | GO:0006796 |
| slr0599 | Serine/threonine-protein kinase C | phosphate metabolic process | GO:0006796 |
| sll1296 | sll1296 | phosphate metabolic process | GO:0006796 |
| slr0152 | slr0152 | phosphate metabolic process | GO:0006796 |
| slr0311 | slr0311 | phosphate metabolic process | GO:0006796 |
| sll0711 | 4-diphosphocytidyl-2-C-methyl-D-erythritol kinase | phosphorus metabolic process | GO:0006793 |
| slr0756 | Circadian clock protein kaiA | phosphorus metabolic process | GO:0006793 |
| slr0328 | Putative low molecular weight protein-tyrosine-phosphatase slr0328 | phosphorus metabolic process | GO:0006793 |
| slr2099 | Sensor protein | phosphorus metabolic process | GO:0006793 |
| slr1285 | Sensor protein | phosphorus metabolic process | GO:0006793 |
| slr1969 | Sensor protein | phosphorus metabolic process | GO:0006793 |
| slr6001 | Sensor protein | phosphorus metabolic process | GO:0006793 |
| slr2104 | Sensor protein | phosphorus metabolic process | GO:0006793 |
| slr1324 | Sensor protein | phosphorus metabolic process | GO:0006793 |
| sll1888 | Sensor protein | phosphorus metabolic process | GO:0006793 |
| slr2098 | Sensor protein | phosphorus metabolic process | GO:0006793 |
| slr0640 | Sensor protein | phosphorus metabolic process | GO:0006793 |
| sll1228 | Sensor protein | phosphorus metabolic process | GO:0006793 |
| slr0222 | Sensor protein | phosphorus metabolic process | GO:0006793 |
| slr1147 | Sensor protein | phosphorus metabolic process | GO:0006793 |
| sll0474 | Sensor protein | phosphorus metabolic process | GO:0006793 |
| sll1672 | Sensor protein | phosphorus metabolic process | GO:0006793 |
| sll1229 | Sensor protein | phosphorus metabolic process | GO:0006793 |
| sll5060 | Sensor protein | phosphorus metabolic process | GO:0006793 |
| sll0798 | Sensor protein | phosphorus metabolic process | GO:0006793 |
| slr1393 | Sensor protein | phosphorus metabolic process | GO:0006793 |
| sll1124 | Sensor protein | phosphorus metabolic process | GO:0006793 |
| sll1905 | Sensor protein | phosphorus metabolic process | GO:0006793 |
| sll1575 | Serine/threonine-protein kinase A | phosphorus metabolic process | GO:0006793 |
| slr0599 | Serine/threonine-protein kinase C | phosphorus metabolic process | GO:0006793 |
| sll1296 | sll1296 | phosphorus metabolic process | GO:0006793 |
| slr0152 | slr0152 | phosphorus metabolic process | GO:0006793 |
| slr0311 | slr0311 | phosphorus metabolic process | GO:0006793 |
| sll1946 | sll1946 | glycoprotein metabolic process | GO:0009100 |
| sll2003 | sll2003 | glycoprotein metabolic process | GO:0009100 |
| slr1820 | slr1820 | glycoprotein metabolic process | GO:0009100 |
| sll1946 | sll1946 | glycoprotein biosynthetic process | GO:0009101 |
| sll2003 | sll2003 | glycoprotein biosynthetic process | GO:0009101 |
| slr1820 | slr1820 | glycoprotein biosynthetic process | GO:0009101 |
| sll1946 | sll1946 | glycosylation | GO:0070085 |
| sll2003 | sll2003 | glycosylation | GO:0070085 |
| slr1820 | slr1820 | glycosylation | GO:0070085 |
| sll1946 | sll1946 | protein amino acid glycosylation | GO:0006486 |
| sll2003 | sll2003 | protein amino acid glycosylation | GO:0006486 |
| slr1820 | slr1820 | protein amino acid glycosylation | GO:0006486 |
| sll1946 | sll1946 | biopolymer glycosylation | GO:0043413 |
| sll2003 | sll2003 | biopolymer glycosylation | GO:0043413 |
| slr1820 | slr1820 | biopolymer glycosylation | GO:0043413 |
| sll1946 | sll1946 | protein amino acid O-linked glycosylation | GO:0006493 |
| sll2003 | sll2003 | protein amino acid O-linked glycosylation | GO:0006493 |
| slr1820 | slr1820 | protein amino acid O-linked glycosylation | GO:0006493 |

| **Table S5.** Complete list of enriched GO terms of target genes in *Synechocystis* sp. PCC 6803. | | | | | |
| --- | --- | --- | --- | --- | --- |
| **Category** | **GO_ID** | **Description** | **P-value** | **Count** | **Genes in test set** |
| Biological Process | GO:0000160 | two-component signal transduction system (phosphorelay) | 5.00622E-13 | 39 | SLR1693, SLR2099, SLL0267, SLL5060, SLR2098, SLL1672, SLL1296, SLL1592, SLL1124, SLL1879, SLR1594, SLL1228, SLL1229, SLL0797, SLL0779, SLR2100, SLR1324, SLR2104, SLL0038, SLR0302, SLL1905, SLR1837, SLR1041, SLR0222, SLR6001, SLR1214, SLR1393, SLR1305, SLR1588, SLL1687, SLR1969, SLR0312, SLR0311, SLL0474, SLL1330, SLL5059, SLR0115, SLL0782, SLR0359 |
| Biological Process | GO:0006468 | protein amino acid phosphorylation | 1.55071E-09 | 26 | SLR0640, SLR2099, SLR2098, SLL5060, SLL1296, SLL1672, SLL1575, SLL1124, SLL1228, SLL1229, SLL0798, SLR1324, SLR1285, SLR1147, SLR2104, SLL1905, SLR0599, SLR0756, SLR0222, SLR6001, SLL1888, SLR1393, SLR1969, SLR0311, SLL0474, SLR0152 |
| Biological Process | GO:0051252 | regulation of RNA metabolic process | 2.22527E-09 | 43 | SLR1693, SLR2099, SLL0267, SLL5060, SLL5086, SLR2098, SLL1672, SLL1296, SLL0998, SLL1592, SLL1124, SLL1879, SLR1594, SLL1228, SLL1229, SLL0797, SLL0779, SLR2100, SLR1324, SLR2104, SLL0038, SLR0302, SLL1905, SLR1837, SLR1041, SLR0222, SLL1924, SLR6001, SLR1214, SLR1393, SSL1326, SLR1305, SLR1588, SLL1687, SLR1969, SLR0312, SLR0311, SLL0474, SLL1330, SLL5059, SLR0115, SLR0359, SLL0782 |
| Biological Process | GO:0006355 | regulation of transcription, DNA-dependent | 2.22527E-09 | 43 | SLR1693, SLR2099, SLL0267, SLL5060, SLL5086, SLR2098, SLL1672, SLL1296, SLL0998, SLL1592, SLL1124, SLL1879, SLR1594, SLL1228, SLL1229, SLL0797, SLL0779, SLR2100, SLR1324, SLR2104, SLL0038, SLR0302, SLL1905, SLR1837, SLR1041, SLR0222, SLL1924, SLR6001, SLR1214, SLR1393, SSL1326, SLR1305, SLR1588, SLL1687, SLR1969, SLR0312, SLR0311, SLL0474, SLL1330, SLL5059, SLR0115, SLR0359, SLL0782 |
| Biological Process | GO:0018106 | peptidyl-histidine phosphorylation | 4.66462E-08 | 22 | SLR0222, SLR0640, SLR6001, SLR2099, SLR2098, SLL5060, SLL1888, SLL1672, SLL1296, SLR1393, SLL1124, SLL1228, SLL1229, SLL0798, SLR1324, SLR1285, SLR1147, SLR2104, SLR1969, SLR0311, SLL0474, SLL1905 |
| Biological Process | GO:0018202 | peptidyl-histidine modification | 4.66462E-08 | 22 | SLR0222, SLR0640, SLR6001, SLR2099, SLR2098, SLL5060, SLL1888, SLL1672, SLL1296, SLR1393, SLL1124, SLL1228, SLL1229, SLL0798, SLR1324, SLR1285, SLR1147, SLR2104, SLR1969, SLR0311, SLL0474, SLL1905 |
| Biological Process | GO:0045449 | regulation of transcription | 6.32384E-08 | 43 | SLR1693, SLR2099, SLL0267, SLL5060, SLL5086, SLR2098, SLL1672, SLL1296, SLL0998, SLL1592, SLL1124, SLL1879, SLR1594, SLL1228, SLL1229, SLL0797, SLL0779, SLR2100, SLR1324, SLR2104, SLL0038, SLR0302, SLL1905, SLR1837, SLR1041, SLR0222, SLL1924, SLR6001, SLR1214, SLR1393, SSL1326, SLR1305, SLR1588, SLL1687, SLR1969, SLR0312, SLR0311, SLL0474, SLL1330, SLL5059, SLR0115, SLR0359, SLL0782 |
| Biological Process | GO:0016310 | phosphorylation | 3.65282E-06 | 27 | SLR0640, SLR2099, SLR2098, SLL5060, SLL1296, SLL1672, SLL1575, SLL1124, SLL1228, SLL1229, SLL0798, SLR1324, SLR1285, SLR1147, SLR2104, SLL1905, SLR0599, SLR0756, SLR0222, SLR6001, SLL0711, SLL1888, SLR1393, SLR1969, SLR0311, SLL0474, SLR0152 |
| Biological Process | GO:0006793 | phosphorus metabolic process | 8.57981E-06 | 28 | SLR0640, SLR2099, SLR2098, SLL5060, SLL1296, SLL1672, SLL1575, SLL1124, SLL1228, SLL1229, SLL0798, SLR1324, SLR1285, SLR1147, SLR0328, SLR2104, SLL1905, SLR0599, SLR0756, SLR0222, SLR6001, SLL0711, SLL1888, SLR1393, SLR1969, SLR0311, SLL0474, SLR0152 |
| Biological Process | GO:0006796 | phosphate metabolic process | 8.57981E-06 | 28 | SLR0640, SLR2099, SLR2098, SLL5060, SLL1296, SLL1672, SLL1575, SLL1124, SLL1228, SLL1229, SLL0798, SLR1324, SLR1285, SLR1147, SLR0328, SLR2104, SLL1905, SLR0599, SLR0756, SLR0222, SLR6001, SLL0711, SLL1888, SLR1393, SLR1969, SLR0311, SLL0474, SLR0152 |
| Cellular Component | GO:0030288 | outer membrane-bounded periplasmic space | 0.001315068 | 23 | SLL1532, SLR1406, SLL1835, SLL0064, SLR0924, SLL1549, SLR1940, SLL0224, SLL1491, SLR0513, SLR1196, SLL1507, SLL1483, SLR2048, SLR1668, SLR0401, SLR1924, SLR1484, SLL0837, SLR2004, SLL1089, SLR0708, SLR1257 |
| Cellular Component | GO:0042597 | periplasmic space | 0.00164317 | 23 | SLL1532, SLR1406, SLL1835, SLL0064, SLR0924, SLL1549, SLR1940, SLL0224, SLL1491, SLR0513, SLR1196, SLL1507, SLL1483, SLR2048, SLR1668, SLR0401, SLR1924, SLR1484, SLL0837, SLR2004, SLL1089, SLR0708, SLR1257 |
| Cellular Component | GO:0044462 | external encapsulating structure part | 0.002515327 | 23 | SLL1532, SLR1406, SLL1835, SLL0064, SLR0924, SLL1549, SLR1940, SLL0224, SLL1491, SLR0513, SLR1196, SLL1507, SLL1483, SLR2048, SLR1668, SLR0401, SLR1924, SLR1484, SLL0837, SLR2004, SLL1089, SLR0708, SLR1257 |
| Cellular Component | GO:0031975 | envelope | 0.010933815 | 23 | SLL1532, SLR1406, SLL1835, SLL0064, SLR0924, SLL1549, SLR1940, SLL0224, SLL1491, SLR0513, SLR1196, SLL1507, SLL1483, SLR2048, SLR1668, SLR0401, SLR1924, SLR1484, SLL0837, SLR2004, SLL1089, SLR0708, SLR1257 |
| Cellular Component | GO:0030313 | cell envelope | 0.010933815 | 23 | SLL1532, SLR1406, SLL1835, SLL0064, SLR0924, SLL1549, SLR1940, SLL0224, SLL1491, SLR0513, SLR1196, SLL1507, SLL1483, SLR2048, SLR1668, SLR0401, SLR1924, SLR1484, SLL0837, SLR2004, SLL1089, SLR0708, SLR1257 |
| Cellular Component | GO:0031224 | intrinsic to membrane | 0.0169626 | 59 | SLL1550, SLL0528, SLR1841, SLR0681, SLR1201, SLL0615, SLL1428, SLR1182, SLR1488, SLR1647, SLR1149, SLR0615, SLR1790, SLR0594, SLR5078, SLL1271, SLR0822, SLR0658, SLL0182, SLL1081, SLR5005, SLR0712, SLL1725, SLR2131, SLL0474, SLL1147, SLL0477, SLR1336, SLL1087, SLL0875, SLL0063, SLR0273, SLR0272, SLR0643, SLR0944, SLL0501, SLL0672, SLR0896, SLR1776, SLL0060, SLR1163, SLR0794, SLR0639, SLL0556, SLR1908, SLR0096, SLR1494, SLL1060, SLL0862, SLR0269, SLR1403, SLR0408, SLR1028, SSL3127, SLL1021, SLR1949, SLR0311, SLL0312, SLR6043 |
| Cellular Component | GO:0005887 | integral to plasma membrane | 0.018343474 | 4 | SLR5005, SLR5078, SLR1403, SLR1028 |
| Cellular Component | GO:0031226 | intrinsic to plasma membrane | 0.018343474 | 4 | SLR5005, SLR5078, SLR1403, SLR1028 |
| Cellular Component | GO:0030312 | external encapsulating structure | 0.023271403 | 23 | SLL1532, SLR1406, SLL1835, SLL0064, SLR0924, SLL1549, SLR1940, SLL0224, SLL1491, SLR0513, SLR1196, SLL1507, SLL1483, SLR2048, SLR1668, SLR0401, SLR1924, SLR1484, SLL0837, SLR2004, SLL1089, SLR0708, SLR1257 |
| Cellular Component | GO:0016021 | integral to membrane | 0.024425451 | 58 | SLL1550, SLL0528, SLR1841, SLR0681, SLR1201, SLL0615, SLL1428, SLR1182, SLR1488, SLR1647, SLR1149, SLR0615, SLR1790, SLR0594, SLR5078, SLL1271, SLR0822, SLL0182, SLL1081, SLR5005, SLR0712, SLL1725, SLR2131, SLL0474, SLL1147, SLL0477, SLR1336, SLL1087, SLL0875, SLL0063, SLR0273, SLR0272, SLR0643, SLR0944, SLL0501, SLL0672, SLR0896, SLR1776, SLL0060, SLR1163, SLR0794, SLR0639, SLL0556, SLR1908, SLR0096, SLR1494, SLL1060, SLL0862, SLR0269, SLR1403, SLR0408, SLR1028, SSL3127, SLL1021, SLR1949, SLR0311, SLL0312, SLR6043 |
| Molecular Function | GO:0004672 | protein kinase activity | 4.62532E-10 | 32 | SLR0640, SLR2099, SLR2098, SLL5060, SLL0267, SLL1672, SLL1296, SLL1575, SLL1124, SLL1228, SLL1229, SLL0779, SLL0798, SLR1324, SLR1285, SLR1147, SLR2104, SLR0302, SLL1905, SLR0599, SLR0222, SLR6001, SLL1888, SLR1393, SLR1305, SLL1687, SLL1334, SLR1969, SLR0311, SLL0474, SLR0152, SLR0359 |
| Molecular Function | GO:0016775 | phosphotransferase activity, nitrogenous group as acceptor | 9.61027E-10 | 29 | SLR0640, SLR2099, SLR2098, SLL0267, SLL5060, SLL1296, SLL1672, SLL1124, SLL1228, SLL1229, SLL0779, SLL0798, SLR1324, SLR1285, SLR1147, SLR2104, SLR0302, SLL1905, SLR0222, SLR6001, SLL1888, SLR1393, SLR1305, SLL1687, SLL1334, SLR1969, SLR0311, SLL0474, SLR0359 |
| Molecular Function | GO:0000155 | two-component sensor activity | 9.61027E-10 | 29 | SLR0640, SLR2099, SLR2098, SLL0267, SLL5060, SLL1296, SLL1672, SLL1124, SLL1228, SLL1229, SLL0779, SLL0798, SLR1324, SLR1285, SLR1147, SLR2104, SLR0302, SLL1905, SLR0222, SLR6001, SLL1888, SLR1393, SLR1305, SLL1687, SLL1334, SLR1969, SLR0311, SLL0474, SLR0359 |
| Molecular Function | GO:0004673 | protein histidine kinase activity | 9.61027E-10 | 29 | SLR0640, SLR2099, SLR2098, SLL0267, SLL5060, SLL1296, SLL1672, SLL1124, SLL1228, SLL1229, SLL0779, SLL0798, SLR1324, SLR1285, SLR1147, SLR2104, SLR0302, SLL1905, SLR0222, SLR6001, SLL1888, SLR1393, SLR1305, SLL1687, SLL1334, SLR1969, SLR0311, SLL0474, SLR0359 |
| Molecular Function | GO:0000156 | two-component response regulator activity | 2.49032E-09 | 31 | SLR1693, SLR2099, SLR2098, SLL5060, SLL1296, SLL1672, SLL1592, SLL1879, SLR1594, SLL1228, SLL1229, SLL0797, SLR2100, SLR1324, SLR2104, SLL0038, SLL1905, SLR1837, SLR1041, SLR0222, SLR6001, SLR1214, SLR1588, SLR1305, SLR1969, SLR0312, SLL1330, SLL5059, SLL0474, SLR0115, SLL0782 |
| Molecular Function | GO:0030528 | transcription regulator activity | 1.18807E-05 | 35 | SLR1693, SLR2099, SLL5060, SLL5086, SLR2098, SLL1672, SLL1296, SLL0998, SLL1592, SLL1879, SLR1594, SLL1228, SLL1229, SLL0797, SLR2100, SLR1324, SLR2104, SLL0038, SLL1905, SLR1837, SLR1041, SLR0222, SLR6001, SLL1924, SLR1214, SLR1305, SLR1588, SLR0317, SLR1969, SLR0312, SLL0474, SLL1330, SLL5059, SLR0115, SLL0782 |
| Molecular Function | GO:0032559 | adenyl ribonucleotide binding | 0.017892333 | 59 | SLL5060, SLR1882, SLL0086, SLR0354, SLL1124, SLL1228, SLL1229, SLL0798, SLR1488, SLR1147, SLR1149, SLR0615, SLR7010, SLR0822, SLL1366, SLR0599, SLL0182, SLL0711, SLL8049, SLL1725, SLR1969, SLR0864, SLL0912, SLL0474, SLR1278, SLL5083, SLR0086, SLR6012, SLR0640, SLR2099, SLR0374, SLR2098, SLL0385, SLL1296, SLL1672, SLL1575, SLR0904, SLL1878, SLR0544, SLR1324, SLL0672, SLL0484, SLR2104, SLL1905, SLR2002, SLL0489, SLR0222, SLL0257, SLR6001, SLR1494, SLL1888, SLR1393, SLR6007, SLR6102, SLR1113, SLR6047, SLR0787, SLR0311, SLR0152 |
| Molecular Function | GO:0005524 | ATP binding | 0.017892333 | 59 | SLL5060, SLR1882, SLL0086, SLR0354, SLL1124, SLL1228, SLL1229, SLL0798, SLR1488, SLR1147, SLR1149, SLR0615, SLR7010, SLR0822, SLL1366, SLR0599, SLL0182, SLL0711, SLL8049, SLL1725, SLR1969, SLR0864, SLL0912, SLL0474, SLR1278, SLL5083, SLR0086, SLR6012, SLR0640, SLR2099, SLR0374, SLR2098, SLL0385, SLL1296, SLL1672, SLL1575, SLR0904, SLL1878, SLR0544, SLR1324, SLL0672, SLL0484, SLR2104, SLL1905, SLR2002, SLL0489, SLR0222, SLL0257, SLR6001, SLR1494, SLL1888, SLR1393, SLR6007, SLR6102, SLR1113, SLR6047, SLR0787, SLR0311, SLR0152 |
| Molecular Function | GO:0032553 | ribonucleotide binding | 0.030727144 | 62 | SLL5060, SLR1882, SLL0086, SLR0354, SLL1124, SLL0898, SLL1228, SLL1229, SLL0798, SLR1488, SLR1147, SLR1149, SLR0615, SLR7010, SLR0822, SLL1366, SLR0599, SLL0182, SLL0711, SLL8049, SLL1725, SLR1969, SLL0912, SLR0864, SLL0474, SLR1278, SLL5083, SLL0245, SLR0086, SLR6012, SLR0640, SLR2099, SLR0374, SLR2098, SLL0385, SLL1672, SLL1296, SLL1575, SLL1878, SLR0904, SLR0544, SLR1462, SLR1324, SLL0484, SLL0672, SLR2104, SLL1905, SLR2002, SLL0489, SLR0222, SLL0257, SLR6001, SLR1494, SLL1888, SLR1393, SLR6102, SLR6007, SLR1113, SLR6047, SLR0787, SLR0311, SLR0152 |
| Molecular Function | GO:0032555 | purine ribonucleotide binding | 0.030727144 | 62 | SLL5060, SLR1882, SLL0086, SLR0354, SLL1124, SLL0898, SLL1228, SLL1229, SLL0798, SLR1488, SLR1147, SLR1149, SLR0615, SLR7010, SLR0822, SLL1366, SLR0599, SLL0182, SLL0711, SLL8049, SLL1725, SLR1969, SLL0912, SLR0864, SLL0474, SLR1278, SLL5083, SLL0245, SLR0086, SLR6012, SLR0640, SLR2099, SLR0374, SLR2098, SLL0385, SLL1672, SLL1296, SLL1575, SLL1878, SLR0904, SLR0544, SLR1462, SLR1324, SLL0484, SLL0672, SLR2104, SLL1905, SLR2002, SLL0489, SLR0222, SLL0257, SLR6001, SLR1494, SLL1888, SLR1393, SLR6102, SLR6007, SLR1113, SLR6047, SLR0787, SLR0311, SLR0152 |
| Molecular Function | GO:0001883 | purine nucleoside binding | 0.03078996 | 61 | SLL0703, SLL5060, SLR1882, SLL0086, SLR0354, SLL1124, SLL1228, SLL1229, SLL0798, SLR1488, SLR1147, SLR1149, SLR0615, SLR7010, SLR0822, SLL1366, SLR0599, SLL0182, SLL0711, SLL8049, SLL1725, SLR1969, SLL0912, SLR0864, SLL0474, SLR1278, SLL5083, SLR0086, SLR6012, SLR0640, SLR2099, SLR0374, SLR2098, SLR0644, SLL0385, SLL1672, SLL1296, SLL1575, SLL1878, SLR0904, SLR0544, SLR1324, SLL0672, SLL0484, SLR2104, SLL1905, SLR2002, SLL0489, SLR0222, SLL0257, SLR6001, SLR1494, SLL1888, SLR1393, SLR6007, SLR6102, SLR1113, SLR6047, SLR0787, SLR0311, SLR0152 |
| Molecular Function | GO:0030554 | adenyl nucleotide binding | 0.03078996 | 61 | SLL0703, SLL5060, SLR1882, SLL0086, SLR0354, SLL1124, SLL1228, SLL1229, SLL0798, SLR1488, SLR1147, SLR1149, SLR0615, SLR7010, SLR0822, SLL1366, SLR0599, SLL0182, SLL0711, SLL8049, SLL1725, SLR1969, SLL0912, SLR0864, SLL0474, SLR1278, SLL5083, SLR0086, SLR6012, SLR0640, SLR2099, SLR0374, SLR2098, SLR0644, SLL0385, SLL1672, SLL1296, SLL1575, SLL1878, SLR0904, SLR0544, SLR1324, SLL0672, SLL0484, SLR2104, SLL1905, SLR2002, SLL0489, SLR0222, SLL0257, SLR6001, SLR1494, SLL1888, SLR1393, SLR6007, SLR6102, SLR1113, SLR6047, SLR0787, SLR0311, SLR0152 |
| Molecular Function | GO:0001882 | nucleoside binding | 0.039476213 | 61 | SLL0703, SLL5060, SLR1882, SLL0086, SLR0354, SLL1124, SLL1228, SLL1229, SLL0798, SLR1488, SLR1147, SLR1149, SLR0615, SLR7010, SLR0822, SLL1366, SLR0599, SLL0182, SLL0711, SLL8049, SLL1725, SLR1969, SLL0912, SLR0864, SLL0474, SLR1278, SLL5083, SLR0086, SLR6012, SLR0640, SLR2099, SLR0374, SLR2098, SLR0644, SLL0385, SLL1672, SLL1296, SLL1575, SLL1878, SLR0904, SLR0544, SLR1324, SLL0672, SLL0484, SLR2104, SLL1905, SLR2002, SLL0489, SLR0222, SLL0257, SLR6001, SLR1494, SLL1888, SLR1393, SLR6007, SLR6102, SLR1113, SLR6047, SLR0787, SLR0311, SLR0152 |
| Molecular Function | GO:0017076 | purine nucleotide binding | 0.049146149 | 64 | SLL0703, SLL5060, SLR1882, SLL0086, SLR0354, SLL1124, SLL0898, SLL1228, SLL1229, SLL0798, SLR1488, SLR1147, SLR1149, SLR0615, SLR7010, SLR0822, SLL1366, SLR0599, SLL0182, SLL0711, SLL8049, SLL1725, SLR1969, SLL0912, SLR0864, SLL0474, SLR1278, SLL5083, SLL0245, SLR0086, SLR6012, SLR0640, SLR2099, SLR0374, SLR2098, SLR0644, SLL0385, SLL1672, SLL1296, SLL1575, SLL1878, SLR0904, SLR0544, SLR1462, SLR1324, SLL0484, SLL0672, SLR2104, SLL1905, SLR2002, SLL0489, SLR0222, SLL0257, SLR6001, SLR1494, SLL1888, SLR1393, SLR6102, SLR6007, SLR1113, SLR6047, SLR0787, SLR0311, SLR0152 |

| **Table S6.** Complete list of KEGG pathways in the identified target genes of small regulatory RNAs. | |
| --- | --- |
| **syn01100 Metabolic pathways - *Synechocystis* sp. PCC 6803 (198)** | |
| syn:sll0017 | hemL; glutamate-1-semialdehyde aminotransferase (EC:5.4.3.8) |
| syn:sll0080 | argC; N-acetyl-gamma-glutamyl-phosphate reductase (EC:1.2.1.38) |
| syn:sll0135 | 5'-methylthioadenosine phosphorylase (EC:2.4.2.28) |
| syn:sll0144 | pyrH; uridylate kinase |
| syn:sll0158 | glgB; glycogen branching protein (EC:2.4.1.18) |
| syn:sll0166 | hemD; uroporphyrin-III synthase |
| syn:sll0179 | gltX; glutamyl-tRNA synthetase (EC:6.1.1.17) |
| syn:sll0207 | rfbA; glucose-1-phosphate thymidylyltransferase |
| syn:sll0220 | glmS; glucosamine--fructose-6-phosphate aminotransferase (EC:2.6.1.16) |
| syn:sll0244 | galE; UDP-glucose-4-epimerase |
| syn:sll0370 | carB; carbamoyl phosphate synthase large subunit (EC:6.3.5.5) |
| syn:sll0401 | gltA; citrate synthase (EC:2.3.3.5) |
| syn:sll0402 | aspC; aspartate aminotransferase (EC:2.6.1.1) |
| syn:sll0421 | purB; adenylosuccinate lyase (EC:4.3.2.2) |
| syn:sll0422 | asparaginase |
| syn:sll0427 | psbO; photosystem II manganese-stabilizing polypeptide |
| syn:sll0461 | proA; gamma-glutamyl phosphate reductase |
| syn:sll0469 | prsA; ribose-phosphate pyrophosphokinase (EC:2.7.6.1) |
| syn:sll0480 | L,L-diaminopimelate aminotransferase |
| syn:sll0504 | lysA; diaminopimelate decarboxylase |
| syn:sll0519 | ndhA; NADH dehydrogenase subunit H (EC:1.6.5.3) |
| syn:sll0544 | DNA polymerase III subunit delta |
| syn:sll0578 | purK; phosphoribosylaminoimidazole carboxylase ATPase subunit (EC:4.1.1.21) |
| syn:sll0587 | pykF; pyruvate kinase |
| syn:sll0593 | glk; glucokinase (EC:2.7.1.2) |
| syn:sll0603 | menD; 2-succinyl-5-enolpyruvyl-6-hydroxy-3-cyclohexene-1-carboxylate synthase (EC:2.2.1.9 4.1.1.71) |
| syn:sll0622 | nadA; quinolinate synthetase |
| syn:sll0629 | psaK; photosystem I subunit X |
| syn:sll0631 | nadB; L-aspartate oxidase (EC:1.4.3.16) |
| syn:sll0635 | thiE; thiamine-phosphate pyrophosphorylase (EC:2.5.1.3) |
| syn:sll0660 | pdxA; 4-hydroxythreonine-4-phosphate dehydrogenase (EC:1.1.1.262) |
| syn:sll0711 | 4-diphosphocytidyl-2-C-methyl-D-erythritol kinase (EC:2.7.1.148) |
| syn:sll0712 | cysM; cysteine synthase A |
| syn:sll0728 | accA; acetyl-CoA carboxylase carboxyltransferase subunit alpha (EC:6.4.1.2) |
| syn:sll0794 | merR; mercuric resistance operon regulatory protein precorrin isomerase |
| syn:sll0823 | sdhB; succinate dehydrogenase iron-sulfur subunit (EC:1.3.99.1) |
| syn:sll0838 | pyrF; orotidine 5'-phosphate decarboxylase (EC:4.1.1.23) |
| syn:sll0892 | panD; aspartate alpha-decarboxylase (EC:4.1.1.11) |
| syn:sll0895 | cysQ; ammonium transporter |
| syn:sll0927 | metX; S-adenosylmethionine synthetase (EC:2.5.1.6) |
| syn:sll0928 | apcD; allophycocyanin-B |
| syn:sll0934 | ccmA; 3-deoxy-7-phosphoheptulonate synthase |
| syn:sll1018 | pyrC; dihydroorotase (EC:3.5.2.3) |
| syn:sll1023 | sucC; succinate--CoA ligase |
| syn:sll1051 | cpcF; phycocyanin alpha phycocyanobilin lyase CpcF |
| syn:sll1056 | purL; phosphoribosylformylglycinamidine synthase II (EC:6.3.5.3) |
| syn:sll1059 | adk; adenylate kinase |
| syn:sll1070 | tktA; transketolase (EC:2.2.1.1) |
| syn:sll1077 | speB; agmatine ureohydrolase |
| syn:sll1108 | surE; stationary phase survival protein SurE (EC:3.1.3.2) |
| syn:sll1172 | thrC; threonine synthase (EC:4.2.3.1) |
| syn:sll1214 | magnesium-protoporphyrin IX monomethyl ester cyclase |
| syn:sll1220 | bidirectional hydrogenase complex protein HoxE |
| syn:sll1234 | ahcY; S-adenosyl-L-homocysteine hydrolase (EC:3.3.1.1) |
| syn:sll1317 | petA; apocytochrome f |
| syn:sll1329 | monophosphatase |
| syn:sll1343 | ape2; aminopeptidase |
| syn:sll1360 | dnaX; DNA polymerase III subunit |
| syn:sll1415 | ppnK; inorganic polyphosphate/ATP-NAD kinase (EC:2.7.1.23) |
| syn:sll1443 | pyrG; CTP synthetase (EC:6.3.4.2) |
| syn:sll1459 | surE; stationary phase survival protein SurE (EC:3.1.3.2) |
| syn:sll1470 | leuC; isopropylmalate isomerase large subunit (EC:4.2.1.33) |
| syn:sll1479 | devB; 6-phosphogluconolactonase |
| syn:sll1496 | mannose-1-phosphate guanylyltransferase |
| syn:sll1498 | carA; carbamoyl phosphate synthase small subunit (EC:6.3.5.5) |
| syn:sll1538 | bgl; beta-glucosidase |
| syn:sll1540 | dpm1; dolichyl-phosphate-mannose synthase |
| syn:sll1556 | isopentenyl pyrophosphate isomerase (EC:5.3.3.2) |
| syn:sll1557 | sucD; succinyl-CoA synthase subunit beta |
| syn:sll1578 | cpcA; phycocyanin a subunit |
| syn:sll1580 | cpcC; phycocyanin associated linker protein |
| syn:sll1605 | fabZ; (3R)-hydroxymyristoyl-ACP dehydratase |
| syn:sll1612 | folC; folyl-polyglutamate synthetase |
| syn:sll1625 | sdhB; succinate dehydrogenase iron-sulfur subunit (EC:1.3.99.1) |
| syn:sll1655 | birA; biotin [acetyl-CoA-carboxylase] ligase |
| syn:sll1721 | pdhB; Pyruvate dehydrogenase E1 component subunit beta |
| syn:sll1732 | ndhF; NAD(P)H-quinone oxidoreductase subunit F |
| syn:sll1733 | ndhD3; NAD(P)H-quinone oxidoreductase subunit M |
| syn:sll1750 | ureC; urease subunit alpha (EC:3.5.1.5) |
| syn:sll1760 | thrB; homoserine kinase (EC:2.7.1.39) |
| syn:sll1787 | rpoB; DNA-directed RNA polymerase subunit beta (EC:2.7.7.6) |
| syn:sll1818 | rpoA; DNA-directed RNA polymerase subunit alpha (EC:2.7.7.6) |
| syn:sll1841 | odhB; branched-chain alpha-keto acid dehydrogenase E2 |
| syn:sll1883 | argJ; bifunctional ornithine acetyltransferase/N-acetylglutamate synthase (EC:2.3.1.1 2.3.1.35) |
| syn:sll1893 | hisF; imidazole glycerol phosphate synthase subunit HisF |
| syn:sll1894 | ribA; bifunctional 3,4-dihydroxy-2-butanone 4-phosphate synthase/GTP cyclohydrolase II (EC:3.5.4.25) |
| syn:sll1899 | ctaB; protoheme IX farnesyltransferase |
| syn:sll1908 | serA; D-3-phosphoglycerate dehydrogenase |
| syn:sll1945 | 1-deoxy-D-xylulose-5-phosphate synthase (EC:2.2.1.7) |
| syn:sll1981 | ilvB; acetolactate synthase |
| syn:sll1987 | katG; catalase HPI |
| syn:sll1994 | hemB; delta-aminolevulinic acid dehydratase (EC:4.2.1.24) |
| syn:sll2001 | lap; leucyl aminopeptidase (EC:3.4.11.1) |
| syn:sll5079 | hypothetical protein |
| syn:slr0009 | rbcL; ribulose bisophosphate carboxylase (EC:4.1.1.39) |
| syn:slr0017 | murZ; UDP-N-acetylglucosamine 1-carboxyvinyltransferase (EC:2.5.1.7) |
| syn:slr0054 | dgkA; diacylglycerol kinase |
| syn:slr0056 | chlG; bacteriochlorophyll/chlorophyll a synthase |
| syn:slr0084 | hisH; imidazole glycerol phosphate synthase subunit HisH |
| syn:slr0090 | ppd; 4-hydroxyphenylpyruvic acid dioxgenase |
| syn:slr0091 | aldehyde dehydrogenase |
| syn:slr0204 | hypothetical protein |
| syn:slr0212 | metH; 5-methyltetrahydrofolate--homocysteine methyltransferase |
| syn:slr0237 | glgX; glycogen operon protein GlgX |
| syn:slr0252 | cobK; cobalt-precorrin-6x reductase (EC:1.3.1.54) |
| syn:slr0261 | ndhH; NAD(P)H-quinone oxidoreductase subunit H |
| syn:slr0301 | ppsA; phosphoenolpyruvate synthase (EC:2.7.9.2) |
| syn:slr0335 | apcE; phycobilisome LCM core-membrane linker polypeptide |
| syn:slr0402 | deoxyribonucleotide triphosphate pyrophosphatase |
| syn:slr0452 | ilvD; dihydroxy-acid dehydratase (EC:4.2.1.9) |
| syn:slr0458 | hypothetical protein |
| syn:slr0500 | hisB; imidazoleglycerol-phosphate dehydratase (EC:4.2.1.19) |
| syn:slr0536 | hemE; uroporphyrinogen decarboxylase (EC:4.1.1.37) |
| syn:slr0546 | trpC; indole-3-glycerol phosphate synthase (EC:4.1.1.48) |
| syn:slr0549 | asd; aspartate beta-semialdehyde dehydrogenese |
| syn:slr0550 | dapA; dihydrodipicolinate synthase (EC:4.2.1.52) |
| syn:slr0585 | argG; argininosuccinate synthase (EC:6.3.4.5) |
| syn:slr0597 | purH; bifunctional phosphoribosylaminoimidazolecarboxamide formyltransferase/IMP cyclohydrolase (EC:2.1.2.3 3.5.4.10) |
| syn:slr0603 | dnaE; DNA polymerase III subunit alpha |
| syn:slr0608 | hisI; bifunctional phosphoribosyl-AMP cyclohydrolase/phosphoribosyl-ATP (EC:3.5.4.19 3.6.1.31) |
| syn:slr0657 | lysC; aspartate kinase (EC:2.7.2.4) |
| syn:slr0662 | speA; arginine decarboxylase (EC:4.1.1.19) |
| syn:slr0665 | bifunctional aconitate hydratase 2/2-methylisocitrate dehydratase (EC:4.2.1.3) |
| syn:slr0682 | hisD; histidinol dehydrogenase |
| syn:slr0738 | trpE; anthranilate synthase component I |
| syn:slr0750 | chlN; light-independent protochlorophyllide reductase subunit N |
| syn:slr0787 | bifunctional nicotinamide mononucleotide adenylyltransferase/ADP-ribose pyrophosphatase (EC:2.7.7.1) |
| syn:slr0836 | rfbB; dTDP-glucose 4,6-dehydratase |
| syn:slr0838 | purM; phosphoribosylaminoimidazole synthetase (EC:6.3.3.1) |
| syn:slr0844 | ndhF; NAD(P)H-quinone oxidoreductase subunit F |
| syn:slr0847 | coaD; phosphopantetheine adenylyltransferase (EC:2.7.7.3) |
| syn:slr0897 | endo-1,4-beta-glucanase |
| syn:slr0917 | bioF; 8-amino-7-oxononanoate synthase (EC:2.3.1.47) |
| syn:slr0936 | nadC; nicotinate-nucleotide pyrophosphorylase (EC:2.4.2.19) |
| syn:slr0940 | crtQ; zeta-carotene desaturase |
| syn:slr0952 | fbp; fructose-1,6-bisphosphatase (EC:3.1.3.11) |
| syn:slr0969 | cbiH; precorrin methylase |
| syn:slr1030 | chlI; Mg chelatase subunit ChlI |
| syn:slr1055 | chlH; magnesium chelatase subunit H (EC:6.6.1.1) |
| syn:slr1067 | galE; UDP-glucose-4-epimerase |
| syn:slr1123 | gmk; guanylate kinase (EC:2.7.4.8) |
| syn:slr1124 | gpmB; phosphoglycerate mutase |
| syn:slr1136 | ctaC; cytochrome C oxidase subunit II |
| syn:slr1138 | ctaE; cytochrome C oxidase subunit III |
| syn:slr1164 | nrdA; ribonucleotide reductase subunit alpha |
| syn:slr1176 | glgC; glucose-1-phosphate adenylyltransferase (EC:2.7.7.27) |
| syn:slr1181 | psbA1; photosystem II D1 protein |
| syn:slr1237 | codA; cytosine deaminase (EC:3.5.4.1) |
| syn:slr1254 | pds; phytoene desaturase |
| syn:slr1265 | rpoC1; DNA-directed RNA polymerase subunit gamma (EC:2.7.7.6) |
| syn:slr1279 | ndhC; NADH dehydrogenase subunit A (EC:1.6.5.3) |
| syn:slr1280 | ndhK; NADH dehydrogenase subunit B (EC:1.6.5.3) |
| syn:slr1289 | icd; isocitrate dehydrogenase (EC:1.1.1.41) |
| syn:slr1299 | UDP-glucose dehydrogenase |
| syn:slr1300 | ubiH; 2-octaprenyl-6-methoxyphenyl hydroxylase |
| syn:slr1312 | speA; arginine decarboxylase (EC:4.1.1.19) |
| syn:slr1349 | pgi; glucose-6-phosphate isomerase (EC:5.3.1.9) |
| syn:slr1364 | bioB; biotin synthetase |
| syn:slr1367 | glgP; glycogen phosphorylase |
| syn:slr1418 | pyrD; dihydroorotate dehydrogenase 2 (EC:1.3.98.1) |
| syn:slr1423 | murC; UDP-N-acetylmuramate--L-alanine ligase (EC:6.3.2.8) |
| syn:slr1424 | murB; UDP-N-acetylenolpyruvoylglucosamine reductase |
| syn:slr1448 | cscK; fructokinase |
| syn:slr1467 | precorrin-8X methylmutase (EC:5.4.1.2) |
| syn:slr1511 | fabH; 3-oxoacyl-ACP synthase (EC:2.3.1.41) |
| syn:slr1560 | hisZ; ATP phosphoribosyltransferase |
| syn:slr1655 | psaL; photosystem I reaction center protein subunit XI |
| syn:slr1656 | murG; UDP-N-acetylglucosamine-N-acetylmuramyl-(pentape ptide)pyrophosphoryl-undecaprenol N-acetylglucosamine transferase |
| syn:slr1691 | nadE; NAD synthetase (EC:6.3.1.5) |
| syn:slr1722 | guaB; inosine 5-monophosphate dehydrogenase (EC:1.1.1.205) |
| syn:slr1737 | hypothetical protein |
| syn:slr1739 | photosystem II protein |
| syn:slr1746 | murI; glutamate racemase (EC:5.1.1.3) |
| syn:slr1748 | hypothetical protein |
| syn:slr1762 | hypothetical protein |
| syn:slr1777 | chlD; Mg chelatase subunit ChlD |
| syn:slr1834 | psaA; photosystem I P700 chlorophyll a apoprotein A1 |
| syn:slr1843 | zwf; glucose-6-phosphate 1-dehydrogenase (EC:1.1.1.49) |
| syn:slr1848 | hisD; histidinol dehydrogenase (EC:1.1.1.23) |
| syn:slr1867 | trpD; anthranilate phosphoribosyltransferase (EC:2.4.2.18) |
| syn:slr1878 | cpcE; phycocyanin alpha phycocyanobilin lyase CpcE |
| syn:slr1882 | ribF; bifunctional riboflavin kinase/FMN adenylyltransferase (EC:2.7.1.26 2.7.7.2) |
| syn:slr1923 | hypothetical protein |
| syn:slr1925 | cobD; cobalamin biosynthesis protein |
| syn:slr1933 | rfbC; dTDP-4-dehydrorhamnose 3,5-epimerase |
| syn:slr1934 | pyruvate dehydrogenase E1 component subunit alpha |
| syn:slr1938 | mtnA; methylthioribose-1-phosphate isomerase (EC:5.3.1.23) |
| syn:slr1945 | yibO; phosphoglyceromutase (EC:5.4.2.1) |
| syn:slr1993 | thl; acetyl CoA acetyltransferase |
| syn:slr2026 | folP; dihydropteroate pyrophosphorylase |
| syn:slr2082 | ctaD; cytochrome C oxidase subunit I |
| syn:slr2088 | ilvG; acetolactate synthase 3 catalytic subunit (EC:2.2.1.6) |
| syn:slr2094 | glpX; fructose 1,6-bisphosphatase II (EC:3.1.3.11) |
| syn:slr2132 | pta; phosphate acetyltransferase (EC:2.3.1.8) |
| syn:slr2136 | ispG; 4-hydroxy-3-methylbut-2-en-1-yl diphosphate synthase (EC:1.17.7.1) |
| syn:smr0001 | psbT; photosystem II reaction center protein T |
| syn:ssr1386 | ndhL; NADH dehydrogenase subunit NdhL |
| syn:ssr2831 | psaE; photosystem I reaction center subunit IV |
| **syn01110 Biosynthesis of secondary metabolites - *Synechocystis* sp. PCC 6803 (104)** | |
| syn:sll0017 | hemL; glutamate-1-semialdehyde aminotransferase (EC:5.4.3.8) |
| syn:sll0080 | argC; N-acetyl-gamma-glutamyl-phosphate reductase (EC:1.2.1.38) |
| syn:sll0158 | glgB; glycogen branching protein (EC:2.4.1.18) |
| syn:sll0166 | hemD; uroporphyrin-III synthase |
| syn:sll0179 | gltX; glutamyl-tRNA synthetase (EC:6.1.1.17) |
| syn:sll0207 | rfbA; glucose-1-phosphate thymidylyltransferase |
| syn:sll0220 | glmS; glucosamine--fructose-6-phosphate aminotransferase (EC:2.6.1.16) |
| syn:sll0244 | galE; UDP-glucose-4-epimerase |
| syn:sll0401 | gltA; citrate synthase (EC:2.3.3.5) |
| syn:sll0402 | aspC; aspartate aminotransferase (EC:2.6.1.1) |
| syn:sll0421 | purB; adenylosuccinate lyase (EC:4.3.2.2) |
| syn:sll0422 | asparaginase |
| syn:sll0469 | prsA; ribose-phosphate pyrophosphokinase (EC:2.7.6.1) |
| syn:sll0480 | L,L-diaminopimelate aminotransferase |
| syn:sll0504 | lysA; diaminopimelate decarboxylase |
| syn:sll0578 | purK; phosphoribosylaminoimidazole carboxylase ATPase subunit (EC:4.1.1.21) |
| syn:sll0587 | pykF; pyruvate kinase |
| syn:sll0593 | glk; glucokinase (EC:2.7.1.2) |
| syn:sll0603 | menD; 2-succinyl-5-enolpyruvyl-6-hydroxy-3-cyclohexene-1-carboxylate synthase (EC:2.2.1.9 4.1.1.71) |
| syn:sll0711 | 4-diphosphocytidyl-2-C-methyl-D-erythritol kinase (EC:2.7.1.148) |
| syn:sll0728 | accA; acetyl-CoA carboxylase carboxyltransferase subunit alpha (EC:6.4.1.2) |
| syn:sll0823 | sdhB; succinate dehydrogenase iron-sulfur subunit (EC:1.3.99.1) |
| syn:sll0892 | panD; aspartate alpha-decarboxylase (EC:4.1.1.11) |
| syn:sll0927 | metX; S-adenosylmethionine synthetase (EC:2.5.1.6) |
| syn:sll0934 | ccmA; 3-deoxy-7-phosphoheptulonate synthase |
| syn:sll1023 | sucC; succinate--CoA ligase |
| syn:sll1056 | purL; phosphoribosylformylglycinamidine synthase II (EC:6.3.5.3) |
| syn:sll1059 | adk; adenylate kinase |
| syn:sll1070 | tktA; transketolase (EC:2.2.1.1) |
| syn:sll1108 | surE; stationary phase survival protein SurE (EC:3.1.3.2) |
| syn:sll1214 | magnesium-protoporphyrin IX monomethyl ester cyclase |
| syn:sll1329 | monophosphatase |
| syn:sll1459 | surE; stationary phase survival protein SurE (EC:3.1.3.2) |
| syn:sll1470 | leuC; isopropylmalate isomerase large subunit (EC:4.2.1.33) |
| syn:sll1479 | devB; 6-phosphogluconolactonase |
| syn:sll1538 | bgl; beta-glucosidase |
| syn:sll1556 | isopentenyl pyrophosphate isomerase (EC:5.3.3.2) |
| syn:sll1557 | sucD; succinyl-CoA synthase subunit beta |
| syn:sll1625 | sdhB; succinate dehydrogenase iron-sulfur subunit (EC:1.3.99.1) |
| syn:sll1647 | hypothetical protein |
| syn:sll1721 | pdhB; Pyruvate dehydrogenase E1 component subunit beta |
| syn:sll1841 | odhB; branched-chain alpha-keto acid dehydrogenase E2 |
| syn:sll1883 | argJ; bifunctional ornithine acetyltransferase/N-acetylglutamate synthase (EC:2.3.1.1 2.3.1.35) |
| syn:sll1893 | hisF; imidazole glycerol phosphate synthase subunit HisF |
| syn:sll1899 | ctaB; protoheme IX farnesyltransferase |
| syn:sll1945 | 1-deoxy-D-xylulose-5-phosphate synthase (EC:2.2.1.7) |
| syn:sll1981 | ilvB; acetolactate synthase |
| syn:sll1994 | hemB; delta-aminolevulinic acid dehydratase (EC:4.2.1.24) |
| syn:slr0056 | chlG; bacteriochlorophyll/chlorophyll a synthase |
| syn:slr0084 | hisH; imidazole glycerol phosphate synthase subunit HisH |
| syn:slr0091 | aldehyde dehydrogenase |
| syn:slr0121 | hypothetical protein |
| syn:slr0204 | hypothetical protein |
| syn:slr0212 | metH; 5-methyltetrahydrofolate--homocysteine methyltransferase |
| syn:slr0237 | glgX; glycogen operon protein GlgX |
| syn:slr0452 | ilvD; dihydroxy-acid dehydratase (EC:4.2.1.9) |
| syn:slr0458 | hypothetical protein |
| syn:slr0500 | hisB; imidazoleglycerol-phosphate dehydratase (EC:4.2.1.19) |
| syn:slr0536 | hemE; uroporphyrinogen decarboxylase (EC:4.1.1.37) |
| syn:slr0546 | trpC; indole-3-glycerol phosphate synthase (EC:4.1.1.48) |
| syn:slr0549 | asd; aspartate beta-semialdehyde dehydrogenese |
| syn:slr0550 | dapA; dihydrodipicolinate synthase (EC:4.2.1.52) |
| syn:slr0585 | argG; argininosuccinate synthase (EC:6.3.4.5) |
| syn:slr0597 | purH; bifunctional phosphoribosylaminoimidazolecarboxamide formyltransferase/IMP cyclohydrolase (EC:2.1.2.3 3.5.4.10) |
| syn:slr0608 | hisI; bifunctional phosphoribosyl-AMP cyclohydrolase/phosphoribosyl-ATP (EC:3.5.4.19 3.6.1.31) |
| syn:slr0611 | sds; solanesyl diphosphate synthase |
| syn:slr0657 | lysC; aspartate kinase (EC:2.7.2.4) |
| syn:slr0665 | bifunctional aconitate hydratase 2/2-methylisocitrate dehydratase (EC:4.2.1.3) |
| syn:slr0682 | hisD; histidinol dehydrogenase |
| syn:slr0738 | trpE; anthranilate synthase component I |
| syn:slr0750 | chlN; light-independent protochlorophyllide reductase subunit N |
| syn:slr0836 | rfbB; dTDP-glucose 4,6-dehydratase |
| syn:slr0838 | purM; phosphoribosylaminoimidazole synthetase (EC:6.3.3.1) |
| syn:slr0940 | crtQ; zeta-carotene desaturase |
| syn:slr0952 | fbp; fructose-1,6-bisphosphatase (EC:3.1.3.11) |
| syn:slr0984 | rfbG; CDP-glucose-4,6-dehydratase |
| syn:slr1030 | chlI; Mg chelatase subunit ChlI |
| syn:slr1055 | chlH; magnesium chelatase subunit H (EC:6.6.1.1) |
| syn:slr1067 | galE; UDP-glucose-4-epimerase |
| syn:slr1124 | gpmB; phosphoglycerate mutase |
| syn:slr1176 | glgC; glucose-1-phosphate adenylyltransferase (EC:2.7.7.27) |
| syn:slr1254 | pds; phytoene desaturase |
| syn:slr1289 | icd; isocitrate dehydrogenase (EC:1.1.1.41) |
| syn:slr1299 | UDP-glucose dehydrogenase |
| syn:slr1300 | ubiH; 2-octaprenyl-6-methoxyphenyl hydroxylase |
| syn:slr1349 | pgi; glucose-6-phosphate isomerase (EC:5.3.1.9) |
| syn:slr1367 | glgP; glycogen phosphorylase |
| syn:slr1560 | hisZ; ATP phosphoribosyltransferase |
| syn:slr1722 | guaB; inosine 5-monophosphate dehydrogenase (EC:1.1.1.205) |
| syn:slr1737 | hypothetical protein |
| syn:slr1748 | hypothetical protein |
| syn:slr1762 | hypothetical protein |
| syn:slr1777 | chlD; Mg chelatase subunit ChlD |
| syn:slr1843 | zwf; glucose-6-phosphate 1-dehydrogenase (EC:1.1.1.49) |
| syn:slr1848 | hisD; histidinol dehydrogenase (EC:1.1.1.23) |
| syn:slr1867 | trpD; anthranilate phosphoribosyltransferase (EC:2.4.2.18) |
| syn:slr1933 | rfbC; dTDP-4-dehydrorhamnose 3,5-epimerase |
| syn:slr1934 | pyruvate dehydrogenase E1 component subunit alpha |
| syn:slr1945 | yibO; phosphoglyceromutase (EC:5.4.2.1) |
| syn:slr1993 | thl; acetyl CoA acetyltransferase |
| syn:slr2088 | ilvG; acetolactate synthase 3 catalytic subunit (EC:2.2.1.6) |
| syn:slr2089 | shc; squalene-hopene cyclase |
| syn:slr2094 | glpX; fructose 1,6-bisphosphatase II (EC:3.1.3.11) |
| syn:slr2136 | ispG; 4-hydroxy-3-methylbut-2-en-1-yl diphosphate synthase (EC:1.17.7.1) |
| **syn01120 Microbial metabolism in diverse environments - *Synechocystis* sp. PCC 6803 (45)** | |
| syn:sll0401 | gltA; citrate synthase (EC:2.3.3.5) |
| syn:sll0402 | aspC; aspartate aminotransferase (EC:2.6.1.1) |
| syn:sll0450 | norB; cytochrome B subunit of nitric oxide reductase |
| syn:sll0469 | prsA; ribose-phosphate pyrophosphokinase (EC:2.7.6.1) |
| syn:sll0504 | lysA; diaminopimelate decarboxylase |
| syn:sll0587 | pykF; pyruvate kinase |
| syn:sll0593 | glk; glucokinase (EC:2.7.1.2) |
| syn:sll0712 | cysM; cysteine synthase A |
| syn:sll0728 | accA; acetyl-CoA carboxylase carboxyltransferase subunit alpha (EC:6.4.1.2) |
| syn:sll0823 | sdhB; succinate dehydrogenase iron-sulfur subunit (EC:1.3.99.1) |
| syn:sll0895 | cysQ; ammonium transporter |
| syn:sll1023 | sucC; succinate--CoA ligase |
| syn:sll1070 | tktA; transketolase (EC:2.2.1.1) |
| syn:sll1172 | thrC; threonine synthase (EC:4.2.3.1) |
| syn:sll1454 | narB; nitrate reductase |
| syn:sll1479 | devB; 6-phosphogluconolactonase |
| syn:sll1557 | sucD; succinyl-CoA synthase subunit beta |
| syn:sll1625 | sdhB; succinate dehydrogenase iron-sulfur subunit (EC:1.3.99.1) |
| syn:sll1709 | gdh; glucose dehydrogenase |
| syn:sll1721 | pdhB; Pyruvate dehydrogenase E1 component subunit beta |
| syn:sll1750 | ureC; urease subunit alpha (EC:3.5.1.5) |
| syn:sll1760 | thrB; homoserine kinase (EC:2.7.1.39) |
| syn:sll1841 | odhB; branched-chain alpha-keto acid dehydrogenase E2 |
| syn:sll1908 | serA; D-3-phosphoglycerate dehydrogenase |
| syn:sll1987 | katG; catalase HPI |
| syn:slr0009 | rbcL; ribulose bisophosphate carboxylase (EC:4.1.1.39) |
| syn:slr0091 | aldehyde dehydrogenase |
| syn:slr0301 | ppsA; phosphoenolpyruvate synthase (EC:2.7.9.2) |
| syn:slr0549 | asd; aspartate beta-semialdehyde dehydrogenese |
| syn:slr0550 | dapA; dihydrodipicolinate synthase (EC:4.2.1.52) |
| syn:slr0657 | lysC; aspartate kinase (EC:2.7.2.4) |
| syn:slr0665 | bifunctional aconitate hydratase 2/2-methylisocitrate dehydratase (EC:4.2.1.3) |
| syn:slr0952 | fbp; fructose-1,6-bisphosphatase (EC:3.1.3.11) |
| syn:slr1124 | gpmB; phosphoglycerate mutase |
| syn:slr1289 | icd; isocitrate dehydrogenase (EC:1.1.1.41) |
| syn:slr1349 | pgi; glucose-6-phosphate isomerase (EC:5.3.1.9) |
| syn:slr1718 | 2-phosphosulfolactate phosphatase (EC:3.1.3.71) |
| syn:slr1748 | hypothetical protein |
| syn:slr1843 | zwf; glucose-6-phosphate 1-dehydrogenase (EC:1.1.1.49) |
| syn:slr1923 | hypothetical protein |
| syn:slr1934 | pyruvate dehydrogenase E1 component subunit alpha |
| syn:slr1945 | yibO; phosphoglyceromutase (EC:5.4.2.1) |
| syn:slr1993 | thl; acetyl CoA acetyltransferase |
| syn:slr2094 | glpX; fructose 1,6-bisphosphatase II (EC:3.1.3.11) |
| syn:slr2132 | pta; phosphate acetyltransferase (EC:2.3.1.8) |
| **syn02020 Two-component system - *Synechocystis* sp. PCC 6803 (28)** | |
| syn:sll0038 | PatA subfamily protein |
| syn:sll0540 | hypothetical protein |
| syn:sll0680 | pstS; phosphate-binding periplasmic protein |
| syn:sll0698 | dfr; drug sensory protein A |
| syn:sll0779 | PleD protein |
| syn:sll0797 | OmpR subfamily protein |
| syn:sll0798 | sensory transduction histidine kinase |
| syn:sll0848 | dnaA; chromosome replication initiator DnaA |
| syn:sll1293 | hypothetical protein |
| syn:sll1294 | pilJ; methyl-accepting chemotaxis-like-protein |
| syn:sll1296 | chemotaxis protein CheA |
| syn:sll1598 | mntC; Mn transporter MntC |
| syn:sll1600 | mntB; Mn transporter MntB |
| syn:slr0115 | OmpR subfamily protein |
| syn:slr0121 | hypothetical protein |
| syn:slr0640 | sensory transduction histidine kinase |
| syn:slr0687 | pleD; PleD protein |
| syn:slr0721 | me; malic enzyme |
| syn:slr1041 | PatA subfamily protein |
| syn:slr1214 | PatA subfamily protein |
| syn:slr1247 | pstS; phosphate binding protein |
| syn:slr1350 | desA; fatty acid desaturase |
| syn:slr1728 | kdpA; potassium-transporting ATPase subunit A (EC:3.6.3.12) |
| syn:slr1729 | kdpB; potassium-transporting ATPase subunit B (EC:3.6.3.12) |
| syn:slr1798 | pleD; PleD protein |
| syn:slr1837 | OmpR subfamily protein |
| syn:slr1969 | hybrid sensory kinase |
| syn:slr1993 | thl; acetyl CoA acetyltransferase |
| **syn02010 ABC transporters - *Synechocystis* sp. PCC 6803 (27)** | |
| syn:sll0182 | ABC transporter |
| syn:sll0224 | hypothetical protein |
| syn:sll0374 | braG; high-affinity branched-chain amino acid transport ATP-binding protein |
| syn:sll0385 | ABC transporter |
| syn:sll0489 | ABC transporter |
| syn:sll0540 | hypothetical protein |
| syn:sll0606 | hypothetical protein |
| syn:sll0680 | pstS; phosphate-binding periplasmic protein |
| syn:sll1202 | hypothetical protein |
| syn:sll1451 | nrtB; nitrate transport protein NrtB |
| syn:sll1598 | mntC; Mn transporter MntC |
| syn:sll1600 | mntB; Mn transporter MntB |
| syn:sll1878 | ABC transporter |
| syn:slr0040 | cmpA; bicarbonate transporter |
| syn:slr0327 | hitB; iron utilization protein |
| syn:slr0467 | natA; neutral amino acids ABC transporter protein |
| syn:slr0513 | periplasmic iron-binding protein |
| syn:slr0559 | natB; neutral amino acids ABC transporter substrate-binding protein |
| syn:slr1201 | hypothetical protein |
| syn:slr1247 | pstS; phosphate binding protein |
| syn:slr1249 | pstA; phosphate ABC transporter permease |
| syn:slr1319 | fecB; iron(III) dicitrate ABC transporter permease |
| syn:slr1453 | cysT; sulfate ABC transporter permease |
| syn:slr1488 | ABC transporter |
| syn:slr1491 | fecB; iron(III) dicitrate-binding periplasmic protein |
| syn:slr1492 | fecB; iron(III) dicitrate-binding periplasmic protein |
| syn:slr1494 | ABC transporter |
| **syn00230 Purine metabolism - *Synechocystis* sp. PCC 6803 (25)** | |
| syn:sll0398 | dgt; dGTP triphosphohydrolase |
| syn:sll0421 | purB; adenylosuccinate lyase (EC:4.3.2.2) |
| syn:sll0469 | prsA; ribose-phosphate pyrophosphokinase (EC:2.7.6.1) |
| syn:sll0544 | DNA polymerase III subunit delta |
| syn:sll0578 | purK; phosphoribosylaminoimidazole carboxylase ATPase subunit (EC:4.1.1.21) |
| syn:sll0587 | pykF; pyruvate kinase |
| syn:sll1043 | pnp; polynucleotide phosphorylase |
| syn:sll1056 | purL; phosphoribosylformylglycinamidine synthase II (EC:6.3.5.3) |
| syn:sll1059 | adk; adenylate kinase |
| syn:sll1108 | surE; stationary phase survival protein SurE (EC:3.1.3.2) |
| syn:sll1360 | dnaX; DNA polymerase III subunit |
| syn:sll1459 | surE; stationary phase survival protein SurE (EC:3.1.3.2) |
| syn:sll1546 | ppx; exopolyphosphatase |
| syn:sll1750 | ureC; urease subunit alpha (EC:3.5.1.5) |
| syn:sll1787 | rpoB; DNA-directed RNA polymerase subunit beta (EC:2.7.7.6) |
| syn:sll1818 | rpoA; DNA-directed RNA polymerase subunit alpha (EC:2.7.7.6) |
| syn:slr0402 | deoxyribonucleotide triphosphate pyrophosphatase |
| syn:slr0597 | purH; bifunctional phosphoribosylaminoimidazolecarboxamide formyltransferase/IMP cyclohydrolase (EC:2.1.2.3 3.5.4.10) |
| syn:slr0603 | dnaE; DNA polymerase III subunit alpha |
| syn:slr0838 | purM; phosphoribosylaminoimidazole synthetase (EC:6.3.3.1) |
| syn:slr1123 | gmk; guanylate kinase (EC:2.7.4.8) |
| syn:slr1134 | hypothetical protein |
| syn:slr1164 | nrdA; ribonucleotide reductase subunit alpha |
| syn:slr1265 | rpoC1; DNA-directed RNA polymerase subunit gamma (EC:2.7.7.6) |
| syn:slr1722 | guaB; inosine 5-monophosphate dehydrogenase (EC:1.1.1.205) |
| **syn00240 Pyrimidine metabolism - *Synechocystis* sp. PCC 6803 (19)** | |
| syn:sll0144 | pyrH; uridylate kinase |
| syn:sll0370 | carB; carbamoyl phosphate synthase large subunit (EC:6.3.5.5) |
| syn:sll0544 | DNA polymerase III subunit delta |
| syn:sll0838 | pyrF; orotidine 5'-phosphate decarboxylase (EC:4.1.1.23) |
| syn:sll1018 | pyrC; dihydroorotase (EC:3.5.2.3) |
| syn:sll1043 | pnp; polynucleotide phosphorylase |
| syn:sll1108 | surE; stationary phase survival protein SurE (EC:3.1.3.2) |
| syn:sll1360 | dnaX; DNA polymerase III subunit |
| syn:sll1443 | pyrG; CTP synthetase (EC:6.3.4.2) |
| syn:sll1459 | surE; stationary phase survival protein SurE (EC:3.1.3.2) |
| syn:sll1498 | carA; carbamoyl phosphate synthase small subunit (EC:6.3.5.5) |
| syn:sll1787 | rpoB; DNA-directed RNA polymerase subunit beta (EC:2.7.7.6) |
| syn:sll1818 | rpoA; DNA-directed RNA polymerase subunit alpha (EC:2.7.7.6) |
| syn:slr0402 | deoxyribonucleotide triphosphate pyrophosphatase |
| syn:slr0603 | dnaE; DNA polymerase III subunit alpha |
| syn:slr1164 | nrdA; ribonucleotide reductase subunit alpha |
| syn:slr1237 | codA; cytosine deaminase (EC:3.5.4.1) |
| syn:slr1265 | rpoC1; DNA-directed RNA polymerase subunit gamma (EC:2.7.7.6) |
| syn:slr1418 | pyrD; dihydroorotate dehydrogenase 2 (EC:1.3.98.1) |
| **syn00860 Porphyrin and chlorophyll metabolism - *Synechocystis* sp. PCC 6803 (18)** | |
| syn:sll0017 | hemL; glutamate-1-semialdehyde aminotransferase (EC:5.4.3.8) |
| syn:sll0166 | hemD; uroporphyrin-III synthase |
| syn:sll0179 | gltX; glutamyl-tRNA synthetase (EC:6.1.1.17) |
| syn:sll0794 | merR; mercuric resistance operon regulatory protein precorrin isomerase |
| syn:sll1184 | ho1; heme oxygenase |
| syn:sll1214 | magnesium-protoporphyrin IX monomethyl ester cyclase |
| syn:sll1899 | ctaB; protoheme IX farnesyltransferase |
| syn:sll1994 | hemB; delta-aminolevulinic acid dehydratase (EC:4.2.1.24) |
| syn:slr0056 | chlG; bacteriochlorophyll/chlorophyll a synthase |
| syn:slr0252 | cobK; cobalt-precorrin-6x reductase (EC:1.3.1.54) |
| syn:slr0536 | hemE; uroporphyrinogen decarboxylase (EC:4.1.1.37) |
| syn:slr0750 | chlN; light-independent protochlorophyllide reductase subunit N |
| syn:slr0969 | cbiH; precorrin methylase |
| syn:slr1030 | chlI; Mg chelatase subunit ChlI |
| syn:slr1055 | chlH; magnesium chelatase subunit H (EC:6.6.1.1) |
| syn:slr1467 | precorrin-8X methylmutase (EC:5.4.1.2) |
| syn:slr1777 | chlD; Mg chelatase subunit ChlD |
| syn:slr1925 | cobD; cobalamin biosynthesis protein |
| **syn00190 Oxidative phosphorylation - *Synechocystis* sp. PCC 6803 (18)** | |
| syn:sll0290 | ppk; polyphosphate kinase (EC:2.7.4.1) |
| syn:sll0519 | ndhA; NADH dehydrogenase subunit H (EC:1.6.5.3) |
| syn:sll0823 | sdhB; succinate dehydrogenase iron-sulfur subunit (EC:1.3.99.1) |
| syn:sll1220 | bidirectional hydrogenase complex protein HoxE |
| syn:sll1484 | ndh; NADH dehydrogenase |
| syn:sll1625 | sdhB; succinate dehydrogenase iron-sulfur subunit (EC:1.3.99.1) |
| syn:sll1732 | ndhF; NAD(P)H-quinone oxidoreductase subunit F |
| syn:sll1733 | ndhD3; NAD(P)H-quinone oxidoreductase subunit M |
| syn:sll1899 | ctaB; protoheme IX farnesyltransferase |
| syn:slr0261 | ndhH; NAD(P)H-quinone oxidoreductase subunit H |
| syn:slr0844 | ndhF; NAD(P)H-quinone oxidoreductase subunit F |
| syn:slr0851 | ndh; NADH dehydrogenase |
| syn:slr1136 | ctaC; cytochrome C oxidase subunit II |
| syn:slr1138 | ctaE; cytochrome C oxidase subunit III |
| syn:slr1279 | ndhC; NADH dehydrogenase subunit A (EC:1.6.5.3) |
| syn:slr1280 | ndhK; NADH dehydrogenase subunit B (EC:1.6.5.3) |
| syn:slr2082 | ctaD; cytochrome C oxidase subunit I |
| syn:ssr1386 | ndhL; NADH dehydrogenase subunit NdhL |
| **syn00520 Amino sugar and nucleotide sugar metabolism - *Synechocystis* sp. PCC 6803 (13)** | |
| syn:sll0220 | glmS; glucosamine--fructose-6-phosphate aminotransferase (EC:2.6.1.16) |
| syn:sll0244 | galE; UDP-glucose-4-epimerase |
| syn:sll0593 | glk; glucokinase (EC:2.7.1.2) |
| syn:sll0861 | murQ; N-acetylmuramic acid 6-phosphate etherase |
| syn:sll1496 | mannose-1-phosphate guanylyltransferase |
| syn:slr0017 | murZ; UDP-N-acetylglucosamine 1-carboxyvinyltransferase (EC:2.5.1.7) |
| syn:slr0984 | rfbG; CDP-glucose-4,6-dehydratase |
| syn:slr1067 | galE; UDP-glucose-4-epimerase |
| syn:slr1176 | glgC; glucose-1-phosphate adenylyltransferase (EC:2.7.7.27) |
| syn:slr1299 | UDP-glucose dehydrogenase |
| syn:slr1349 | pgi; glucose-6-phosphate isomerase (EC:5.3.1.9) |
| syn:slr1424 | murB; UDP-N-acetylenolpyruvoylglucosamine reductase |
| syn:slr1448 | cscK; fructokinase |
| **syn00620 Pyruvate metabolism - *Synechocystis* sp. PCC 6803 (12)** | |
| syn:sll0587 | pykF; pyruvate kinase |
| syn:sll0728 | accA; acetyl-CoA carboxylase carboxyltransferase subunit alpha (EC:6.4.1.2) |
| syn:sll1721 | pdhB; Pyruvate dehydrogenase E1 component subunit beta |
| syn:sll1841 | odhB; branched-chain alpha-keto acid dehydrogenase E2 |
| syn:slr0091 | aldehyde dehydrogenase |
| syn:slr0301 | ppsA; phosphoenolpyruvate synthase (EC:2.7.9.2) |
| syn:slr0721 | me; malic enzyme |
| syn:slr1259 | hypothetical protein |
| syn:slr1556 | ddh; D-lactate dehydrogenase |
| syn:slr1934 | pyruvate dehydrogenase E1 component subunit alpha |
| syn:slr1993 | thl; acetyl CoA acetyltransferase |
| syn:slr2132 | pta; phosphate acetyltransferase (EC:2.3.1.8) |
| **syn01210 2-Oxocarboxylic acid metabolism - *Synechocystis* sp. PCC 6803 (12)** | |
| syn:sll0080 | argC; N-acetyl-gamma-glutamyl-phosphate reductase (EC:1.2.1.38) |
| syn:sll0401 | gltA; citrate synthase (EC:2.3.3.5) |
| syn:sll0402 | aspC; aspartate aminotransferase (EC:2.6.1.1) |
| syn:sll1470 | leuC; isopropylmalate isomerase large subunit (EC:4.2.1.33) |
| syn:sll1883 | argJ; bifunctional ornithine acetyltransferase/N-acetylglutamate synthase (EC:2.3.1.1 2.3.1.35) |
| syn:sll1981 | ilvB; acetolactate synthase |
| syn:slr0452 | ilvD; dihydroxy-acid dehydratase (EC:4.2.1.9) |
| syn:slr0549 | asd; aspartate beta-semialdehyde dehydrogenese |
| syn:slr0657 | lysC; aspartate kinase (EC:2.7.2.4) |
| syn:slr0665 | bifunctional aconitate hydratase 2/2-methylisocitrate dehydratase (EC:4.2.1.3) |
| syn:slr1289 | icd; isocitrate dehydrogenase (EC:1.1.1.41) |
| syn:slr2088 | ilvG; acetolactate synthase 3 catalytic subunit (EC:2.2.1.6) |
| **syn00970 Aminoacyl-tRNA biosynthesis - *Synechocystis* sp. PCC 6803 (12)** | |
| syn:ST6803t08 | tRNA-Arg |
| syn:sll0078 | thrS; threonyl-tRNA synthetase (EC:6.1.1.3) |
| syn:sll0179 | gltX; glutamyl-tRNA synthetase (EC:6.1.1.17) |
| syn:sll1362 | ileS; isoleucyl-tRNA synthetase (EC:6.1.1.5) |
| syn:sll1425 | proS; prolyl-tRNA synthetase (EC:6.1.1.15) |
| syn:sll1553 | pheT; phenylalanyl-tRNA synthetase subunit beta (EC:6.1.1.20) |
| syn:slr0357 | hisS; histidyl-tRNA synthetase (EC:6.1.1.21) |
| syn:slr0557 | valS; valyl-tRNA synthetase (EC:6.1.1.9) |
| syn:slr0649 | metG; methionyl-tRNA synthetase (EC:6.1.1.10) |
| syn:slr0958 | cysS; cysteinyl-tRNA synthetase (EC:6.1.1.16) |
| syn:slr1031 | tyrS; tyrosyl-tRNA synthetase (EC:6.1.1.1) |
| syn:slr1703 | serS; seryl-tRNA synthetase (EC:6.1.1.11) |
| **syn00010 Glycolysis / Gluconeogenesis - *Synechocystis* sp. PCC 6803 (12)** | |
| syn:sll0587 | pykF; pyruvate kinase |
| syn:sll0593 | glk; glucokinase (EC:2.7.1.2) |
| syn:sll1721 | pdhB; Pyruvate dehydrogenase E1 component subunit beta |
| syn:sll1841 | odhB; branched-chain alpha-keto acid dehydrogenase E2 |
| syn:slr0091 | aldehyde dehydrogenase |
| syn:slr0952 | fbp; fructose-1,6-bisphosphatase (EC:3.1.3.11) |
| syn:slr1124 | gpmB; phosphoglycerate mutase |
| syn:slr1349 | pgi; glucose-6-phosphate isomerase (EC:5.3.1.9) |
| syn:slr1748 | hypothetical protein |
| syn:slr1934 | pyruvate dehydrogenase E1 component subunit alpha |
| syn:slr1945 | yibO; phosphoglyceromutase (EC:5.4.2.1) |
| syn:slr2094 | glpX; fructose 1,6-bisphosphatase II (EC:3.1.3.11) |
| **syn00680 Methane metabolism - *Synechocystis* sp. PCC 6803 (11)** | |
| syn:sll1908 | serA; D-3-phosphoglycerate dehydrogenase |
| syn:sll1987 | katG; catalase HPI |
| syn:slr0301 | ppsA; phosphoenolpyruvate synthase (EC:2.7.9.2) |
| syn:slr0952 | fbp; fructose-1,6-bisphosphatase (EC:3.1.3.11) |
| syn:slr1124 | gpmB; phosphoglycerate mutase |
| syn:slr1718 | 2-phosphosulfolactate phosphatase (EC:3.1.3.71) |
| syn:slr1748 | hypothetical protein |
| syn:slr1923 | hypothetical protein |
| syn:slr1945 | yibO; phosphoglyceromutase (EC:5.4.2.1) |
| syn:slr2094 | glpX; fructose 1,6-bisphosphatase II (EC:3.1.3.11) |
| syn:slr2132 | pta; phosphate acetyltransferase (EC:2.3.1.8) |
| **syn00330 Arginine and proline metabolism - *Synechocystis* sp. PCC 6803 (11)** | |
| syn:sll0080 | argC; N-acetyl-gamma-glutamyl-phosphate reductase (EC:1.2.1.38) |
| syn:sll0402 | aspC; aspartate aminotransferase (EC:2.6.1.1) |
| syn:sll0461 | proA; gamma-glutamyl phosphate reductase |
| syn:sll1077 | speB; agmatine ureohydrolase |
| syn:sll1750 | ureC; urease subunit alpha (EC:3.5.1.5) |
| syn:sll1883 | argJ; bifunctional ornithine acetyltransferase/N-acetylglutamate synthase (EC:2.3.1.1 2.3.1.35) |
| syn:slr0091 | aldehyde dehydrogenase |
| syn:slr0585 | argG; argininosuccinate synthase (EC:6.3.4.5) |
| syn:slr0662 | speA; arginine decarboxylase (EC:4.1.1.19) |
| syn:slr1237 | codA; cytosine deaminase (EC:3.5.4.1) |
| syn:slr1312 | speA; arginine decarboxylase (EC:4.1.1.19) |
| **syn00020 Citrate cycle (TCA cycle) - *Synechocystis* sp. PCC 6803 (10)** | |
| syn:sll0401 | gltA; citrate synthase (EC:2.3.3.5) |
| syn:sll0823 | sdhB; succinate dehydrogenase iron-sulfur subunit (EC:1.3.99.1) |
| syn:sll1023 | sucC; succinate--CoA ligase |
| syn:sll1557 | sucD; succinyl-CoA synthase subunit beta |
| syn:sll1625 | sdhB; succinate dehydrogenase iron-sulfur subunit (EC:1.3.99.1) |
| syn:sll1721 | pdhB; Pyruvate dehydrogenase E1 component subunit beta |
| syn:sll1841 | odhB; branched-chain alpha-keto acid dehydrogenase E2 |
| syn:slr0665 | bifunctional aconitate hydratase 2/2-methylisocitrate dehydratase (EC:4.2.1.3) |
| syn:slr1289 | icd; isocitrate dehydrogenase (EC:1.1.1.41) |
| syn:slr1934 | pyruvate dehydrogenase E1 component subunit alpha |
| **syn00910 Nitrogen metabolism - *Synechocystis* sp. PCC 6803 (10)** | |
| syn:sll0422 | asparaginase |
| syn:sll0450 | norB; cytochrome B subunit of nitric oxide reductase |
| syn:sll1451 | nrtB; nitrate transport protein NrtB |
| syn:sll1454 | narB; nitrate reductase |
| syn:sll1499 | gltB; ferredoxin-dependent glutamate synthase |
| syn:sll1502 | gltB; glutamate synthase |
| syn:slr0051 | icfA; carbonic anhydrase |
| syn:slr1136 | ctaC; cytochrome C oxidase subunit II |
| syn:slr1138 | ctaE; cytochrome C oxidase subunit III |
| syn:slr2082 | ctaD; cytochrome C oxidase subunit I |
| **syn00500 Starch and sucrose metabolism - *Synechocystis* sp. PCC 6803 (10)** | |
| syn:sll0158 | glgB; glycogen branching protein (EC:2.4.1.18) |
| syn:sll0593 | glk; glucokinase (EC:2.7.1.2) |
| syn:sll1538 | bgl; beta-glucosidase |
| syn:slr0237 | glgX; glycogen operon protein GlgX |
| syn:slr0897 | endo-1,4-beta-glucanase |
| syn:slr1176 | glgC; glucose-1-phosphate adenylyltransferase (EC:2.7.7.27) |
| syn:slr1299 | UDP-glucose dehydrogenase |
| syn:slr1349 | pgi; glucose-6-phosphate isomerase (EC:5.3.1.9) |
| syn:slr1367 | glgP; glycogen phosphorylase |
| syn:slr1448 | cscK; fructokinase |
| **syn00195 Photosynthesis - *Synechocystis* sp. PCC 6803 (9)** | |
| syn:sll0427 | psbO; photosystem II manganese-stabilizing polypeptide |
| syn:sll0629 | psaK; photosystem I subunit X |
| syn:sll1317 | petA; apocytochrome f |
| syn:slr1181 | psbA1; photosystem II D1 protein |
| syn:slr1655 | psaL; photosystem I reaction center protein subunit XI |
| syn:slr1739 | photosystem II protein |
| syn:slr1834 | psaA; photosystem I P700 chlorophyll a apoprotein A1 |
| syn:smr0001 | psbT; photosystem II reaction center protein T |
| syn:ssr2831 | psaE; photosystem I reaction center subunit IV |
| **syn00250 Alanine, aspartate and glutamate metabolism - *Synechocystis* sp. PCC 6803 (9)** | |
| syn:sll0220 | glmS; glucosamine--fructose-6-phosphate aminotransferase (EC:2.6.1.16) |
| syn:sll0370 | carB; carbamoyl phosphate synthase large subunit (EC:6.3.5.5) |
| syn:sll0402 | aspC; aspartate aminotransferase (EC:2.6.1.1) |
| syn:sll0421 | purB; adenylosuccinate lyase (EC:4.3.2.2) |
| syn:sll0422 | asparaginase |
| syn:sll0631 | nadB; L-aspartate oxidase (EC:1.4.3.16) |
| syn:sll1498 | carA; carbamoyl phosphate synthase small subunit (EC:6.3.5.5) |
| syn:slr0585 | argG; argininosuccinate synthase (EC:6.3.4.5) |
| syn:slr1705 | aspA; aspartoacylase (EC:3.5.1.15) |
| **syn00270 Cysteine and methionine metabolism - *Synechocystis* sp. PCC 6803 (9)** | |
| syn:sll0135 | 5'-methylthioadenosine phosphorylase (EC:2.4.2.28) |
| syn:sll0402 | aspC; aspartate aminotransferase (EC:2.6.1.1) |
| syn:sll0712 | cysM; cysteine synthase A |
| syn:sll0927 | metX; S-adenosylmethionine synthetase (EC:2.5.1.6) |
| syn:sll1234 | ahcY; S-adenosyl-L-homocysteine hydrolase (EC:3.3.1.1) |
| syn:slr0212 | metH; 5-methyltetrahydrofolate--homocysteine methyltransferase |
| syn:slr0549 | asd; aspartate beta-semialdehyde dehydrogenese |
| syn:slr0657 | lysC; aspartate kinase (EC:2.7.2.4) |
| syn:slr1938 | mtnA; methylthioribose-1-phosphate isomerase (EC:5.3.1.23) |
| **syn00760 Nicotinate and nicotinamide metabolism - *Synechocystis* sp. PCC 6803 (9)** | |
| syn:sll0622 | nadA; quinolinate synthetase |
| syn:sll0631 | nadB; L-aspartate oxidase (EC:1.4.3.16) |
| syn:sll1108 | surE; stationary phase survival protein SurE (EC:3.1.3.2) |
| syn:sll1415 | ppnK; inorganic polyphosphate/ATP-NAD kinase (EC:2.7.1.23) |
| syn:sll1459 | surE; stationary phase survival protein SurE (EC:3.1.3.2) |
| syn:slr0787 | bifunctional nicotinamide mononucleotide adenylyltransferase/ADP-ribose pyrophosphatase (EC:2.7.7.1) |
| syn:slr0788 | nicotinate phosphoribosyltransferase |
| syn:slr0936 | nadC; nicotinate-nucleotide pyrophosphorylase (EC:2.4.2.19) |
| syn:slr1691 | nadE; NAD synthetase (EC:6.3.1.5) |
| **syn00340 Histidine metabolism - *Synechocystis* sp. PCC 6803 (9)** | |
| syn:sll1893 | hisF; imidazole glycerol phosphate synthase subunit HisF |
| syn:slr0084 | hisH; imidazole glycerol phosphate synthase subunit HisH |
| syn:slr0091 | aldehyde dehydrogenase |
| syn:slr0500 | hisB; imidazoleglycerol-phosphate dehydratase (EC:4.2.1.19) |
| syn:slr0608 | hisI; bifunctional phosphoribosyl-AMP cyclohydrolase/phosphoribosyl-ATP (EC:3.5.4.19 3.6.1.31) |
| syn:slr0682 | hisD; histidinol dehydrogenase |
| syn:slr1560 | hisZ; ATP phosphoribosyltransferase |
| syn:slr1705 | aspA; aspartoacylase (EC:3.5.1.15) |
| syn:slr1848 | hisD; histidinol dehydrogenase (EC:1.1.1.23) |
| **syn03440 Homologous recombination - *Synechocystis* sp. PCC 6803 (8)** | |
| syn:sll0544 | DNA polymerase III subunit delta |
| syn:sll0569 | recA; recombinase A |
| syn:sll1354 | recJ; ssDNA-specific exonuclease RecJ |
| syn:sll1360 | dnaX; DNA polymerase III subunit |
| syn:sll7106 | exodeoxyribonuclease V alpha chain |
| syn:slr0020 | recG; ATP-dependent DNA helicase RecG |
| syn:slr0181 | recO; DNA repair protein RecO |
| syn:slr0603 | dnaE; DNA polymerase III subunit alpha |
| **syn00630 Glyoxylate and dicarboxylate metabolism - *Synechocystis* sp. PCC 6803 (8)** | |
| syn:sll0401 | gltA; citrate synthase (EC:2.3.3.5) |
| syn:sll1499 | gltB; ferredoxin-dependent glutamate synthase |
| syn:sll1502 | gltB; glutamate synthase |
| syn:slr0009 | rbcL; ribulose bisophosphate carboxylase (EC:4.1.1.39) |
| syn:slr0458 | hypothetical protein |
| syn:slr0665 | bifunctional aconitate hydratase 2/2-methylisocitrate dehydratase (EC:4.2.1.3) |
| syn:slr1762 | hypothetical protein |
| syn:slr1993 | thl; acetyl CoA acetyltransferase |
| **syn00260 Glycine, serine and threonine metabolism - *Synechocystis* sp. PCC 6803 (8)** | |
| syn:sll1172 | thrC; threonine synthase (EC:4.2.3.1) |
| syn:sll1760 | thrB; homoserine kinase (EC:2.7.1.39) |
| syn:sll1908 | serA; D-3-phosphoglycerate dehydrogenase |
| syn:slr0549 | asd; aspartate beta-semialdehyde dehydrogenese |
| syn:slr0657 | lysC; aspartate kinase (EC:2.7.2.4) |
| syn:slr1124 | gpmB; phosphoglycerate mutase |
| syn:slr1748 | hypothetical protein |
| syn:slr1945 | yibO; phosphoglyceromutase (EC:5.4.2.1) |
| **syn00030 Pentose phosphate pathway - *Synechocystis* sp. PCC 6803 (8)** | |
| syn:sll0469 | prsA; ribose-phosphate pyrophosphokinase (EC:2.7.6.1) |
| syn:sll1070 | tktA; transketolase (EC:2.2.1.1) |
| syn:sll1479 | devB; 6-phosphogluconolactonase |
| syn:sll1709 | gdh; glucose dehydrogenase |
| syn:slr0952 | fbp; fructose-1,6-bisphosphatase (EC:3.1.3.11) |
| syn:slr1349 | pgi; glucose-6-phosphate isomerase (EC:5.3.1.9) |
| syn:slr1843 | zwf; glucose-6-phosphate 1-dehydrogenase (EC:1.1.1.49) |
| syn:slr2094 | glpX; fructose 1,6-bisphosphatase II (EC:3.1.3.11) |
| **syn00640 Propanoate metabolism - *Synechocystis* sp. PCC 6803 (7)** | |
| syn:sll0728 | accA; acetyl-CoA carboxylase carboxyltransferase subunit alpha (EC:6.4.1.2) |
| syn:sll1023 | sucC; succinate--CoA ligase |
| syn:sll1557 | sucD; succinyl-CoA synthase subunit beta |
| syn:slr0091 | aldehyde dehydrogenase |
| syn:slr0665 | bifunctional aconitate hydratase 2/2-methylisocitrate dehydratase (EC:4.2.1.3) |
| syn:slr1993 | thl; acetyl CoA acetyltransferase |
| syn:slr2132 | pta; phosphate acetyltransferase (EC:2.3.1.8) |
| **syn00480 Glutathione metabolism - *Synechocystis* sp. PCC 6803 (7)** | |
| syn:sll1147 | glutathione S-transferase |
| syn:sll1343 | ape2; aminopeptidase |
| syn:sll1902 | hypothetical protein |
| syn:sll2001 | lap; leucyl aminopeptidase (EC:3.4.11.1) |
| syn:slr1289 | icd; isocitrate dehydrogenase (EC:1.1.1.41) |
| syn:slr1843 | zwf; glucose-6-phosphate 1-dehydrogenase (EC:1.1.1.49) |
| syn:slr1992 | glutathione peroxidase |
| **syn03430 Mismatch repair - *Synechocystis* sp. PCC 6803 (7)** | |
| syn:sll0544 | DNA polymerase III subunit delta |
| syn:sll1143 | uvrD; DNA helicase II |
| syn:sll1165 | mutS; DNA mismatch repair protein MutS |
| syn:sll1354 | recJ; ssDNA-specific exonuclease RecJ |
| syn:sll1360 | dnaX; DNA polymerase III subunit |
| syn:slr0603 | dnaE; DNA polymerase III subunit alpha |
| syn:slr1199 | mutL; DNA mismatch repair protein |
| **syn00650 Butanoate metabolism - *Synechocystis* sp. PCC 6803 (7)** | |
| syn:sll0823 | sdhB; succinate dehydrogenase iron-sulfur subunit (EC:1.3.99.1) |
| syn:sll1625 | sdhB; succinate dehydrogenase iron-sulfur subunit (EC:1.3.99.1) |
| syn:sll1721 | pdhB; Pyruvate dehydrogenase E1 component subunit beta |
| syn:sll1981 | ilvB; acetolactate synthase |
| syn:slr1934 | pyruvate dehydrogenase E1 component subunit alpha |
| syn:slr1993 | thl; acetyl CoA acetyltransferase |
| syn:slr2088 | ilvG; acetolactate synthase 3 catalytic subunit (EC:2.2.1.6) |
| **syn03018 RNA degradation - *Synechocystis* sp. PCC 6803 (6)** | |
| syn:sll0170 | dnaK; molecular chaperone DnaK |
| syn:sll0290 | ppk; polyphosphate kinase (EC:2.7.4.1) |
| syn:sll1043 | pnp; polynucleotide phosphorylase |
| syn:sll1910 | zam; protein conferring resistance to acetazolamide; Zam |
| syn:slr0551 | hypothetical protein |
| syn:slr1129 | rne; ribonuclease E |
| **syn00900 Terpenoid backbone biosynthesis - *Synechocystis* sp. PCC 6803 (6)** | |
| syn:sll0711 | 4-diphosphocytidyl-2-C-methyl-D-erythritol kinase (EC:2.7.1.148) |
| syn:sll1556 | isopentenyl pyrophosphate isomerase (EC:5.3.3.2) |
| syn:sll1945 | 1-deoxy-D-xylulose-5-phosphate synthase (EC:2.2.1.7) |
| syn:slr0611 | sds; solanesyl diphosphate synthase |
| syn:slr1993 | thl; acetyl CoA acetyltransferase |
| syn:slr2136 | ispG; 4-hydroxy-3-methylbut-2-en-1-yl diphosphate synthase (EC:1.17.7.1) |
| **syn00196 Photosynthesis - antenna proteins - *Synechocystis* sp. PCC 6803 (6)** | |
| syn:sll0928 | apcD; allophycocyanin-B |
| syn:sll1051 | cpcF; phycocyanin alpha phycocyanobilin lyase CpcF |
| syn:sll1578 | cpcA; phycocyanin a subunit |
| syn:sll1580 | cpcC; phycocyanin associated linker protein |
| syn:slr0335 | apcE; phycobilisome LCM core-membrane linker polypeptide |
| syn:slr1878 | cpcE; phycocyanin alpha phycocyanobilin lyase CpcE |
| **syn00770 Pantothenate and CoA biosynthesis - *Synechocystis* sp. PCC 6803 (5)** | |
| syn:sll0892 | panD; aspartate alpha-decarboxylase (EC:4.1.1.11) |
| syn:sll1981 | ilvB; acetolactate synthase |
| syn:slr0452 | ilvD; dihydroxy-acid dehydratase (EC:4.2.1.9) |
| syn:slr0847 | coaD; phosphopantetheine adenylyltransferase (EC:2.7.7.3) |
| syn:slr2088 | ilvG; acetolactate synthase 3 catalytic subunit (EC:2.2.1.6) |
| **syn00780 Biotin metabolism - *Synechocystis* sp. PCC 6803 (5)** | |
| syn:sll1605 | fabZ; (3R)-hydroxymyristoyl-ACP dehydratase |
| syn:sll1655 | birA; biotin [acetyl-CoA-carboxylase] ligase |
| syn:sll5079 | hypothetical protein |
| syn:slr0917 | bioF; 8-amino-7-oxononanoate synthase (EC:2.3.1.47) |
| syn:slr1364 | bioB; biotin synthetase |
| **syn00300 Lysine biosynthesis - *Synechocystis* sp. PCC 6803 (5)** | |
| syn:sll0480 | L,L-diaminopimelate aminotransferase |
| syn:sll0504 | lysA; diaminopimelate decarboxylase |
| syn:slr0549 | asd; aspartate beta-semialdehyde dehydrogenese |
| syn:slr0550 | dapA; dihydrodipicolinate synthase (EC:4.2.1.52) |
| syn:slr0657 | lysC; aspartate kinase (EC:2.7.2.4) |
| **syn00660 C5-Branched dibasic acid metabolism - *Synechocystis* sp. PCC 6803 (5)** | |
| syn:sll1023 | sucC; succinate--CoA ligase |
| syn:sll1470 | leuC; isopropylmalate isomerase large subunit (EC:4.2.1.33) |
| syn:sll1557 | sucD; succinyl-CoA synthase subunit beta |
| syn:sll1981 | ilvB; acetolactate synthase |
| syn:slr2088 | ilvG; acetolactate synthase 3 catalytic subunit (EC:2.2.1.6) |
| **syn00400 Phenylalanine, tyrosine and tryptophan biosynthesis - *Synechocystis* sp. PCC 6803 (5)** | |
| syn:sll0402 | aspC; aspartate aminotransferase (EC:2.6.1.1) |
| syn:sll0934 | ccmA; 3-deoxy-7-phosphoheptulonate synthase |
| syn:slr0546 | trpC; indole-3-glycerol phosphate synthase (EC:4.1.1.48) |
| syn:slr0738 | trpE; anthranilate synthase component I |
| syn:slr1867 | trpD; anthranilate phosphoribosyltransferase (EC:2.4.2.18) |
| **syn00710 Carbon fixation in photosynthetic organisms - *Synechocystis* sp. PCC 6803 (5)** | |
| syn:sll0587 | pykF; pyruvate kinase |
| syn:sll1070 | tktA; transketolase (EC:2.2.1.1) |
| syn:slr0009 | rbcL; ribulose bisophosphate carboxylase (EC:4.1.1.39) |
| syn:slr0952 | fbp; fructose-1,6-bisphosphatase (EC:3.1.3.11) |
| syn:slr2094 | glpX; fructose 1,6-bisphosphatase II (EC:3.1.3.11) |
| **syn00550 Peptidoglycan biosynthesis - *Synechocystis* sp. PCC 6803 (5)** | |
| syn:sll1833 | ftsI; penicillin-binding protein |
| syn:slr0017 | murZ; UDP-N-acetylglucosamine 1-carboxyvinyltransferase (EC:2.5.1.7) |
| syn:slr1423 | murC; UDP-N-acetylmuramate--L-alanine ligase (EC:6.3.2.8) |
| syn:slr1424 | murB; UDP-N-acetylenolpyruvoylglucosamine reductase |
| syn:slr1656 | murG; UDP-N-acetylglucosamine-N-acetylmuramyl-(pentape ptide)pyrophosphoryl-undecaprenol N-acetylglucosamine transferase |
| **syn00130 Ubiquinone and other terpenoid-quinone biosynthesis - *Synechocystis* sp. PCC 6803 (5)** | |
| syn:sll0603 | menD; 2-succinyl-5-enolpyruvyl-6-hydroxy-3-cyclohexene-1-carboxylate synthase (EC:2.2.1.9 4.1.1.71) |
| syn:slr0090 | ppd; 4-hydroxyphenylpyruvic acid dioxgenase |
| syn:slr0204 | hypothetical protein |
| syn:slr1300 | ubiH; 2-octaprenyl-6-methoxyphenyl hydroxylase |
| syn:slr1737 | hypothetical protein |
| **syn00521 Streptomycin biosynthesis - *Synechocystis* sp. PCC 6803 (5)** | |
| syn:sll0207 | rfbA; glucose-1-phosphate thymidylyltransferase |
| syn:sll0593 | glk; glucokinase (EC:2.7.1.2) |
| syn:sll1329 | monophosphatase |
| syn:slr0836 | rfbB; dTDP-glucose 4,6-dehydratase |
| syn:slr1933 | rfbC; dTDP-4-dehydrorhamnose 3,5-epimerase |
| **syn00290 Valine, leucine and isoleucine biosynthesis - *Synechocystis* sp. PCC 6803 (4)** | |
| syn:sll1470 | leuC; isopropylmalate isomerase large subunit (EC:4.2.1.33) |
| syn:sll1981 | ilvB; acetolactate synthase |
| syn:slr0452 | ilvD; dihydroxy-acid dehydratase (EC:4.2.1.9) |
| syn:slr2088 | ilvG; acetolactate synthase 3 catalytic subunit (EC:2.2.1.6) |
| **syn03010 Ribosome - *Synechocystis* sp. PCC 6803 (4)** | |
| syn:sll1800 | rplD; 50S ribosomal protein L4 |
| syn:sll1804 | rpsC; 30S ribosomal protein S3 |
| syn:ssl1784 | rpsO; 30S ribosomal protein S15 |
| syn:ssr0482 | rpsP; 30S ribosomal protein S16 |
| **syn00061 Fatty acid biosynthesis - *Synechocystis* sp. PCC 6803 (4)** | |
| syn:sll0728 | accA; acetyl-CoA carboxylase carboxyltransferase subunit alpha (EC:6.4.1.2) |
| syn:sll1605 | fabZ; (3R)-hydroxymyristoyl-ACP dehydratase |
| syn:sll5079 | hypothetical protein |
| syn:slr1511 | fabH; 3-oxoacyl-ACP synthase (EC:2.3.1.41) |
| **syn00051 Fructose and mannose metabolism - *Synechocystis* sp. PCC 6803 (4)** | |
| syn:sll1496 | mannose-1-phosphate guanylyltransferase |
| syn:slr0952 | fbp; fructose-1,6-bisphosphatase (EC:3.1.3.11) |
| syn:slr1448 | cscK; fructokinase |
| syn:slr2094 | glpX; fructose 1,6-bisphosphatase II (EC:3.1.3.11) |
| **syn00380 Tryptophan metabolism - *Synechocystis* sp. PCC 6803 (3)** | |
| syn:sll1987 | katG; catalase HPI |
| syn:slr0091 | aldehyde dehydrogenase |
| syn:slr1993 | thl; acetyl CoA acetyltransferase |
| **syn00790 Folate biosynthesis - *Synechocystis* sp. PCC 6803 (3)** | |
| syn:sll1612 | folC; folyl-polyglutamate synthetase |
| syn:slr0887 | hypothetical protein |
| syn:slr2026 | folP; dihydropteroate pyrophosphorylase |
| **syn01040 Biosynthesis of unsaturated fatty acids - *Synechocystis* sp. PCC 6803 (3)** | |
| syn:sll1441 | desB; delta 15 desaturase |
| syn:sll5079 | hypothetical protein |
| syn:slr1350 | desA; fatty acid desaturase |
| **syn00906 Carotenoid biosynthesis - *Synechocystis* sp. PCC 6803 (3)** | |
| syn:slr0088 | crtO; b-carotene ketolase |
| syn:slr0940 | crtQ; zeta-carotene desaturase |
| syn:slr1254 | pds; phytoene desaturase |
| **syn04122 Sulfur relay system - *Synechocystis* sp. PCC 6803 (3)** | |
| syn:sll0704 | nifS; NifS protein |
| syn:sll0844 | mnmA; tRNA-specific 2-thiouridylase MnmA (EC:2.1.1.61) |
| syn:sll1536 | moeB; hypothetical protein |
| **syn00052 Galactose metabolism - *Synechocystis* sp. PCC 6803 (3)** | |
| syn:sll0244 | galE; UDP-glucose-4-epimerase |
| syn:sll0593 | glk; glucokinase (EC:2.7.1.2) |
| syn:slr1067 | galE; UDP-glucose-4-epimerase |
| **syn03060 Protein export - *Synechocystis* sp. PCC 6803 (3)** | |
| syn:sll0194 | ycf43; hypothetical protein |
| syn:sll0716 | lepB; leader peptidase I |
| syn:slr1531 | ffh; signal recognition particle protein |
| **syn00523 Polyketide sugar unit biosynthesis - *Synechocystis* sp. PCC 6803 (3)** | |
| syn:sll0207 | rfbA; glucose-1-phosphate thymidylyltransferase |
| syn:slr0836 | rfbB; dTDP-glucose 4,6-dehydratase |
| syn:slr1933 | rfbC; dTDP-4-dehydrorhamnose 3,5-epimerase |
| **syn03020 RNA polymerase - *Synechocystis* sp. PCC 6803 (3)** | |
| syn:sll1787 | rpoB; DNA-directed RNA polymerase subunit beta (EC:2.7.7.6) |
| syn:sll1818 | rpoA; DNA-directed RNA polymerase subunit alpha (EC:2.7.7.6) |
| syn:slr1265 | rpoC1; DNA-directed RNA polymerase subunit gamma (EC:2.7.7.6) |
| **syn03030 DNA replication - *Synechocystis* sp. PCC 6803 (3)** | |
| syn:sll0544 | DNA polymerase III subunit delta |
| syn:sll1360 | dnaX; DNA polymerase III subunit |
| syn:slr0603 | dnaE; DNA polymerase III subunit alpha |
| **syn00360 Phenylalanine metabolism - *Synechocystis* sp. PCC 6803 (3)** | |
| syn:sll0402 | aspC; aspartate aminotransferase (EC:2.6.1.1) |
| syn:sll1987 | katG; catalase HPI |
| syn:slr0090 | ppd; 4-hydroxyphenylpyruvic acid dioxgenase |
| **syn00670 One carbon pool by folate - *Synechocystis* sp. PCC 6803 (2)** | |
| syn:slr0212 | metH; 5-methyltetrahydrofolate--homocysteine methyltransferase |
| syn:slr0597 | purH; bifunctional phosphoribosylaminoimidazolecarboxamide formyltransferase/IMP cyclohydrolase (EC:2.1.2.3 3.5.4.10) |
| **syn00280 Valine, leucine and isoleucine degradation - *Synechocystis* sp. PCC 6803 (2)** | |
| syn:slr0091 | aldehyde dehydrogenase |
| syn:slr1993 | thl; acetyl CoA acetyltransferase |
| **syn00564 Glycerophospholipid metabolism - *Synechocystis* sp. PCC 6803 (2)** | |
| syn:sll1085 | glpD; glycerol-3-phosphate dehydrogenase |
| syn:slr0054 | dgkA; diacylglycerol kinase |
| **syn00740 Riboflavin metabolism - *Synechocystis* sp. PCC 6803 (2)** | |
| syn:sll1894 | ribA; bifunctional 3,4-dihydroxy-2-butanone 4-phosphate synthase/GTP cyclohydrolase II (EC:3.5.4.25) |
| syn:slr1882 | ribF; bifunctional riboflavin kinase/FMN adenylyltransferase (EC:2.7.1.26 2.7.7.2) |
| **syn00730 Thiamine metabolism - *Synechocystis* sp. PCC 6803 (2)** | |
| syn:sll0635 | thiE; thiamine-phosphate pyrophosphorylase (EC:2.5.1.3) |
| syn:sll0704 | nifS; NifS protein |
| **syn00410 beta-Alanine metabolism - *Synechocystis* sp. PCC 6803 (2)** | |
| syn:sll0892 | panD; aspartate alpha-decarboxylase (EC:4.1.1.11) |
| syn:slr0091 | aldehyde dehydrogenase |
| **syn00053 Ascorbate and aldarate metabolism - *Synechocystis* sp. PCC 6803 (2)** | |
| syn:slr0091 | aldehyde dehydrogenase |
| syn:slr1299 | UDP-glucose dehydrogenase |
| **syn00750 Vitamin B6 metabolism - *Synechocystis* sp. PCC 6803 (2)** | |
| syn:sll0660 | pdxA; 4-hydroxythreonine-4-phosphate dehydrogenase (EC:1.1.1.262) |
| syn:sll1172 | thrC; threonine synthase (EC:4.2.3.1) |
| **syn00460 Cyanoamino acid metabolism - *Synechocystis* sp. PCC 6803 (2)** | |
| syn:sll0422 | asparaginase |
| syn:sll1538 | bgl; beta-glucosidase |
| **syn00310 Lysine degradation - *Synechocystis* sp. PCC 6803 (2)** | |
| syn:slr0091 | aldehyde dehydrogenase |
| syn:slr1993 | thl; acetyl CoA acetyltransferase |
| **syn00450 Selenocompound metabolism - *Synechocystis* sp. PCC 6803 (2)** | |
| syn:slr0212 | metH; 5-methyltetrahydrofolate--homocysteine methyltransferase |
| syn:slr0649 | metG; methionyl-tRNA synthetase (EC:6.1.1.10) |
| **syn00071 Fatty acid metabolism - *Synechocystis* sp. PCC 6803 (2)** | |
| syn:slr0091 | aldehyde dehydrogenase |
| syn:slr1993 | thl; acetyl CoA acetyltransferase |
| **syn00040 Pentose and glucuronate interconversions - *Synechocystis* sp. PCC 6803 (2)** | |
| syn:slr0091 | aldehyde dehydrogenase |
| syn:slr1299 | UDP-glucose dehydrogenase |
| **syn03420 Nucleotide excision repair - *Synechocystis* sp. PCC 6803 (2)** | |
| syn:sll0865 | uvrC; excinuclease ABC subunit C |
| syn:sll1143 | uvrD; DNA helicase II |
| **syn00920 Sulfur metabolism - *Synechocystis* sp. PCC 6803 (2)** | |
| syn:sll0712 | cysM; cysteine synthase A |
| syn:sll0895 | cysQ; ammonium transporter |
| **syn00623 Toluene degradation - *Synechocystis* sp. PCC 6803 (2)** | |
| syn:sll0823 | sdhB; succinate dehydrogenase iron-sulfur subunit (EC:1.3.99.1) |
| syn:sll1625 | sdhB; succinate dehydrogenase iron-sulfur subunit (EC:1.3.99.1) |
| **syn00471 D-Glutamine and D-glutamate metabolism - *Synechocystis* sp. PCC 6803 (2)** | |
| syn:slr1423 | murC; UDP-N-acetylmuramate--L-alanine ligase (EC:6.3.2.8) |
| syn:slr1746 | murI; glutamate racemase (EC:5.1.1.3) |
| **syn00561 Glycerolipid metabolism - *Synechocystis* sp. PCC 6803 (2)** | |
| syn:slr0054 | dgkA; diacylglycerol kinase |
| syn:slr0091 | aldehyde dehydrogenase |
| **syn00350 Tyrosine metabolism - *Synechocystis* sp. PCC 6803 (2)** | |
| syn:sll0402 | aspC; aspartate aminotransferase (EC:2.6.1.1) |
| syn:slr0090 | ppd; 4-hydroxyphenylpyruvic acid dioxgenase |
| **syn03070 Bacterial secretion system - *Synechocystis* sp. PCC 6803 (2)** | |
| syn:sll0194 | ycf43; hypothetical protein |
| syn:slr1531 | ffh; signal recognition particle protein |
| **syn00540 Lipopolysaccharide biosynthesis - *Synechocystis* sp. PCC 6803 (1)** | |
| syn:slr0862 | lmbP; LmbP protein |
| **syn00401 Novobiocin biosynthesis - *Synechocystis* sp. PCC 6803 (1)** | |
| syn:sll0402 | aspC; aspartate aminotransferase (EC:2.6.1.1) |
| **syn03410 Base excision repair - *Synechocystis* sp. PCC 6803 (1)** | |
| syn:sll1354 | recJ; ssDNA-specific exonuclease RecJ |
| **syn00590 Arachidonic acid metabolism - *Synechocystis* sp. PCC 6803 (1)** | |
| syn:slr1992 | glutathione peroxidase |
| **syn00909 Sesquiterpenoid and triterpenoid biosynthesis - *Synechocystis* sp. PCC 6803 (1)** | |
| syn:slr2089 | shc; squalene-hopene cyclase |
| **syn00430 Taurine and hypotaurine metabolism - *Synechocystis* sp. PCC 6803 (1)** | |
| syn:slr2132 | pta; phosphate acetyltransferase (EC:2.3.1.8) |
| **syn00625 Chloroalkane and chloroalkene degradation - *Synechocystis* sp. PCC 6803 (1)** | |
| syn:slr0091 | aldehyde dehydrogenase |
| **syn00362 Benzoate degradation - *Synechocystis* sp. PCC 6803 (1)** | |
| syn:slr1993 | thl; acetyl CoA acetyltransferase |
| **syn00562 Inositol phosphate metabolism - *Synechocystis* sp. PCC 6803 (1)** | |
| syn:sll1329 | monophosphatase |
| **syn00903 Limonene and pinene degradation - *Synechocystis* sp. PCC 6803 (1)** | |
| syn:slr0091 | aldehyde dehydrogenase |

| **Table S7.** List of the primer oligonucleotides used in this study. | | | | | | |
| --- | --- | --- | --- | --- | --- | --- |
| **Name** | **Location** | **Start(nt)** | **End(nt)** | **Strand** | | **5'primer (5'-3')** |
| SYPCC_Igr_01 | pSYSA | 68081 | 68925 | + | GTGTCTATCCCGACAAAATTAGGG | |
| SYPCC_Igr_02 | chromesome | 2530895 | 2530962 | + | ATTCCCTTCTCCAACCAATTTAGTA | |
| SYPCC_Igr_03 | pSYSG | 15392 | 15481 | + | AACGAAAGACCTCCCTCCAGG | |
| SYPCC_Igr_04 | chromesome | 496101 | 496321 | + | TGACTCCAAAGAATAGGCTATAGTTAA | |
| SYPCC_Igr_05 | chromesome | 1104709 | 1104929 | + | TTGCTCCCTAAATTTCCTCGC | |
| SYPCC_Igr_06 | chromesome | 3019919 | 3020058 | - | CTCTAAATCTCTTCATTAAAGCTCCG | |
| SYPCC_Igr_07 | pSYSM | 66503 | 66574 | + | TTCCACAGTGTTCACGCTTTTG | |
| SYPCC_Igr_08 | chromesome | 3039292 | 3039406 | + | TTTAAGATGGCGAATCAAATGATTC | |
| SYPCC_Igr_09 | chromesome | 3305423 | 3305514 | + | ATTTATCCCCCTAAATCTCCTCACT | |
| SYPCC_anti_01 | chromesome | 407802 | 407923 | + | GCTAATGCTATAGGTCACATCCAA | |
| SYPCC_anti_02 | chromesome | 1909216 | 1909443 | - | AATTCCTTTACACTGGGGGTCTAA | |
| SYPCC_anti_03 | chromesome | 2724266 | 2724365 | - | CCACTACGTTTTTAACTTTACTAAAAC | |
| SYPCC_anti_04 | chromesome | 279064 | 29161 | - | ATATTTAACCGTGTCCACATCGG | |
| SYPCC_anti_05 | chromesome | 346136 | 346282 | + | GTGGTAGATTTTCCATCACCGG | |
| SYPCC_anti_06 | chromesome | 2478723 | 2478824 | - | CAACGGGAAAACGAATATCTTC | |
| SYPCC_anti_07 | chromesome | 940674 | 940896 | + | AGCAGTGCTCGACATTGCAA | |
| SYPCC_anti_08 | chromesome | 1665302 | 1665520 | + | AAAATTACCTGGGGATGCCA | |
| SYPCC_anti_09 | chromesome | 2219904 | 2220106 | - | CCCACACCTGGAGTAAATTTTTT | |
| SYPCC_anti_10 | chromesome | 1020080 | 1020302 | - | TTAATCTTTCCTCTGCTCCTTCA | |
| SYPCC_anti_11 | pSYSX | 91564 | 91738 | - | AATTTACCTCGATCAACAGTAGCTAA | |
| SYPCC_Ir_01 | chromesome | 1832226 | 1832339 | + | TTAGAAATGGCTCGGACTCATGC | |
| SYPCC_Ir_02 | chromesome | 2730512 | 2730591 | + | ATTATCTAGAGGTGTGTGAGGAGTAAG | |
| SYPCC_Ir_03 | chromesome | 3433172 | 3433275 | + | TTCAAACTTTGTAAACAGGCCG | |
| SYPCC_Ir_04 | chromesome | 901005 | 901125 | + | CCTTCTCTGGTCAGTCCTACGG | |
| SYPCC_Ir_05 | chromesome | 458917 | 459162 | + | TACTTCCGTCTTCTTCAAAAAGAGA | |
| SYPCC_Ir_06 | chromesome | 3168882 | 3169030 | + | TCCCCTTGGATTAGTCATTGTCC | |
| SYPCC_Ir_07 | chromesome | 102301 | 102387 | - | ATTGCAATTTAGGAACTTACTTCCA | |
| 5S-forward |  |  |  |  | TGGTGTCTTTAGCGTCATGGAAC | |
| 5S-reverse |  |  |  |  | ACTTGGCATCGGACTATTGTG | |
